# Supplementary material for: Blood pro-resolving mediators are linked with synovial pathology and are predictive of DMARD responsiveness in rheumatoid arthritis
Source: Nat Commun. 2020 Oct 27;11:5420. doi: 10.1038/s41467-020-19176-z (PMC7591509; doi:10.1038/s41467-020-19176-z)
Supplement: Supplementary file 2 — Supplementary Information [file 41467_2020_19176_MOESM2_ESM.pdf]

Blood pro-resolving mediators are linked with synovial pathology and are predictive of DMARD responsiveness in rheumatoid arthritis

Esteban A. Gomez[1*], Romain A. Colas[1*], Patricia R. Souza[1*], Rebecca Hands[2], Myles J. Lewis[2], Conrad Bessant[3], Costantino Pitzalis[2,4,$] and Jesmond Dalli[1,4,$+].

[1] William Harvey Research Institute, Barts and The London School of Medicine and Dentistry, Queen Mary University of London, Charterhouse Square, London, EC1M 6BQ, UK.

[2] Centre for Experimental Medicine & Rheumatology, William Harvey Research Institute, Barts & The London School of Medicine & Dentistry, Queen Mary University of London, London, UK

[3] School of Biological and Chemical Sciences, Queen Mary University of London, Mile End Road, London E1 4NS, UK.

[4] Centre for Inflammation and Therapeutic Innovation, Queen Mary University of London, London, UK.

* These authors contributed equally

[$] These authors jointly supervised this work

[+]Corresponding author: Dr Jesmond Dalli Ph.D, William Harvey Research Institute, John Vane Science Centre, Charterhouse Square, London. EC1M 6BQ. E-mail: j.dalli@qmul.ac.uk, Tel: +44 (0) 207 882 8263

Supplementary Figures and Legends

A

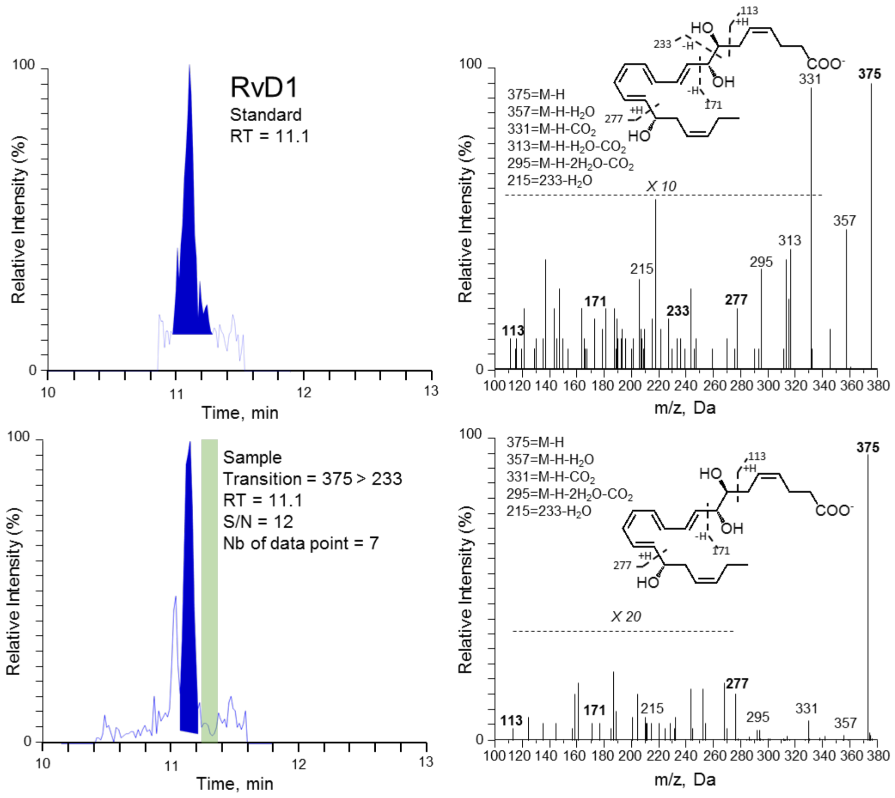

B

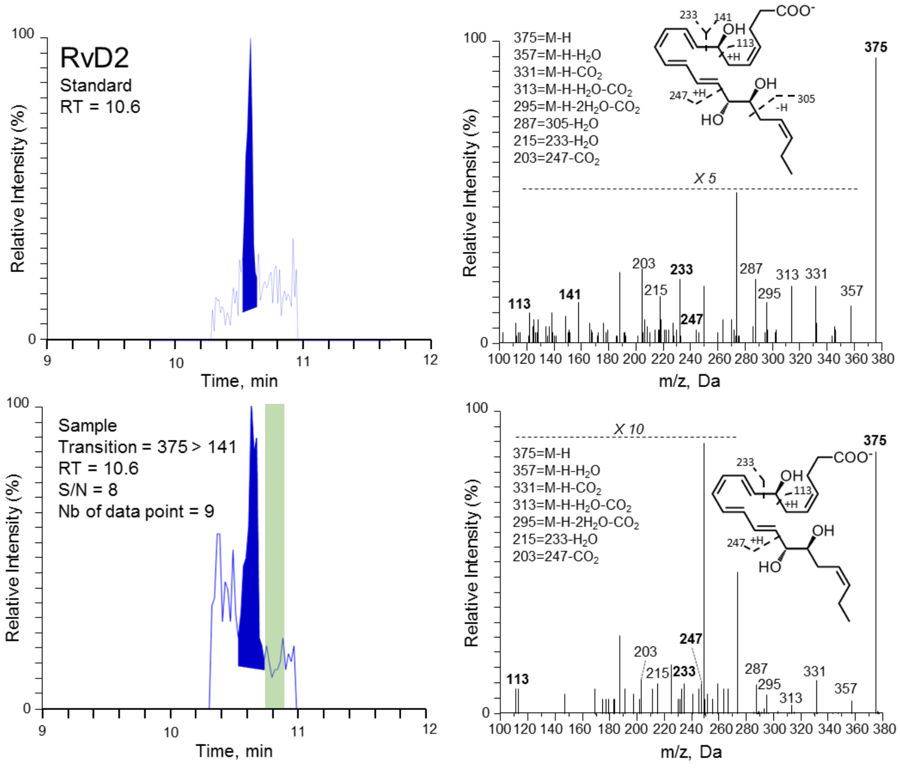

C

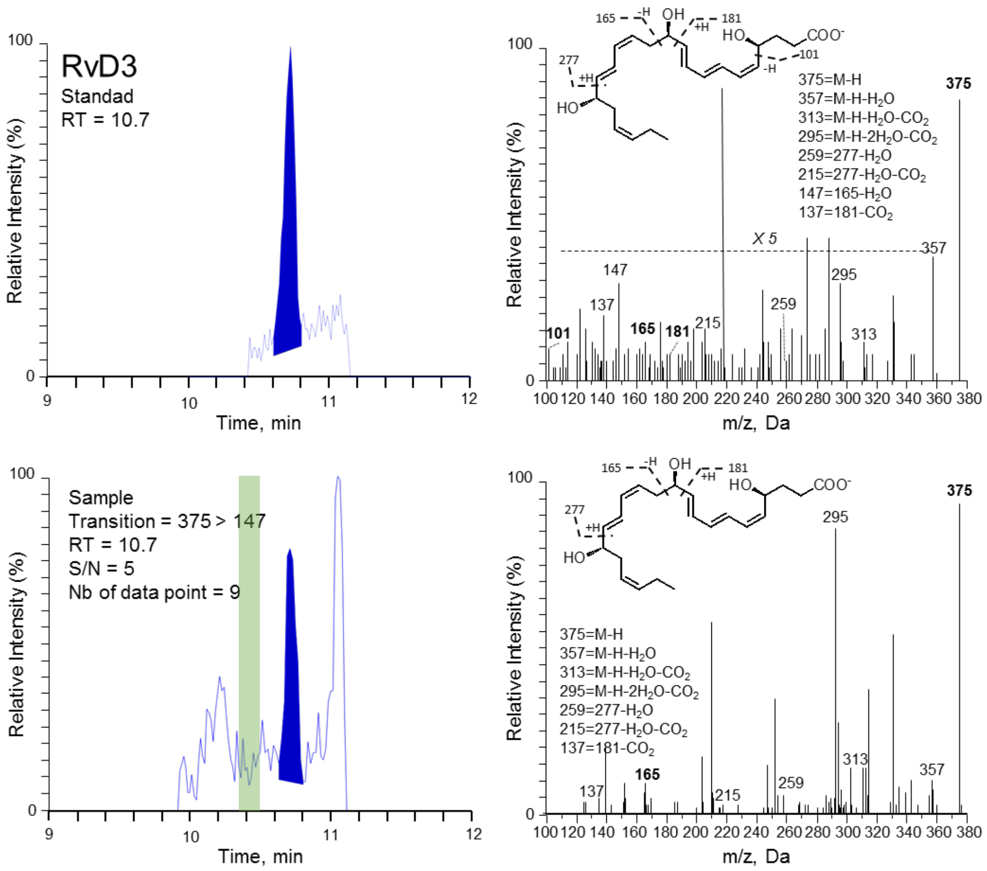
RvD3
Standad
RT = 10.7
Relative Intensity (%)
Time, min
Sample
Transition = 375 > 147
RT = 10.7
S/N = 5
Nb of data point = 9
m/z, Da
375=M-H
357=M-H-H2O
313=M-H-H2O-CO2
295=M-H-2H2O-CO2
259=277-H2O
215=277-H2O-CO2
147=165-H2O
137=181-CO2
X 5
COO-
HO
OH

D

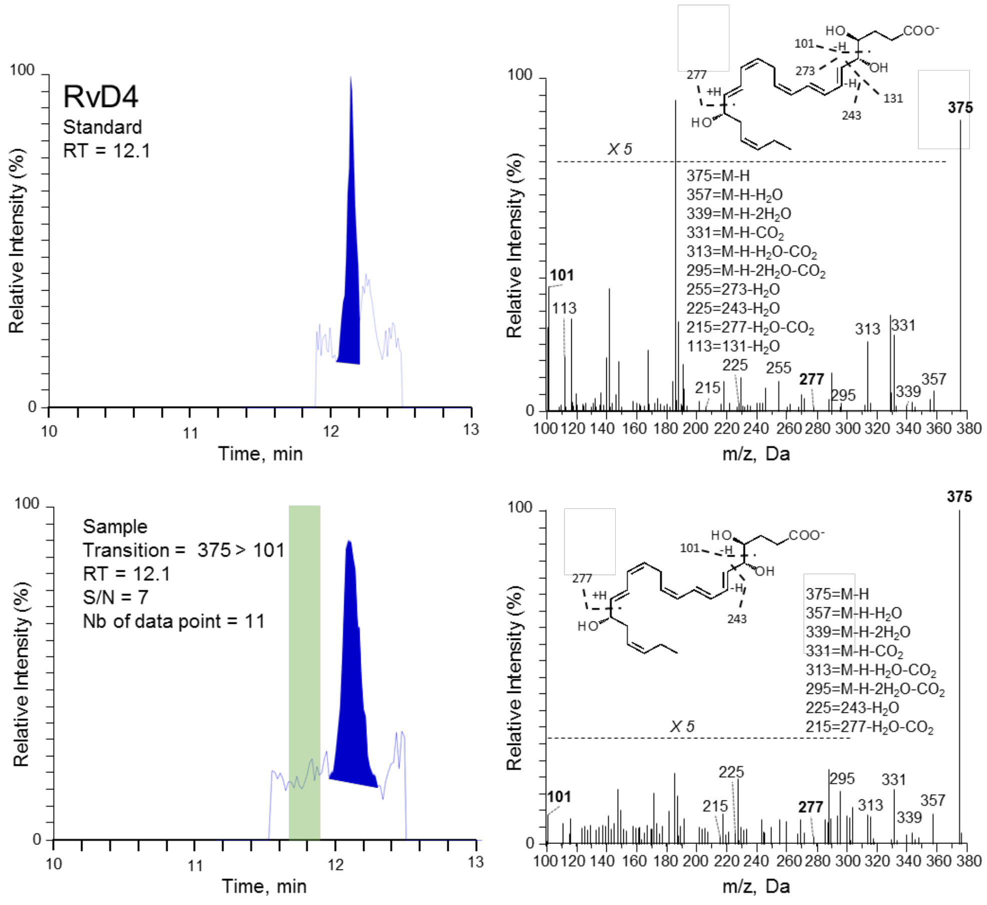
RvD4
Standard
RT = 12.1
Relative Intensity (%)
Time, min
Sample
Transition = 375 > 101
RT = 12.1
S/N = 7
Nb of data point = 11
m/z, Da
375=M-H
357=M-H-H2O
339=M-H-2H2O
331=M-H-CO2
313=M-H-H2O-CO2
295=M-H-2H2O-CO2
255=273-H2O
225=243-H2O
215=277-H2O-CO2
113=131-H2O
X 5
COO-
HO
OH

E

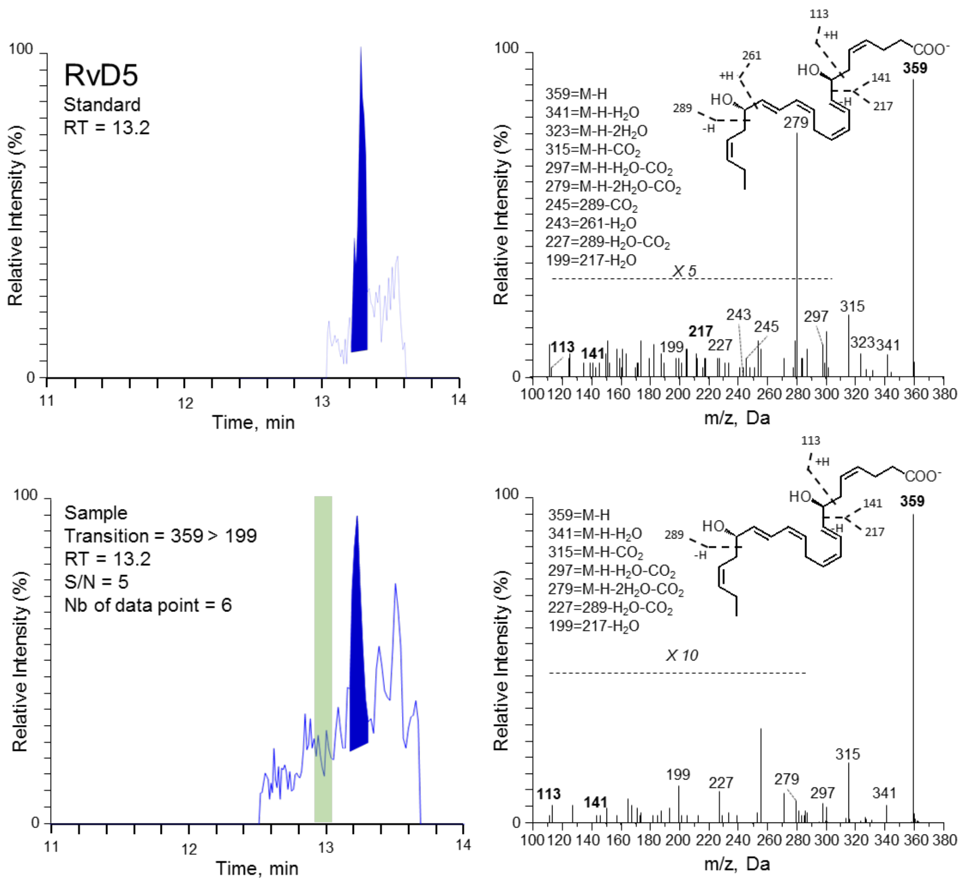

RvD5
Standard
RT = 13.2
Relative Intensity (%)
Time, min
359=M-H
341=M-H-H2O
323=M-H-2H2O
315=M-H-CO2
297=M-H-H2O-CO2
279=M-H-2H2O-CO2
245=289-CO2
243=261-H2O
227=289-H2O-CO2
199=217-H2O
X 5
m/z, Da
Sample
Transition = 359 > 199
RT = 13.2
S/N = 5
Nb of data point = 6
359=M-H
341=M-H-H2O
315=M-H-CO2
297=M-H-H2O-CO2
279=M-H-2H2O-CO2
227=289-H2O-CO2
199=217-H2O
X 10

F

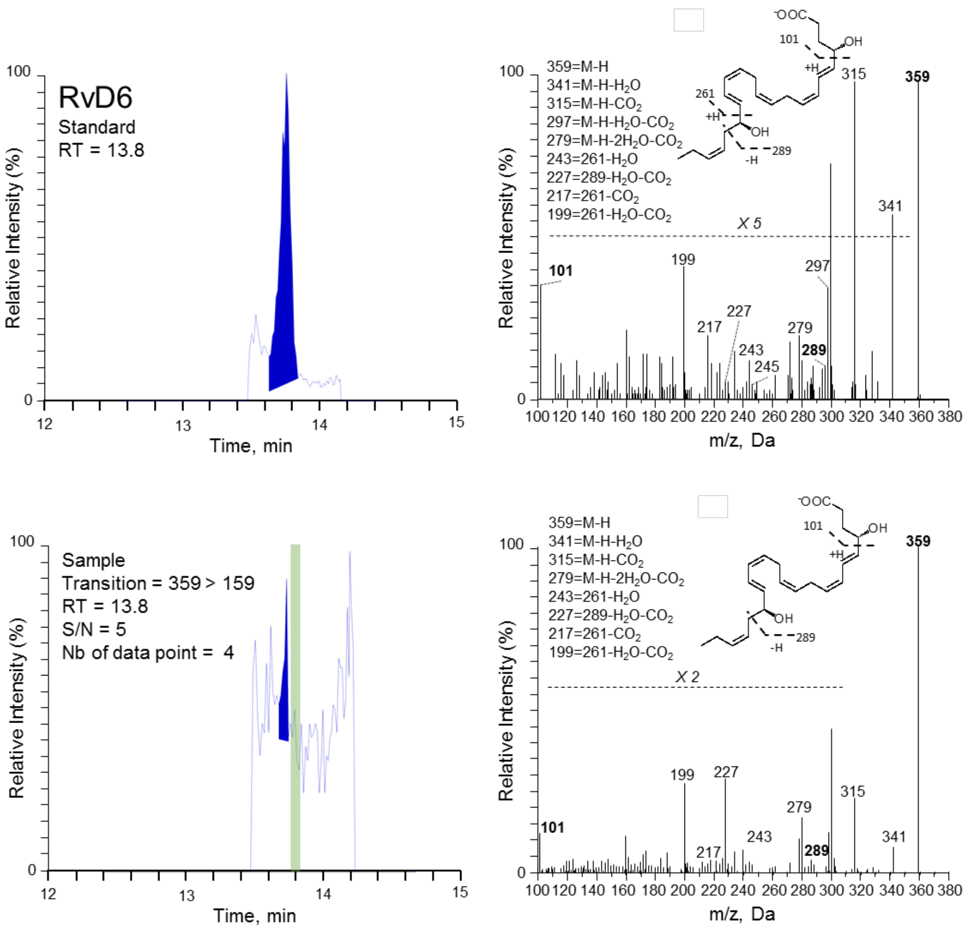

RvD6
Standard
RT = 13.8
Relative Intensity (%)
Time, min
359=M-H
341=M-H-H2O
315=M-H-CO2
297=M-H-H2O-CO2
279=M-H-2H2O-CO2
243=261-H2O
227=289-H2O-CO2
217=261-CO2
199=261-H2O-CO2
X 5
m/z, Da
Sample
Transition = 359 > 159
RT = 13.8
S/N = 5
Nb of data point = 4
359=M-H
341=M-H-H2O
315=M-H-CO2
279=M-H-2H2O-CO2
243=261-H2O
227=289-H2O-CO2
217=261-CO2
199=261-H2O-CO2
X 2

G

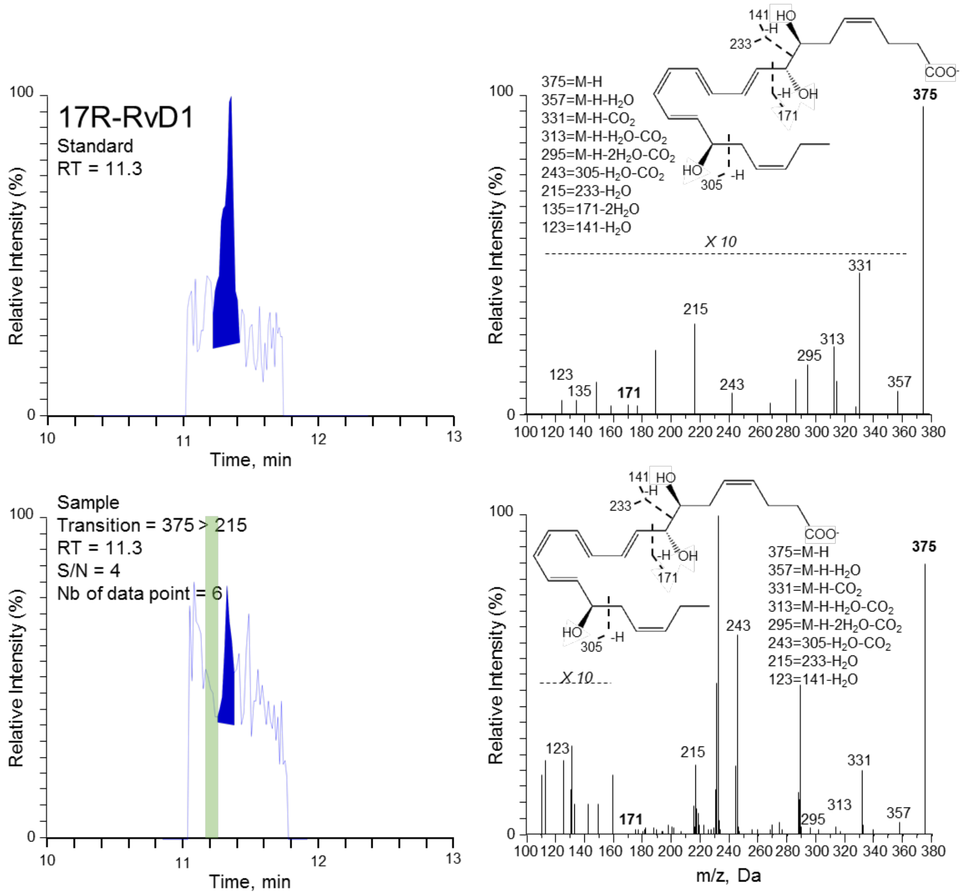
17R-RvD1
Standard
RT = 11.3
Relative Intensity (%)
Time, min
Sample
Transition = 375 > 215
RT = 11.3
S/N = 4
Nb of data point = 6
375=M-H
357=M-H-H_2O
331=M-H-CO_2
313=M-H-H_2O-CO_2
295=M-H-2H_2O-CO_2
243=305-H_2O-CO_2
215=233-H_2O
135=171-2H_2O
123=141-H_2O
X 10
m/z, Da

H

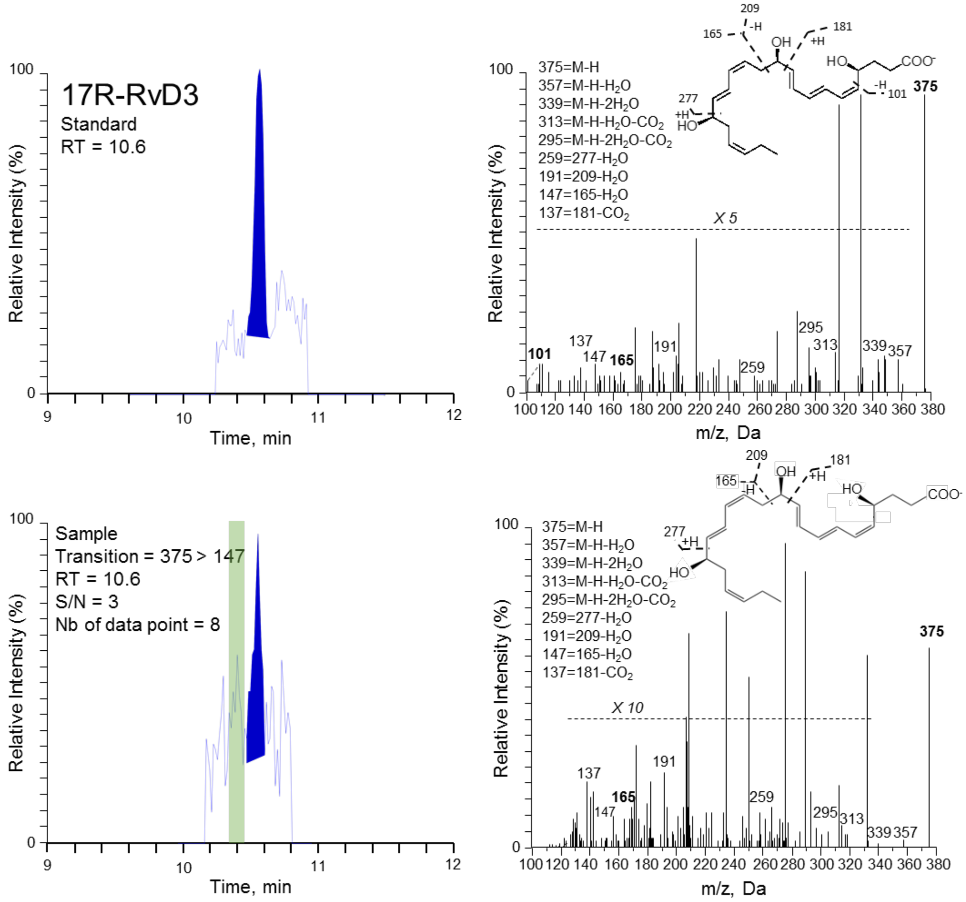
17R-RvD3
Standard
RT = 10.6
Relative Intensity (%)
Time, min
Sample
Transition = 375 > 147
RT = 10.6
S/N = 3
Nb of data point = 8
375=M-H
357=M-H-H_2O
339=M-H-2H_2O
313=M-H-H_2O-CO_2
295=M-H-2H_2O-CO_2
259=277-H_2O
191=209-H_2O
147=165-H_2O
137=181-CO_2
X 5
X 10
m/z, Da

I

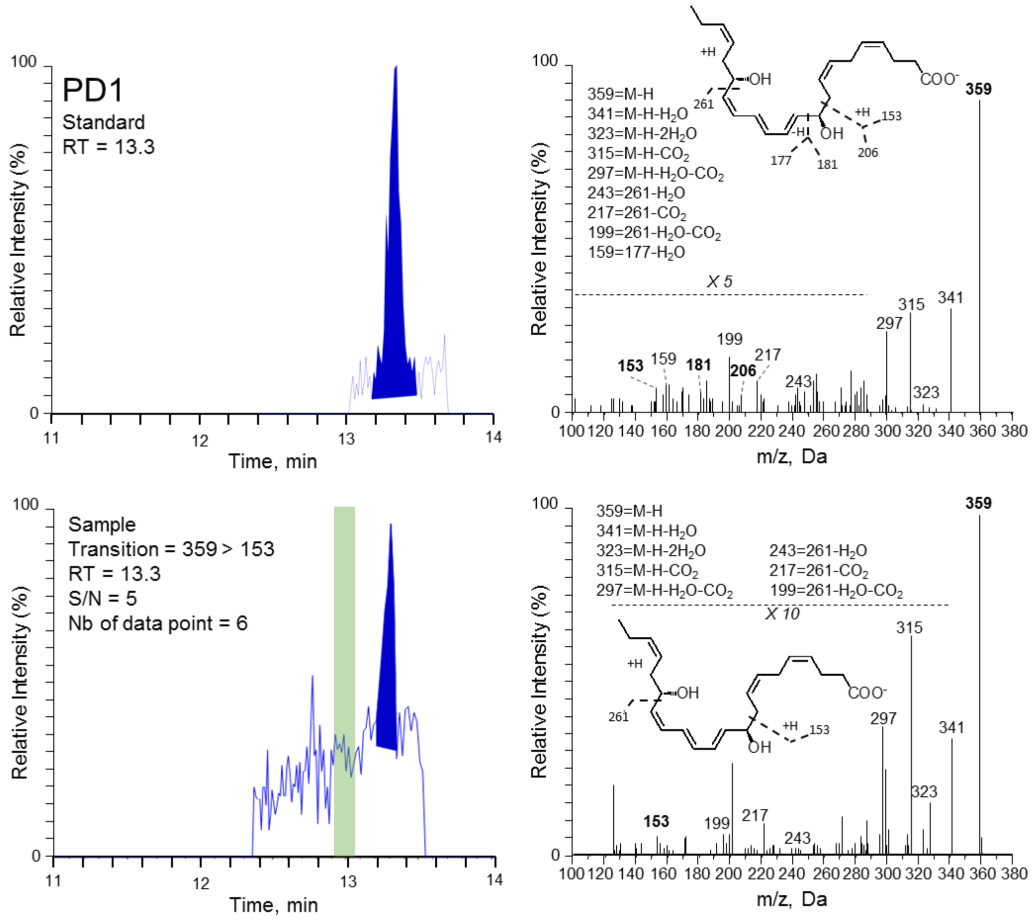
PD1
Standard
RT = 13.3
Relative Intensity (%)
Time, min
359=M-H
341=M-H-H2O
323=M-H-2H2O
315=M-H-CO2
297=M-H-H2O-CO2
243=261-H2O
217=261-CO2
199=261-H2O-CO2
159=177-H2O
X 5
m/z, Da
Sample
Transition = 359 > 153
RT = 13.3
S/N = 5
Nb of data point = 6
X 10

J

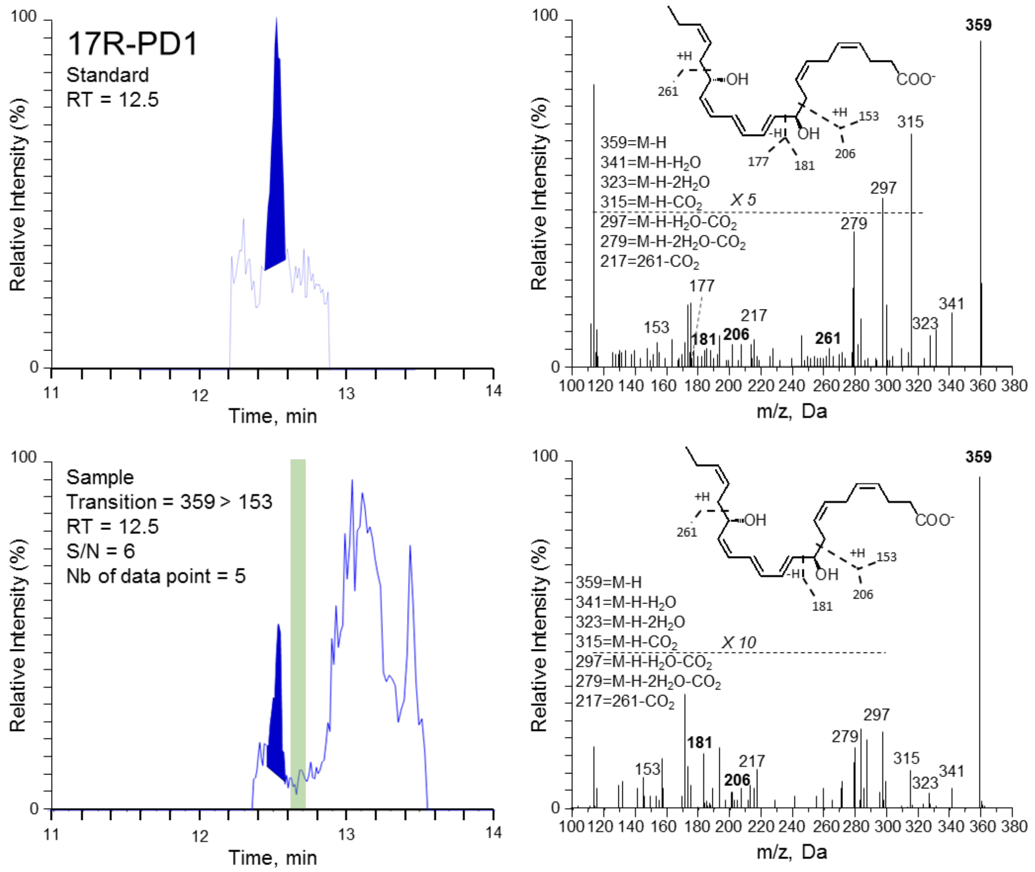
17R-PD1
Standard
RT = 12.5
Relative Intensity (%)
Time, min
359=M-H
341=M-H-H2O
323=M-H-2H2O
315=M-H-CO2
297=M-H-H2O-CO2
279=M-H-2H2O-CO2
217=261-CO2
X 5
m/z, Da
Sample
Transition = 359 > 153
RT = 12.5
S/N = 6
Nb of data point = 5
X 10

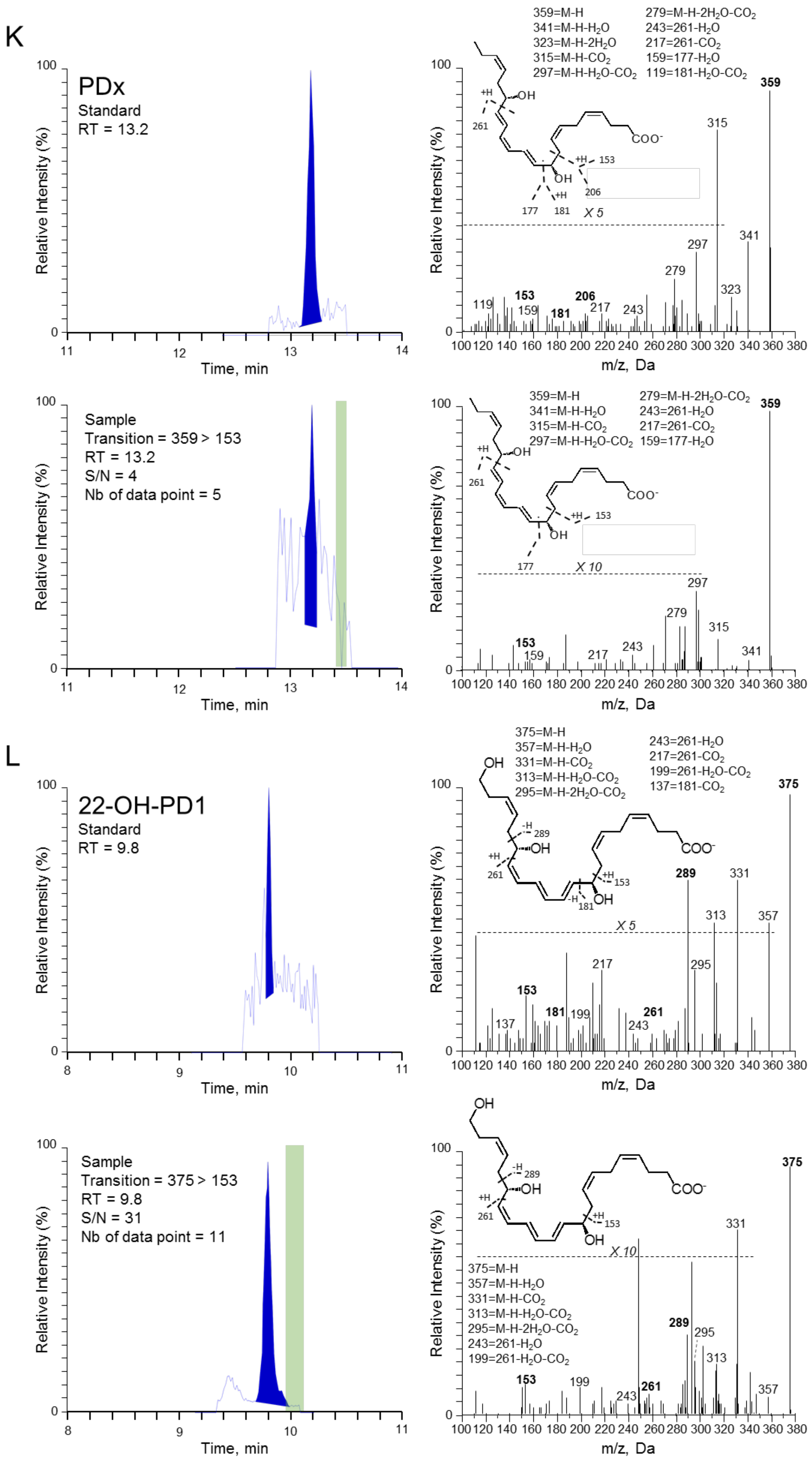
K
PDx
Standard
RT = 13.2
Relative Intensity (%)
Time, min
359=M-H
341=M-H-H_2O
323=M-H-2H_2O
315=M-H-CO_2
297=M-H-H_2O-CO_2
279=M-H-2H_2O-CO_2
243=261-H_2O
217=261-CO_2
159=177-H_2O
119=181-H_2O-CO_2
X 5
m/z, Da
Sample
Transition = 359 > 153
RT = 13.2
S/N = 4
Nb of data point = 5
X 10
L
22-OH-PD1
Standard
RT = 9.8
375=M-H
357=M-H-H_2O
331=M-H-CO_2
313=M-H-H_2O-CO_2
295=M-H-2H_2O-CO_2
243=261-H_2O
217=261-CO_2
199=261-H_2O-CO_2
137=181-CO_2
Sample
Transition = 375 > 153
RT = 9.8
S/N = 31
Nb of data point = 11

M

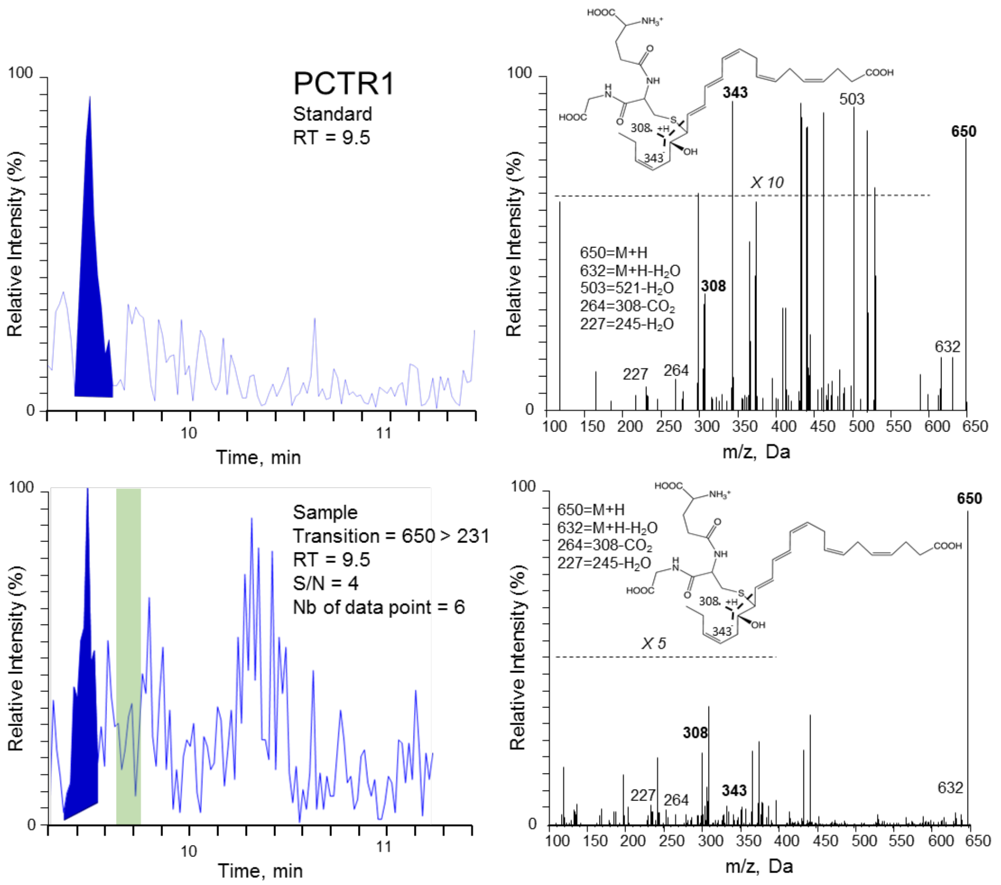
PCTR1
Standard
RT = 9.5
Relative Intensity (%)
Time, min
650=M+H
632=M+H-H2O
503=521-H2O
264=308-CO2
227=245-H2O
X 10
m/z, Da
Sample
Transition = 650 > 231
RT = 9.5
S/N = 4
Nb of data point = 6
X 5

N

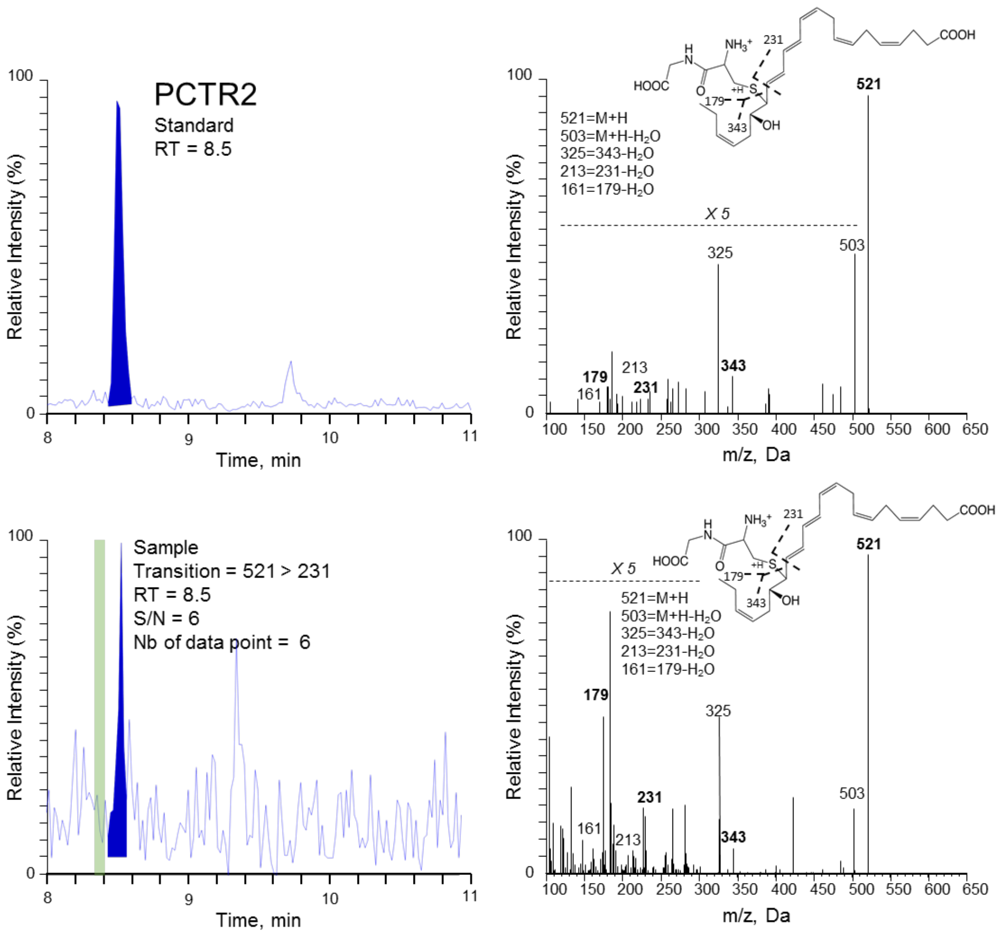
PCTR2
Standard
RT = 8.5
Relative Intensity (%)
Time, min
521=M+H
503=M+H-H2O
325=343-H2O
213=231-H2O
161=179-H2O
X 5
m/z, Da
Sample
Transition = 521 > 231
RT = 8.5
S/N = 6
Nb of data point = 6

O

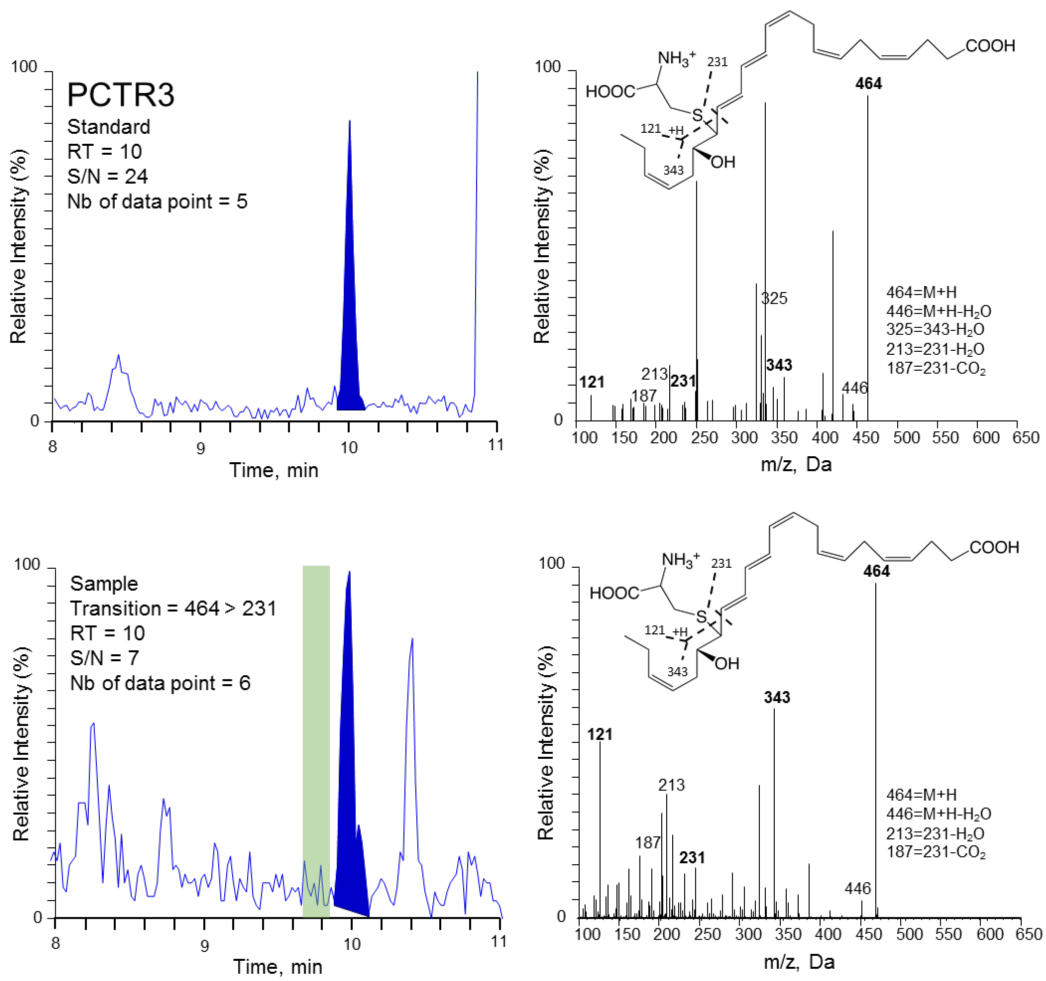
PCTR3
Standard
RT = 10
S/N = 24
Nb of data point = 5
Relative Intensity (%)
Time, min
464
325
343
121
213
231
187
446
464=M+H
446=M+H-H2O
325=343-H2O
213=231-H2O
187=231-CO2
m/z, Da
Sample
Transition = 464 > 231
RT = 10
S/N = 7
Nb of data point = 6
464=M+H
446=M+H-H2O
213=231-H2O
187=231-CO2
NH3+
HOOC
COOH
OH

P

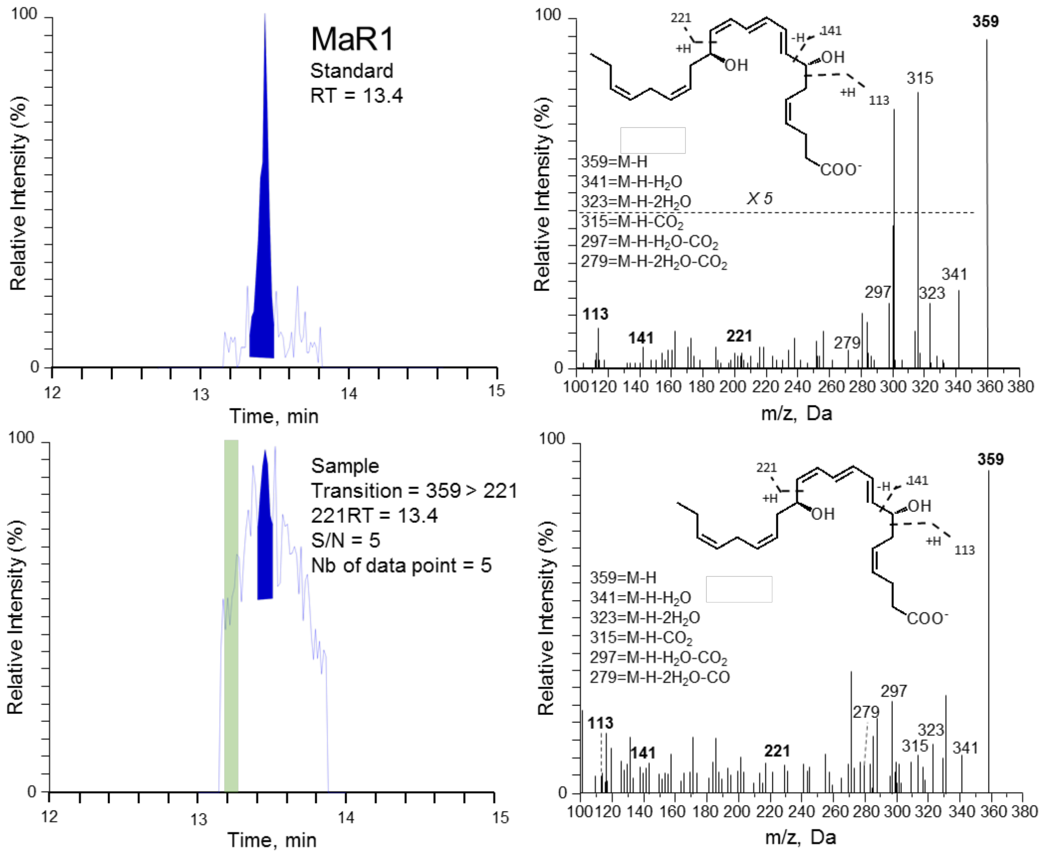
MaR1
Standard
RT = 13.4
Relative Intensity (%)
Time, min
359=M-H
341=M-H-H2O
323=M-H-2H2O
315=M-H-CO2
297=M-H-H2O-CO2
279=M-H-2H2O-CO2
X 5
m/z, Da
Sample
Transition = 359 > 221
221RT = 13.4
S/N = 5
Nb of data point = 5
279=M-H-2H2O-CO
COO-
OH

Q

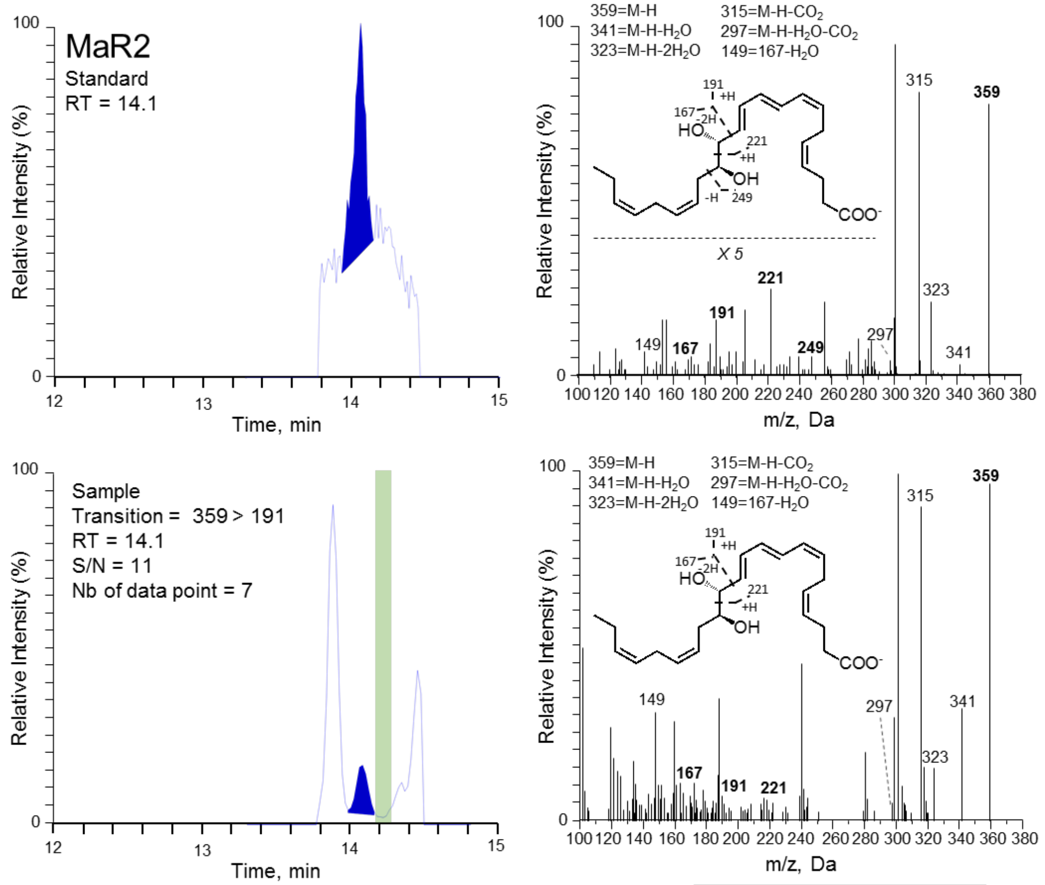
MaR2
Standard
RT = 14.1
Relative Intensity (%)
Time, min
359=M-H
341=M-H-H_2O
323=M-H-2H_2O
315=M-H-CO_2
297=M-H-H_2O-CO_2
149=167-H_2O
X 5
m/z, Da
Sample
Transition = 359 > 191
RT = 14.1
S/N = 11
Nb of data point = 7

R

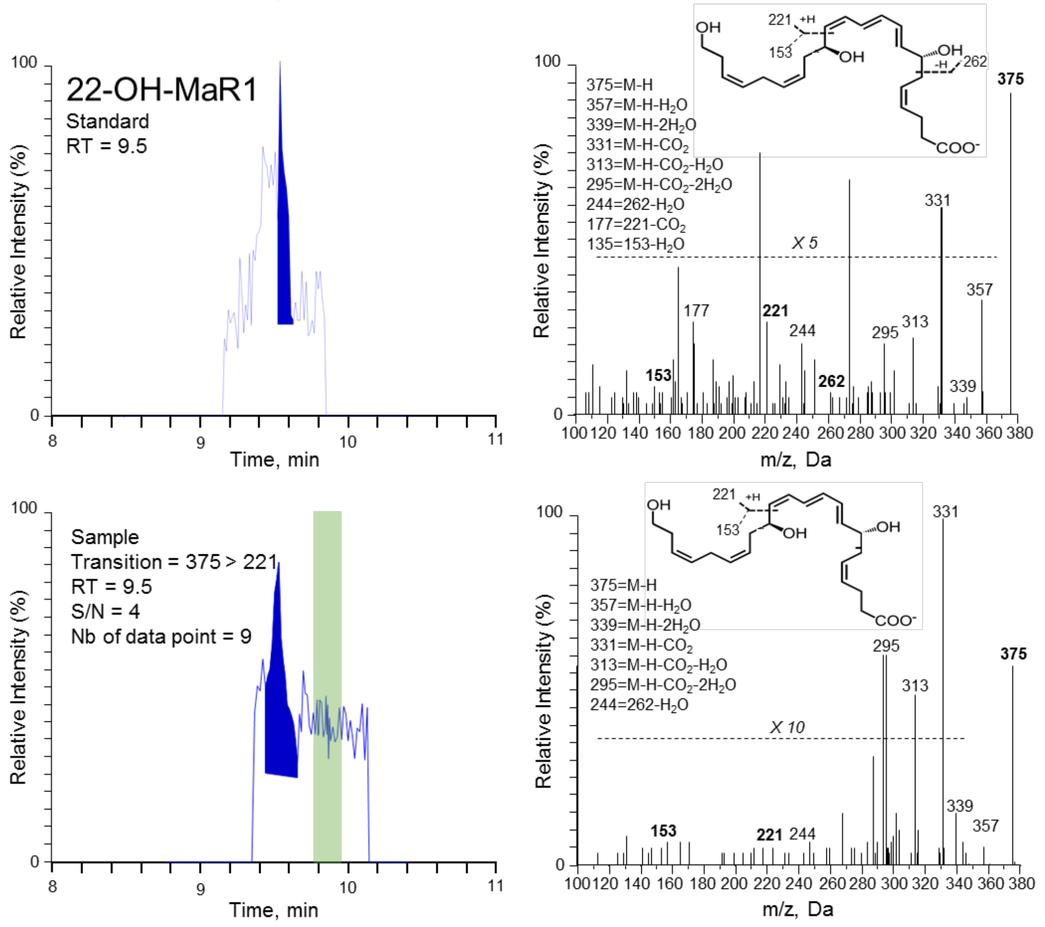
22-OH-MaR1
Standard
RT = 9.5
Relative Intensity (%)
Time, min
375=M-H
357=M-H-H_2O
339=M-H-2H_2O
331=M-H-CO_2
313=M-H-CO_2-H_2O
295=M-H-CO_2-2H_2O
244=262-H_2O
177=221-CO_2
135=153-H_2O
X 5
m/z, Da
Sample
Transition = 375 > 221
RT = 9.5
S/N = 4
Nb of data point = 9
X 10

S

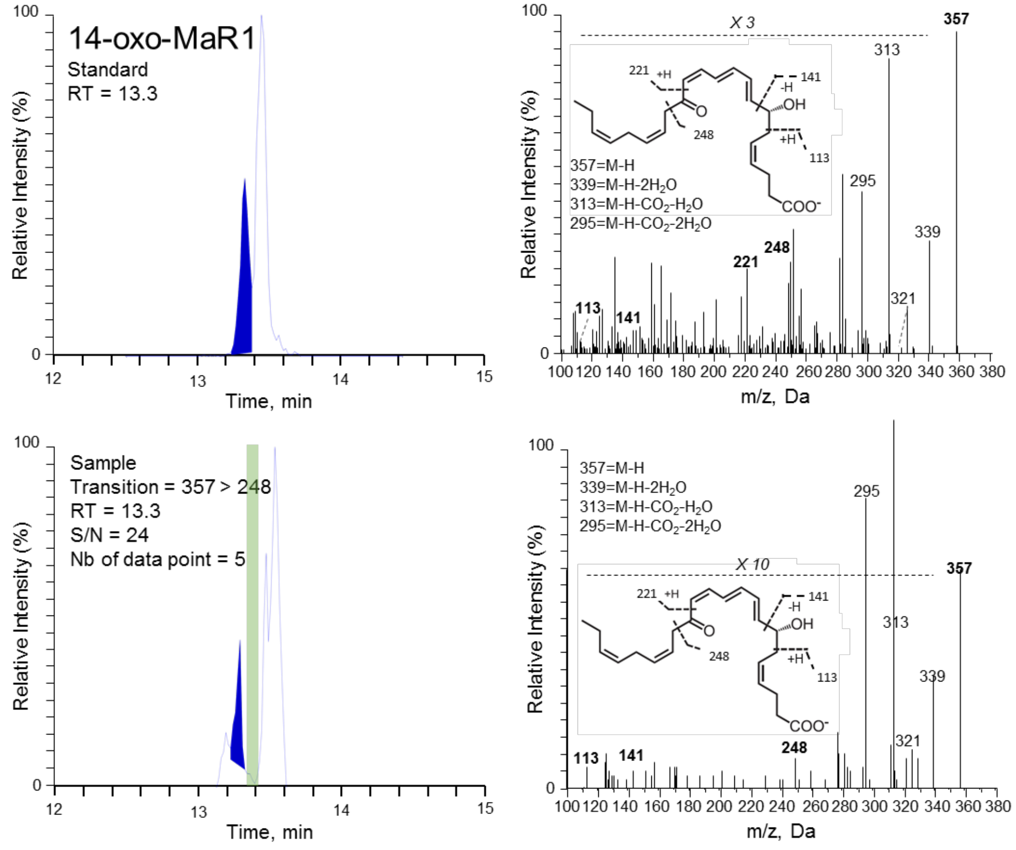
14-oxo-MaR1
Standard
RT = 13.3
Relative Intensity (%)
Time, min
X 3
357=M-H
339=M-H-2H2O
313=M-H-CO2-H2O
295=M-H-CO2-2H2O
m/z, Da
Sample
Transition = 357 > 248
RT = 13.3
S/N = 24
Nb of data point = 5
X 10

T

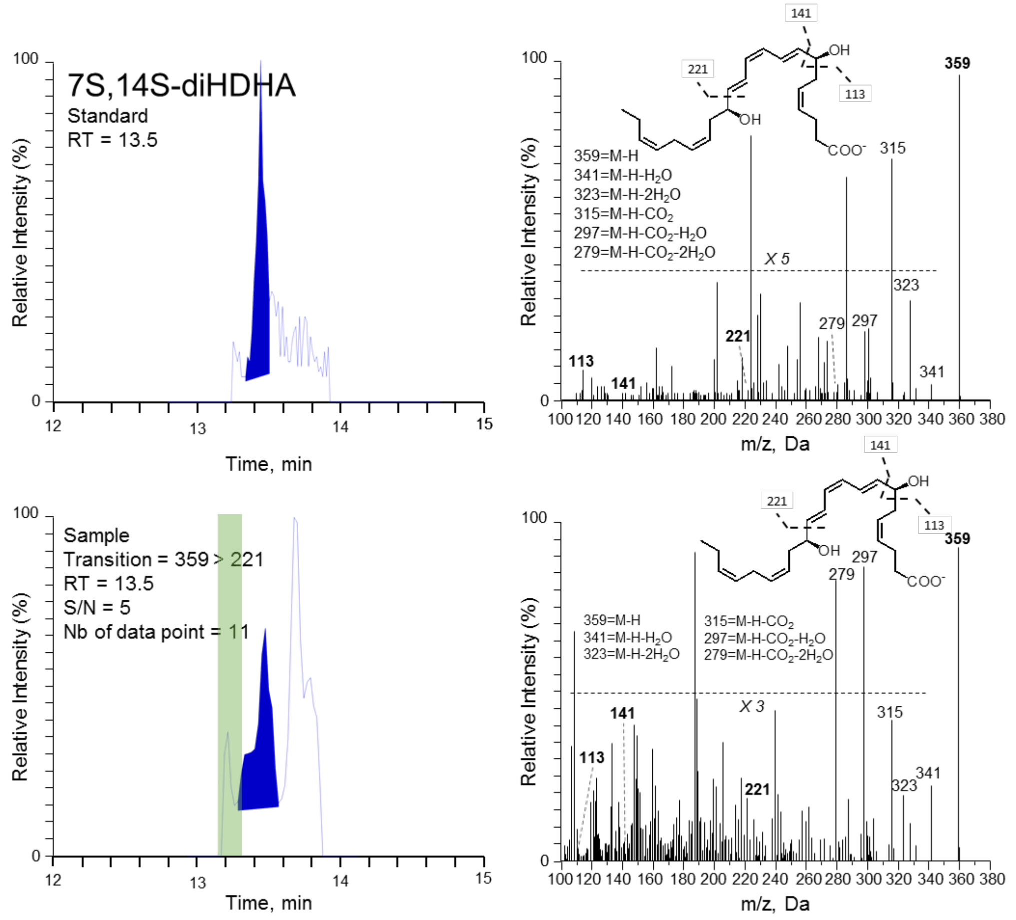
7S,14S-diHDHA
Standard
RT = 13.5
Relative Intensity (%)
Time, min
359=M-H
341=M-H-H2O
323=M-H-2H2O
315=M-H-CO2
297=M-H-CO2-H2O
279=M-H-CO2-2H2O
X 5
m/z, Da
Sample
Transition = 359 > 221
RT = 13.5
S/N = 5
Nb of data point = 11
X 3

U

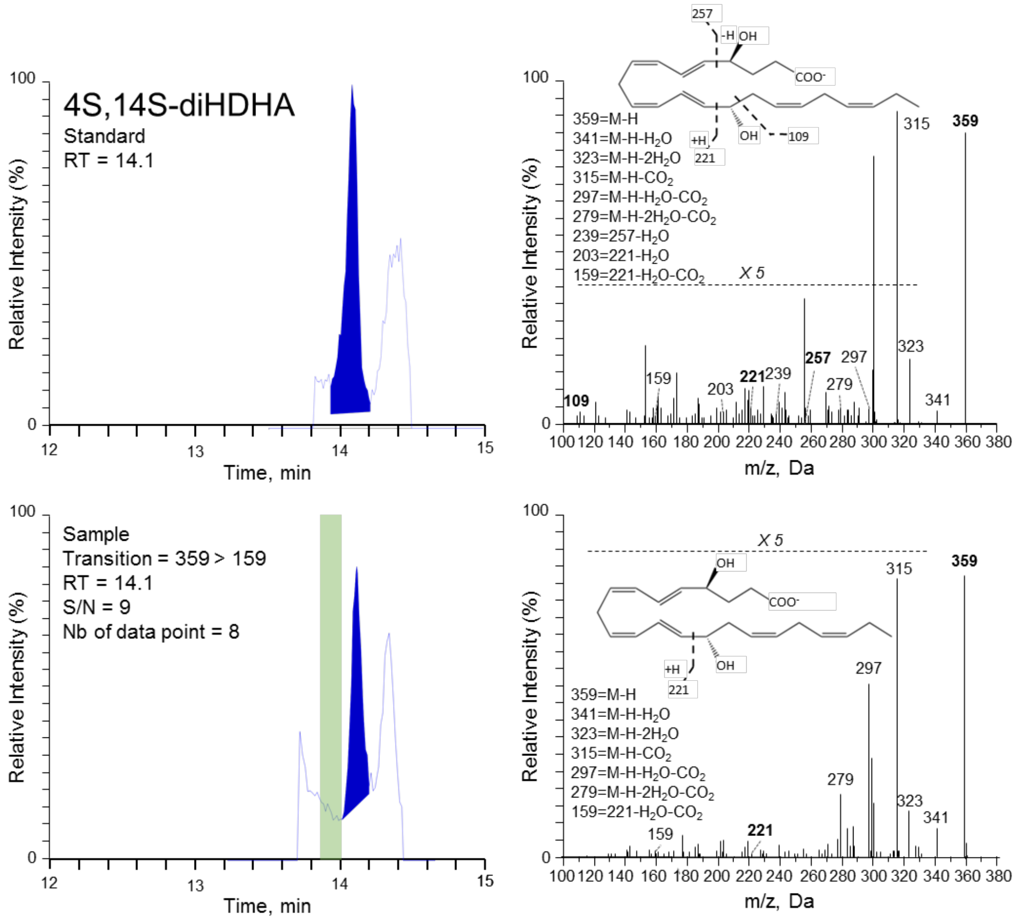
4S,14S-diHDHA
Standard
RT = 14.1
Relative Intensity (%)
Time, min
359=M-H
341=M-H-H_2O
323=M-H-$2H_2O$
315=M-H-CO_2
297=M-H-H_2O-CO_2
279=M-H-$2H_2O$-CO_2
239=257-H_2O
203=221-H_2O
159=221-H_2O-CO_2
X 5
m/z, Da
Sample
Transition = 359 > 159
RT = 14.1
S/N = 9
Nb of data point = 8

V

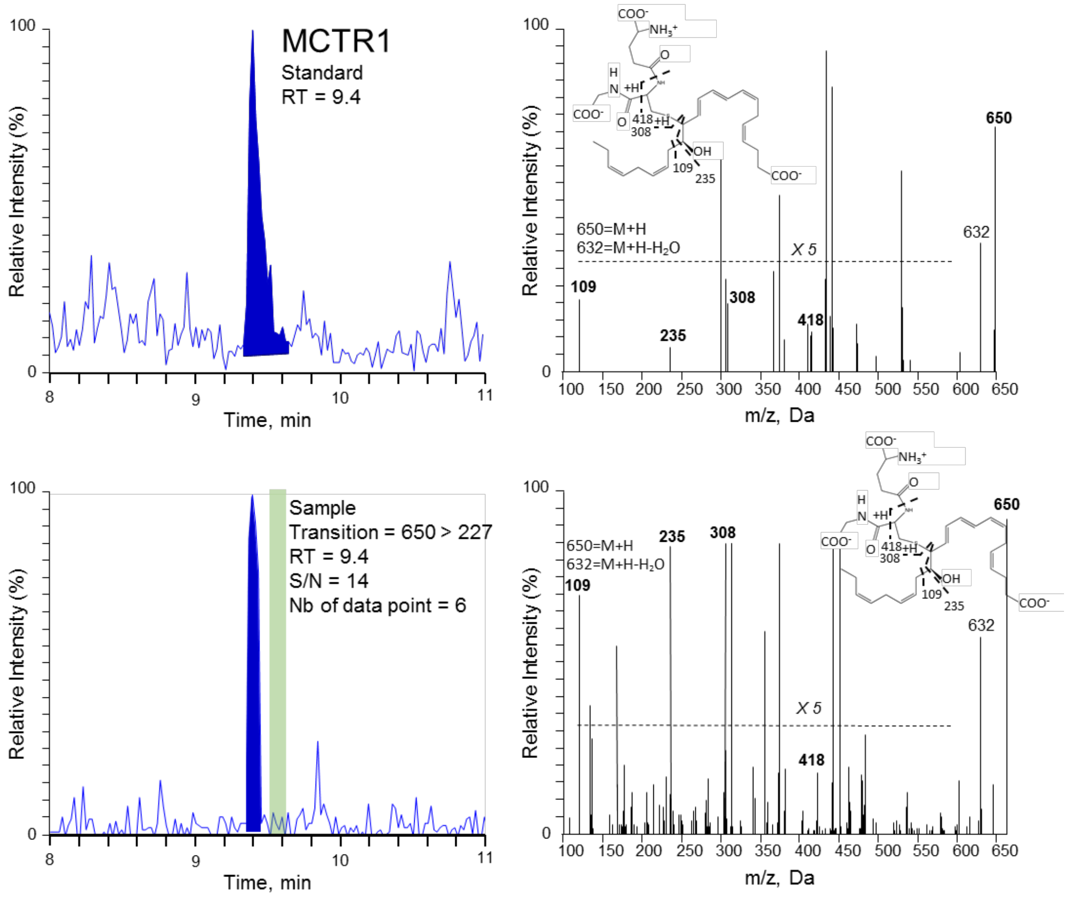
MCTR1
Standard
RT = 9.4
Relative Intensity (%)
Time, min
650=M+H
632=M+H-H_2O
X 5
m/z, Da
Sample
Transition = 650 > 227
RT = 9.4
S/N = 14
Nb of data point = 6

W

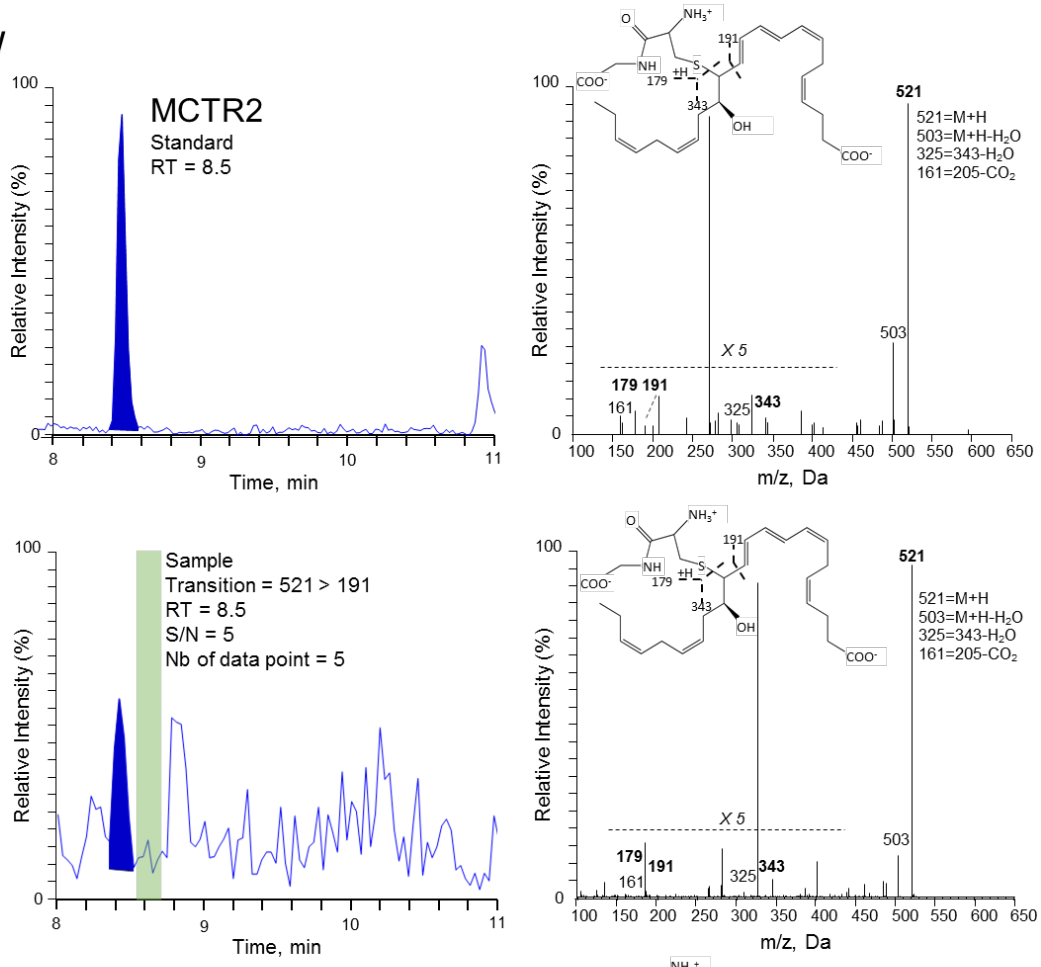
MCTR2
Standard
RT = 8.5
Sample
Transition = 521 > 191
RT = 8.5
S/N = 5
Nb of data point = 5
Relative Intensity (%)
Time, min
m/z, Da
521=M+H
503=M+H-H2O
325=343-H2O
161=205-CO2
X 5

X

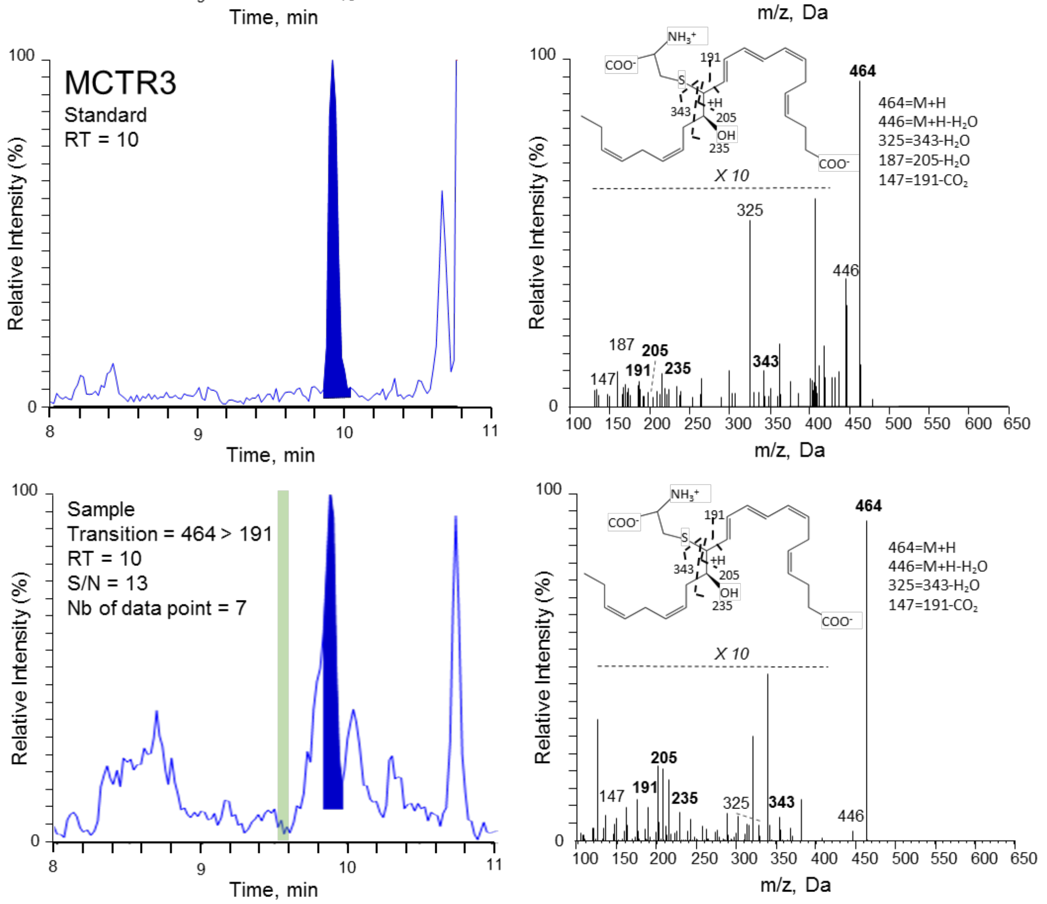
MCTR3
Standard
RT = 10
Sample
Transition = 464 > 191
RT = 10
S/N = 13
Nb of data point = 7
Relative Intensity (%)
Time, min
m/z, Da
464=M+H
446=M+H-H2O
325=343-H2O
187=205-H2O
147=191-CO2
X 10

Supplementary Figure 1: Chromatographic and MS-MS spectral evidence for identified mediators for the DHA bioactive metabolome. Representative sMRM chromatograms (*left panels)* and MS-MS fragmentation spectra (*right panels*) employed for the identification of (A-H) D-series resolvins, (I-L) Protectins, (M-O) PCTRs, (P-U) Maresins and (V-X) MCTRs for spiked standards in matrix (*top panels*) and in the samples (*bottom panels*). Area highlighted in blue in extracted ion chromatograms represents the area under the curve employed for quantitation of identified mediators. Insets in chromatograms report sMRM transition (Transition), Retention tine (RT), signal to noise (S/N) ratio and number of data points in the peak. Area highlighted in green represents the area designated as the noise region that was used to calculated the S/N ratio. Insets in MS/MS spectra report mediator structure and fragment assignments.

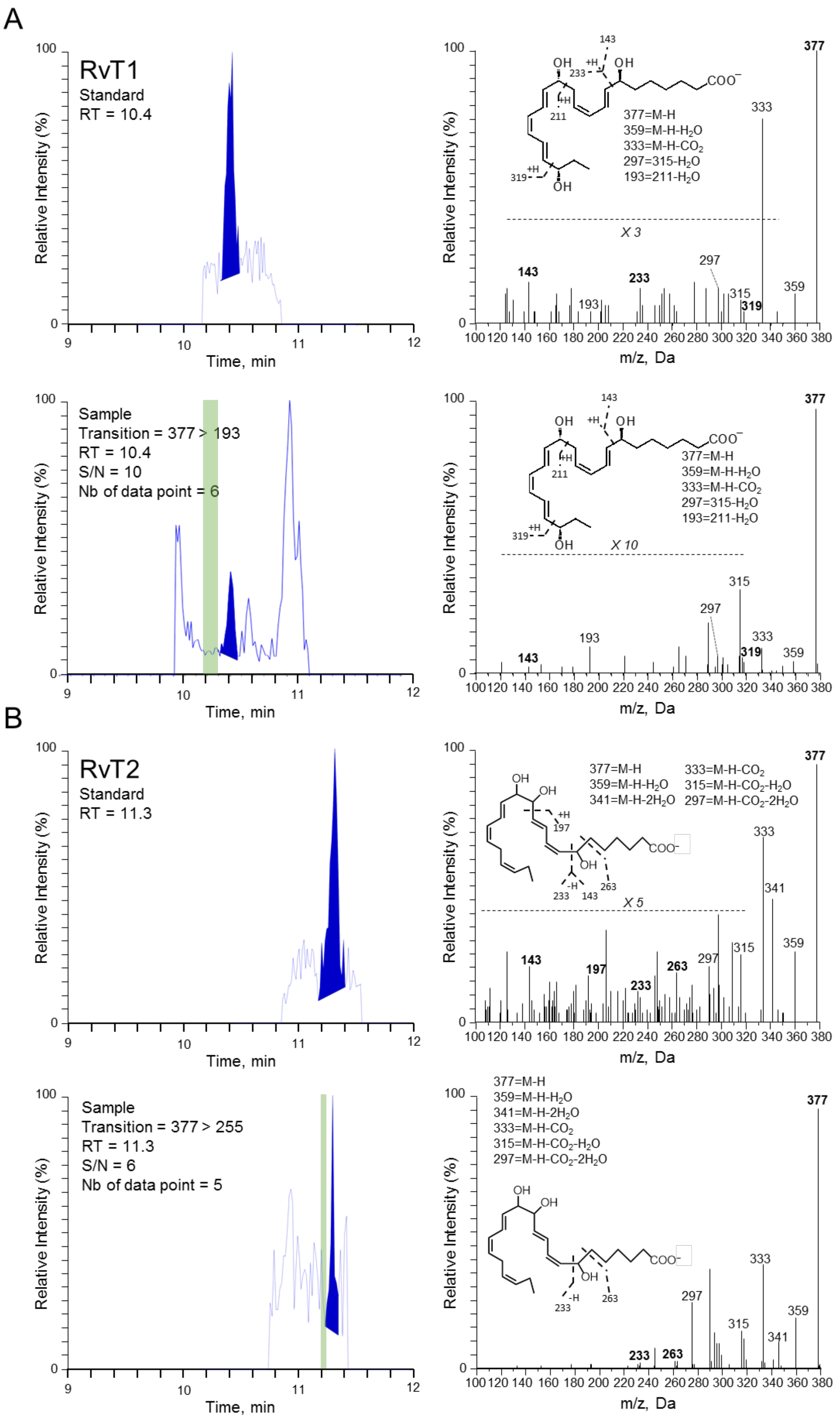
A
RvT1
Standard
RT = 10.4
Relative Intensity (%)
Time, min
377=M-H
359=M-H-H2O
333=M-H-CO2
297=315-H2O
193=211-H2O
X 3
m/z, Da
Sample
Transition = 377 > 193
RT = 10.4
S/N = 10
Nb of data point = 6
X 10
B
RvT2
Standard
RT = 11.3
377=M-H
359=M-H-H2O
341=M-H-2H2O
333=M-H-CO2
315=M-H-CO2-H2O
297=M-H-CO2-2H2O
X 5
Sample
Transition = 377 > 255
RT = 11.3
S/N = 6
Nb of data point = 5

C

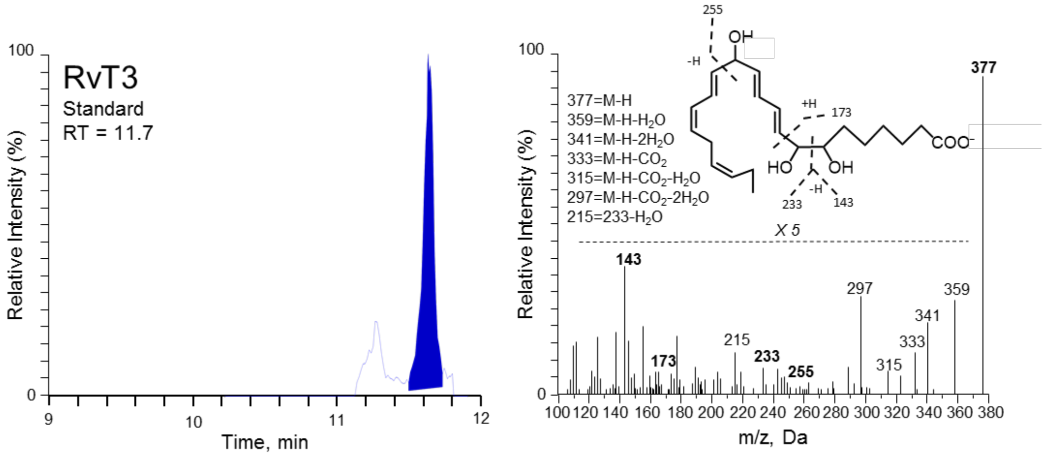
RvT3
Standard
RT = 11.7
Relative Intensity (%)
Time, min
377=M-H
359=M-H-H2O
341=M-H-2H2O
333=M-H-CO2
315=M-H-CO2-H2O
297=M-H-CO2-2H2O
215=233-H2O
X 5
m/z, Da
Sample
Transition = 377 > 197
RT = 11.7
S/N = 8
Nb of data point = 9
377=M-H
359=M-H-H2O
333=M-H-CO2
315=M-H-CO2-H2O
297=M-H-CO2-2H2O
215=233-H2O

D

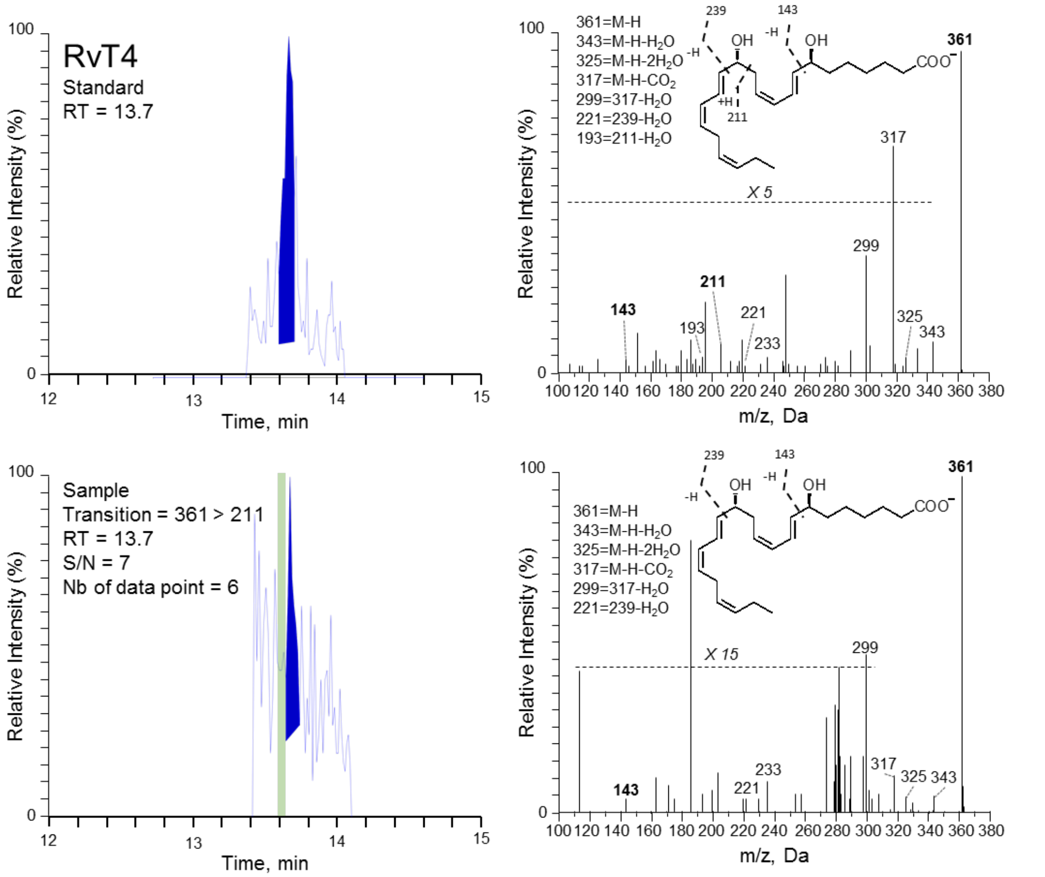
RvT4
Standard
RT = 13.7
Relative Intensity (%)
Time, min
361=M-H
343=M-H-H2O
325=M-H-2H2O
317=M-H-CO2
299=317-H2O
221=239-H2O
193=211-H2O
X 5
m/z, Da
Sample
Transition = 361 > 211
RT = 13.7
S/N = 7
Nb of data point = 6
361=M-H
343=M-H-H2O
325=M-H-2H2O
317=M-H-CO2
299=317-H2O
221=239-H2O
X 15

E

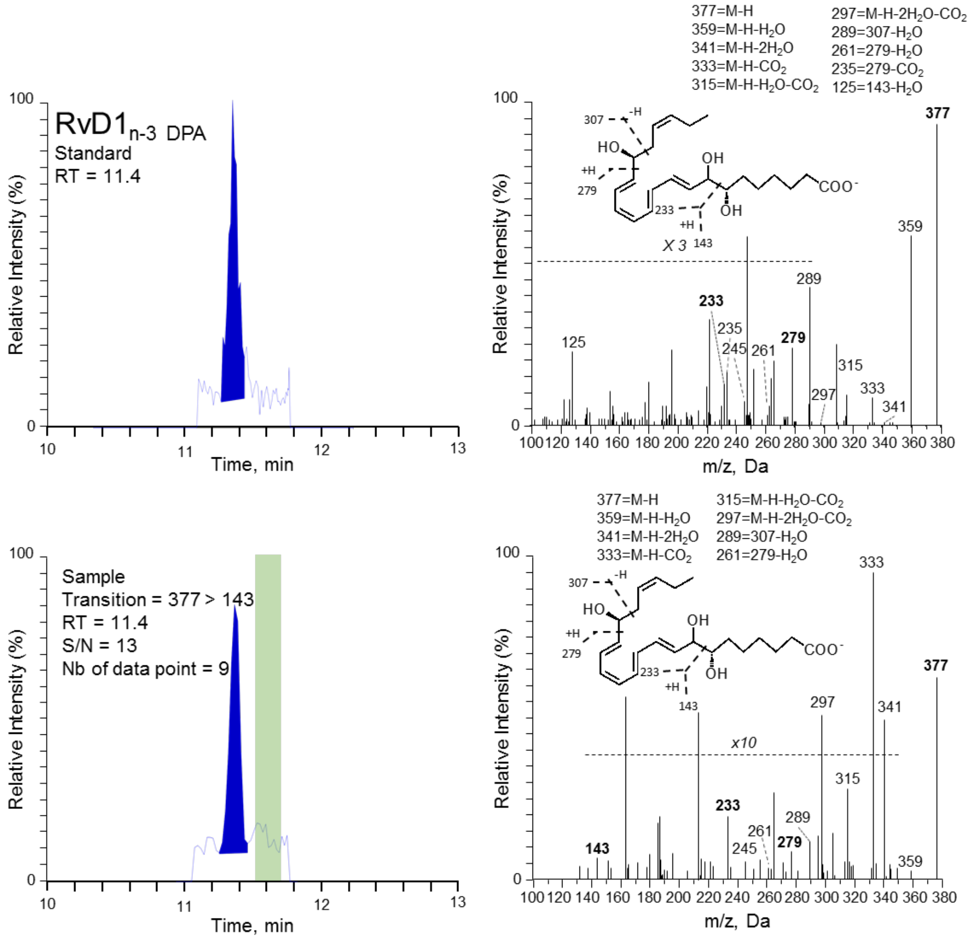

RvD1n-3 DPA
Standard
RT = 11.4
Relative Intensity (%)
Time, min
377=M-H
359=M-H-H2O
341=M-H-2H2O
333=M-H-CO2
315=M-H-H2O-CO2
297=M-H-2H2O-CO2
289=307-H2O
261=279-H2O
235=279-CO2
125=143-H2O
X 3
m/z, Da
Sample
Transition = 377 > 143
RT = 11.4
S/N = 13
Nb of data point = 9
377=M-H
359=M-H-H2O
341=M-H-2H2O
333=M-H-CO2
315=M-H-H2O-CO2
297=M-H-2H2O-CO2
289=307-H2O
261=279-H2O
x10

F

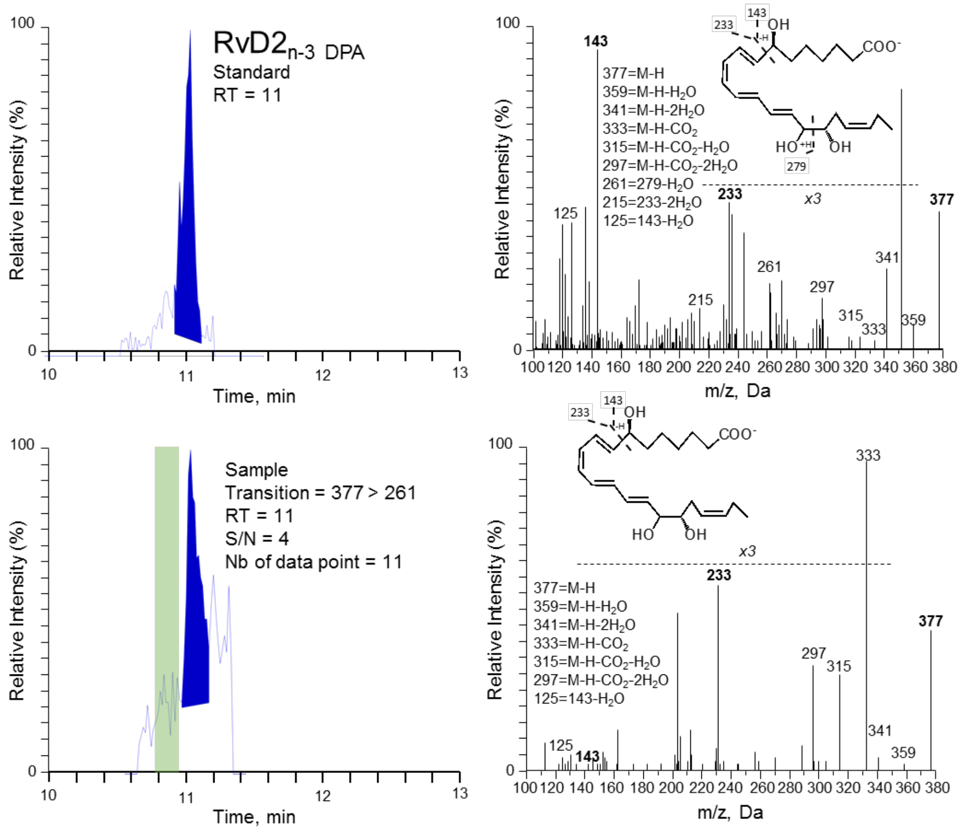

RvD2n-3 DPA
Standard
RT = 11
Relative Intensity (%)
Time, min
377=M-H
359=M-H-H2O
341=M-H-2H2O
333=M-H-CO2
315=M-H-CO2-H2O
297=M-H-CO2-2H2O
261=279-H2O
215=233-2H2O
125=143-H2O
x3
m/z, Da
Sample
Transition = 377 > 261
RT = 11
S/N = 4
Nb of data point = 11
377=M-H
359=M-H-H2O
341=M-H-2H2O
333=M-H-CO2
315=M-H-CO2-H2O
297=M-H-CO2-2H2O
125=143-H2O
x3

G

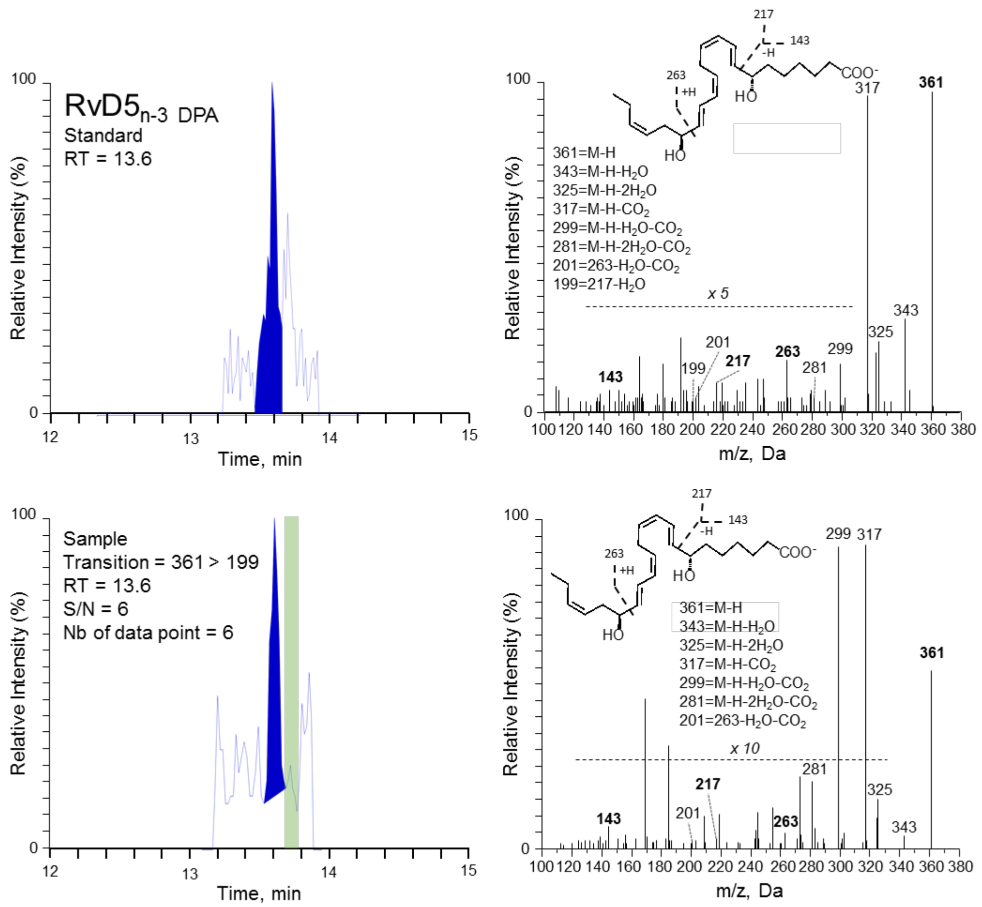

RvD5n-3 DPA
Standard
RT = 13.6
Relative Intensity (%)
Time, min
Sample
Transition = 361 > 199
RT = 13.6
S/N = 6
Nb of data point = 6
361=M-H
343=M-H-H2O
325=M-H-2H2O
317=M-H-CO2
299=M-H-H2O-CO2
281=M-H-2H2O-CO2
201=263-H2O-CO2
199=217-H2O
x 5
x 10
m/z, Da

H

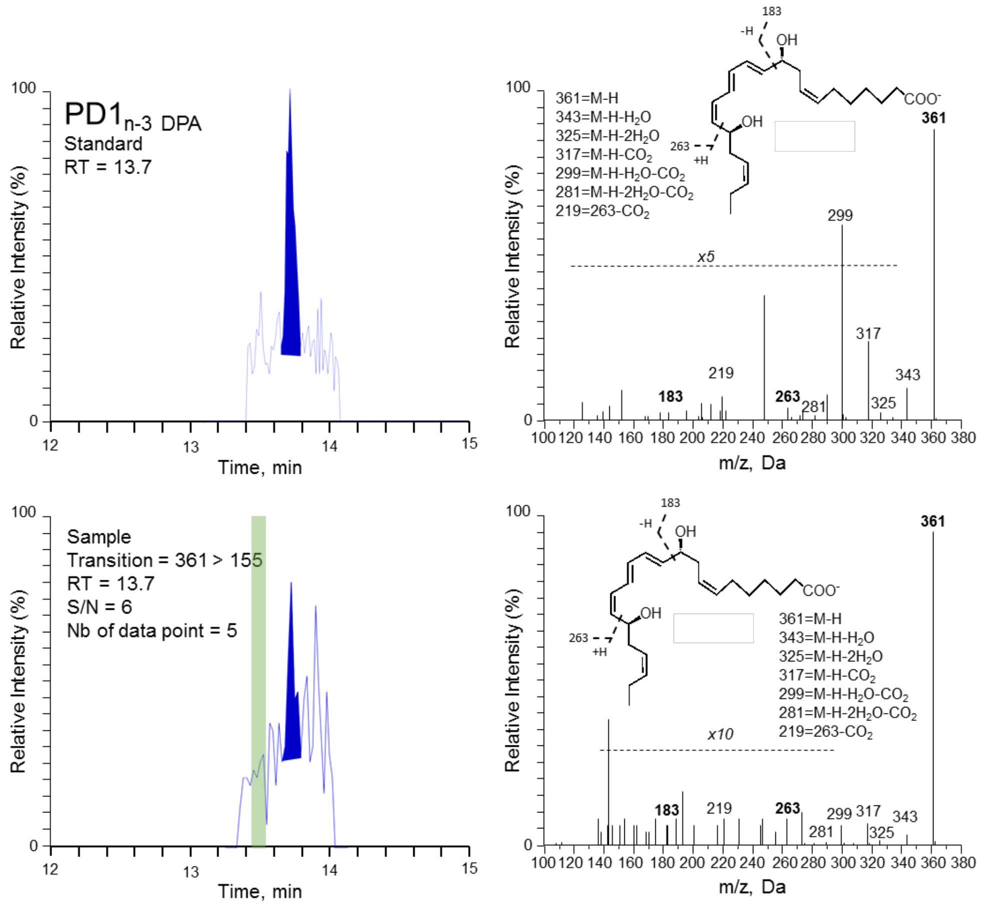

PD1n-3 DPA
Standard
RT = 13.7
Relative Intensity (%)
Time, min
Sample
Transition = 361 > 155
RT = 13.7
S/N = 6
Nb of data point = 5
361=M-H
343=M-H-H2O
325=M-H-2H2O
317=M-H-CO2
299=M-H-H2O-CO2
281=M-H-2H2O-CO2
219=263-CO2
x5
x10
m/z, Da

I

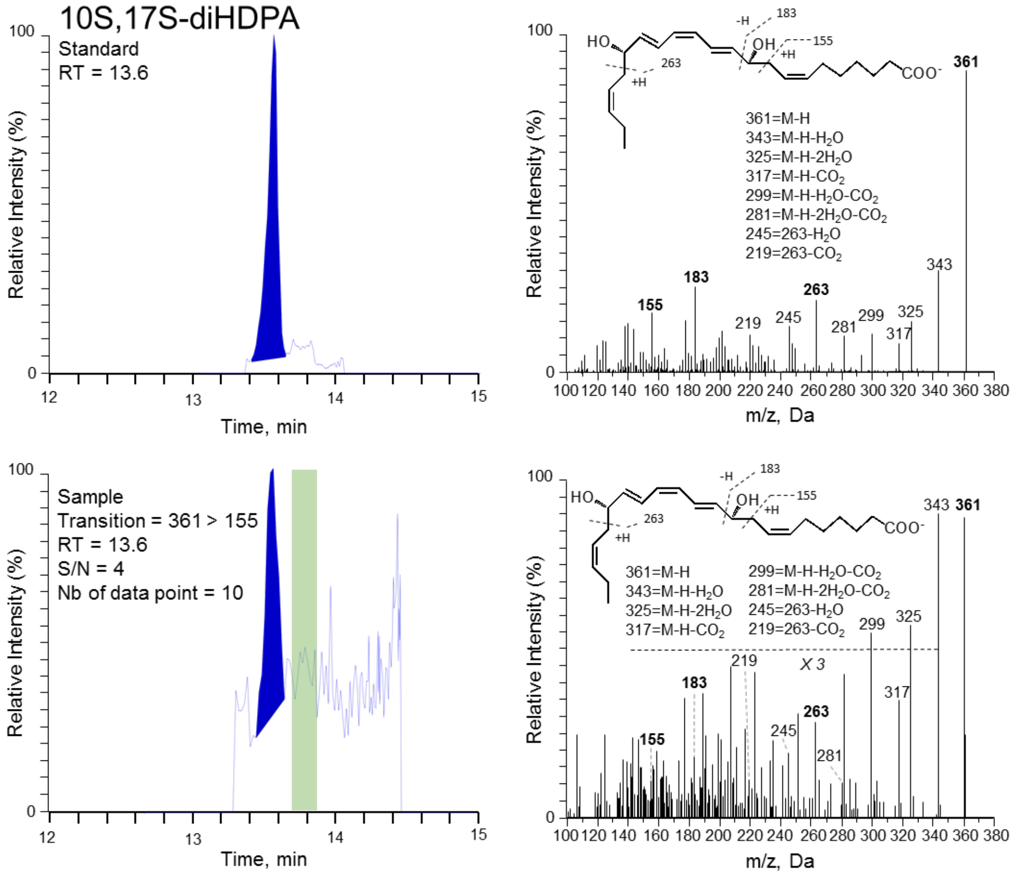

10S,17S-diHDPA
Standard
RT = 13.6
Relative Intensity (%)
Time, min
361=M-H
343=M-H-H2O
325=M-H-2H2O
317=M-H-CO2
299=M-H-H2O-CO2
281=M-H-2H2O-CO2
245=263-H2O
219=263-CO2
m/z, Da
Sample
Transition = 361 > 155
RT = 13.6
S/N = 4
Nb of data point = 10
X 3

J

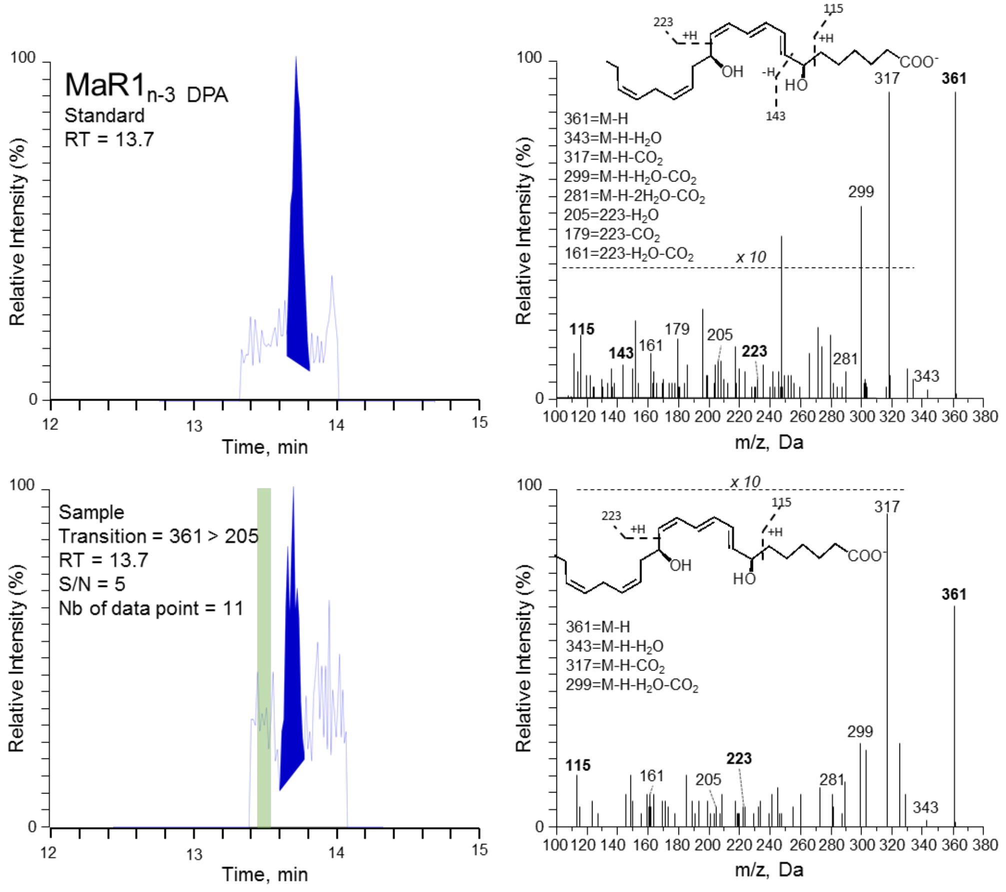

MaR1n-3 DPA
Standard
RT = 13.7
Relative Intensity (%)
Time, min
361=M-H
343=M-H-H2O
317=M-H-CO2
299=M-H-H2O-CO2
281=M-H-2H2O-CO2
205=223-H2O
179=223-CO2
161=223-H2O-CO2
x 10
m/z, Da
Sample
Transition = 361 > 205
RT = 13.7
S/N = 5
Nb of data point = 11

Supplementary Figure 2: Chromatographic and MS-MS spectral evidence for identified mediators for the n-3 DPA bioactive metabolome. Representative sMRM chromatograms (*left panels)* and MS-MS fragmentation spectra (*right panels*) employed for the identification of (A-D) 13-series resolvins, (E-G) D-series resolvins, (H-I) Protectins and (J) Maresins and for spiked standard in matrix (*top panels*) and in the samples (*bottom panels*). Area highlighted in blue in extracted ion chromatograms represents the area under the curve employed for quantitation of identified mediators. Insets in chromatograms report sMRM transition (Transition), Retention tine (RT), signal to noise (S/N) ratio and number of data points in the peak. Area highlighted in green represents the area designated as the noise region that was used to calculated the S/N ratio. Insets in MS/MS spectra report mediator structure and fragment assignments.

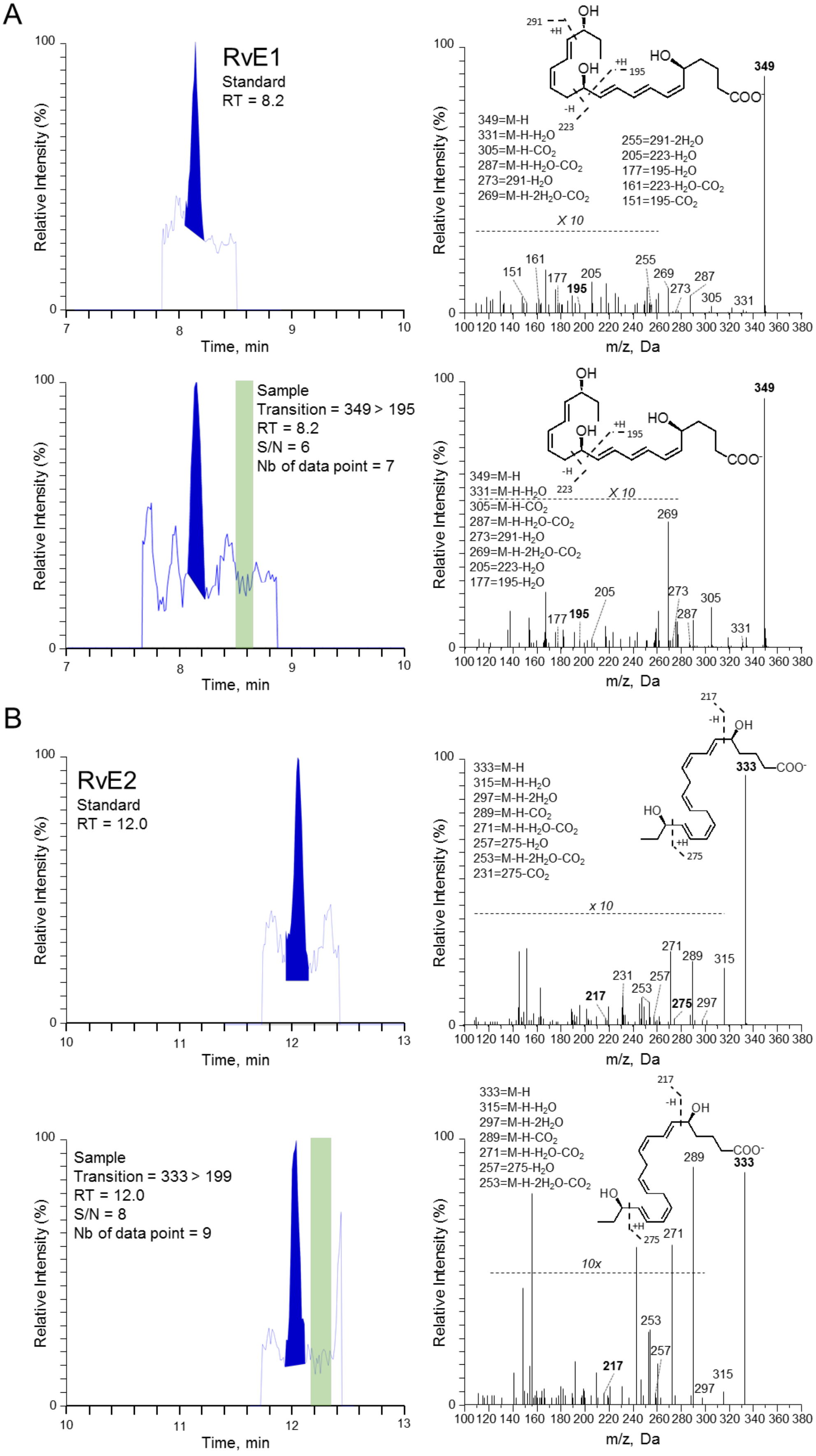
A
RvE1
Standard
RT = 8.2
Relative Intensity (%)
Time, min
349=M-H
331=M-H-H_2O
305=M-H-CO_2
287=M-H-H_2O-CO_2
273=291-H_2O
269=M-H-2H_2O-CO_2
255=291-2H_2O
205=223-H_2O
177=195-H_2O
161=223-H_2O-CO_2
151=195-CO_2
X 10
m/z, Da
Sample
Transition = 349 > 195
RT = 8.2
S/N = 6
Nb of data point = 7
349=M-H
331=M-H-H_2O
305=M-H-CO_2
287=M-H-H_2O-CO_2
273=291-H_2O
269=M-H-2H_2O-CO_2
205=223-H_2O
177=195-H_2O
B
RvE2
Standard
RT = 12.0
333=M-H
315=M-H-H_2O
297=M-H-2H_2O
289=M-H-CO_2
271=M-H-H_2O-CO_2
257=275-H_2O
253=M-H-2H_2O-CO_2
231=275-CO_2
x 10
Sample
Transition = 333 > 199
RT = 12.0
S/N = 8
Nb of data point = 9
333=M-H
315=M-H-H_2O
297=M-H-2H_2O
289=M-H-CO_2
271=M-H-H_2O-CO_2
257=275-H_2O
253=M-H-2H_2O-CO_2
10x

C

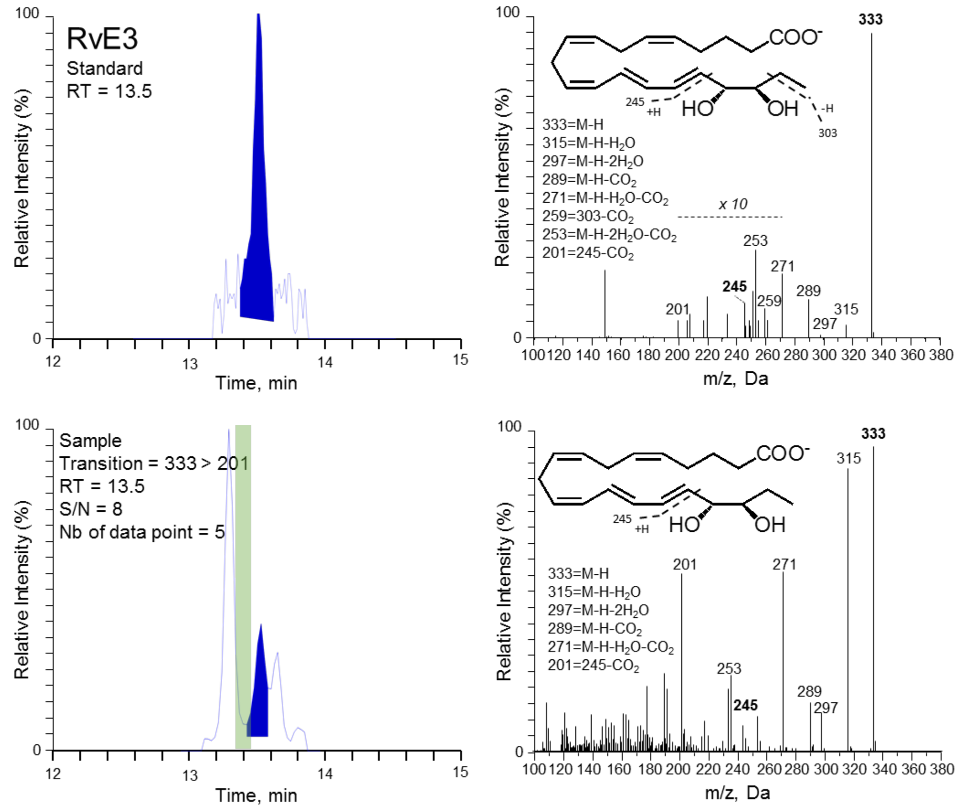

Supplementary Figure 3: Chromatographic and MS-MS spectral evidence for identified mediators for the EPA bioactive metabolome. Representative sMRM chromatograms (*left panels)* and MS-MS fragmentation spectra (*right panels*) employed for the identification of E-series resolvins for spiked standard in matrix (*top panels*) and in the samples (*bottom panels*). Area highlighted in blue in extracted ion chromatograms represents the area under the curve employed for quantitation of identified mediators. Insets in chromatograms report sMRM transition (Transition), Retention tine (RT), signal to noise (S/N) ratio and number of data points in the peak. Area highlighted in green represents the area designated as the noise region that was used to calculated the S/N ratio. Insets in MS/MS spectra report mediator structure and fragment assignments.

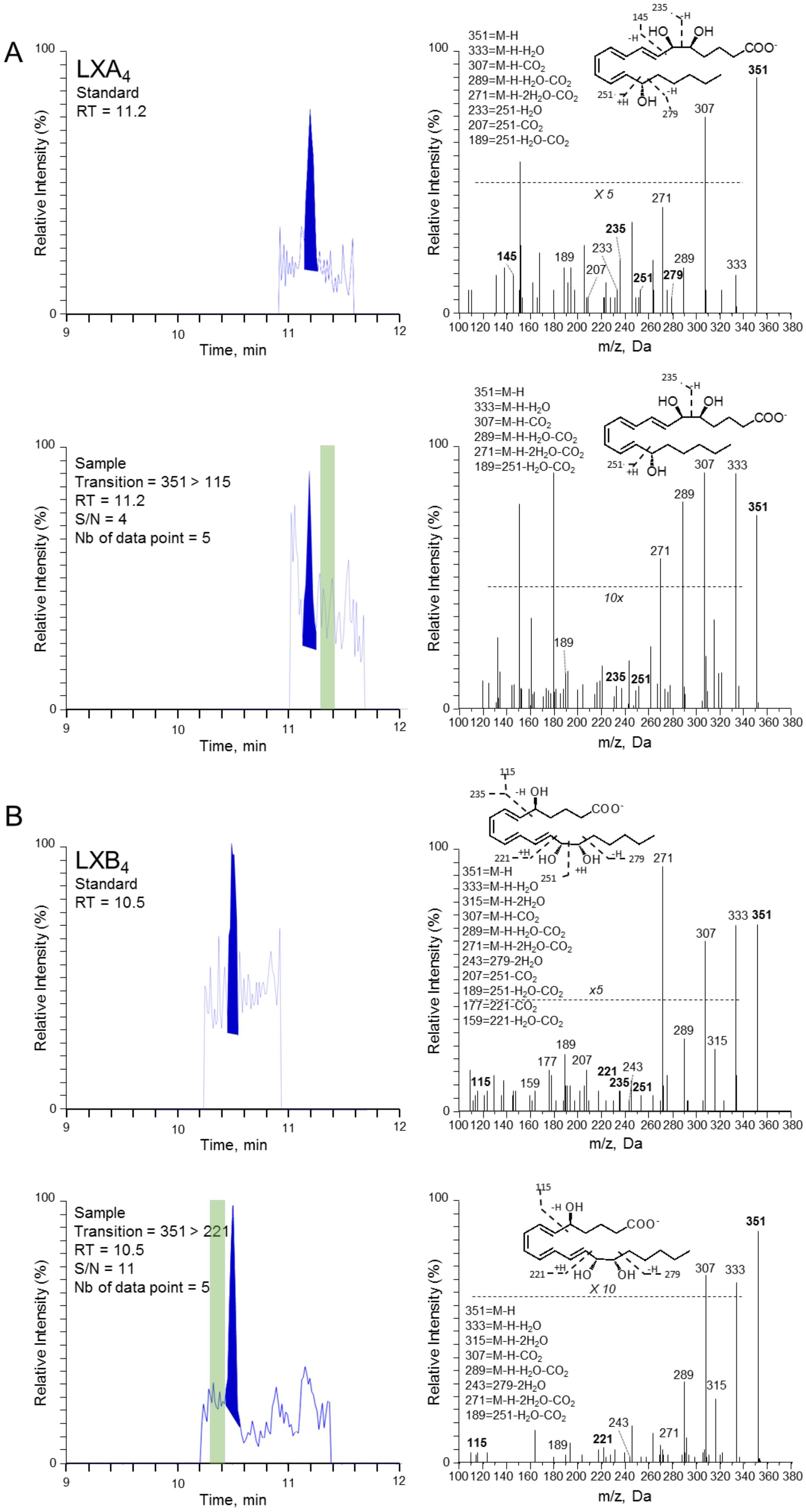
A
LXA4
Standard
RT = 11.2
Relative Intensity (%)
Time, min
351=M-H
333=M-H-H2O
307=M-H-CO2
289=M-H-H2O-CO2
271=M-H-2H2O-CO2
233=251-H2O
207=251-CO2
189=251-H2O-CO2
X 5
m/z, Da
Sample
Transition = 351 > 115
RT = 11.2
S/N = 4
Nb of data point = 5
351=M-H
333=M-H-H2O
307=M-H-CO2
289=M-H-H2O-CO2
271=M-H-2H2O-CO2
189=251-H2O-CO2
10x
B
LXB4
Standard
RT = 10.5
351=M-H
333=M-H-H2O
315=M-H-2H2O
307=M-H-CO2
289=M-H-H2O-CO2
271=M-H-2H2O-CO2
243=279-2H2O
207=251-CO2
189=251-H2O-CO2
177=221-CO2
159=221-H2O-CO2
x5
Sample
Transition = 351 > 221
RT = 10.5
S/N = 11
Nb of data point = 5
X 10
351=M-H
333=M-H-H2O
315=M-H-2H2O
307=M-H-CO2
289=M-H-H2O-CO2
243=279-2H2O
271=M-H-2H2O-CO2
189=251-H2O-CO2

C

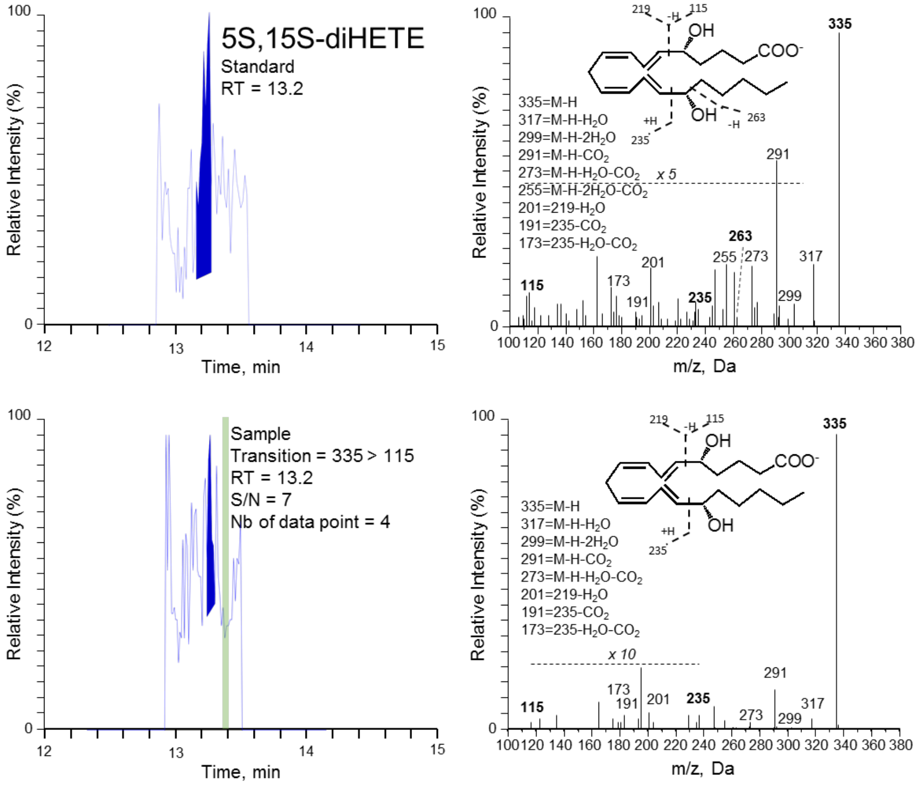

5S,15S-diHETE
Standard
RT = 13.2
Relative Intensity (%)
Time, min
335=M-H
317=M-H-H2O
299=M-H-2H2O
291=M-H-CO2
273=M-H-H2O-CO2
255=M-H-2H2O-CO2
201=219-H2O
191=235-CO2
173=235-H2O-CO2
x 5
m/z, Da
Sample
Transition = 335 > 115
RT = 13.2
S/N = 7
Nb of data point = 4
x 10

D

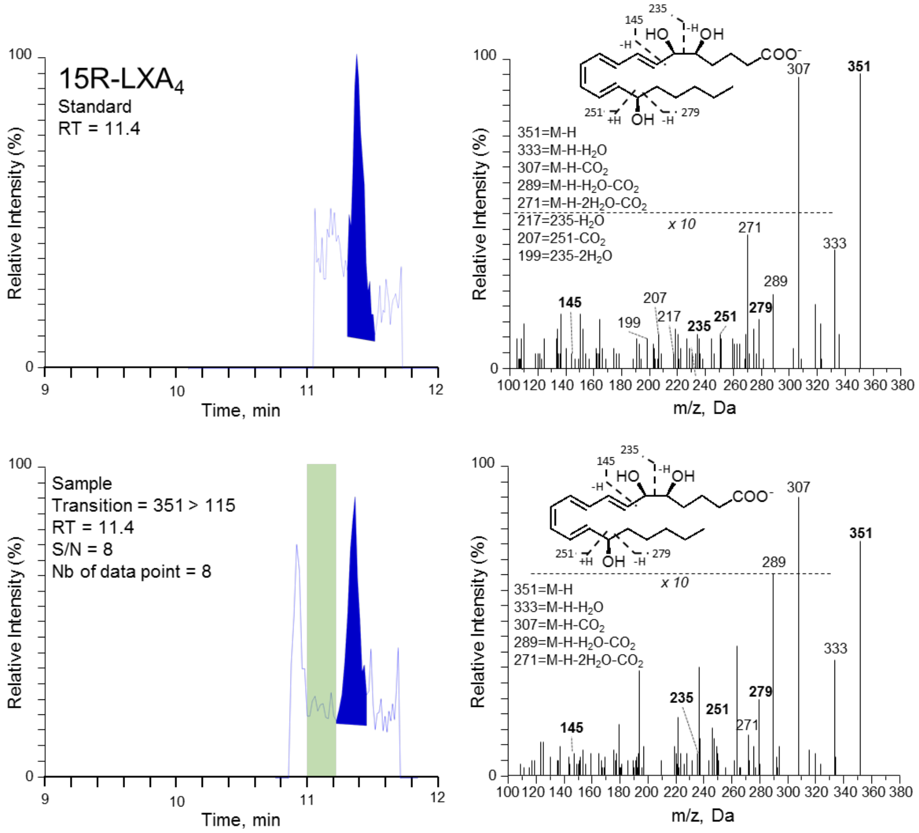

15R-LXA4
Standard
RT = 11.4
Relative Intensity (%)
Time, min
351=M-H
333=M-H-H2O
307=M-H-CO2
289=M-H-H2O-CO2
271=M-H-2H2O-CO2
217=235-H2O
207=251-CO2
199=235-2H2O
x 10
m/z, Da
Sample
Transition = 351 > 115
RT = 11.4
S/N = 8
Nb of data point = 8

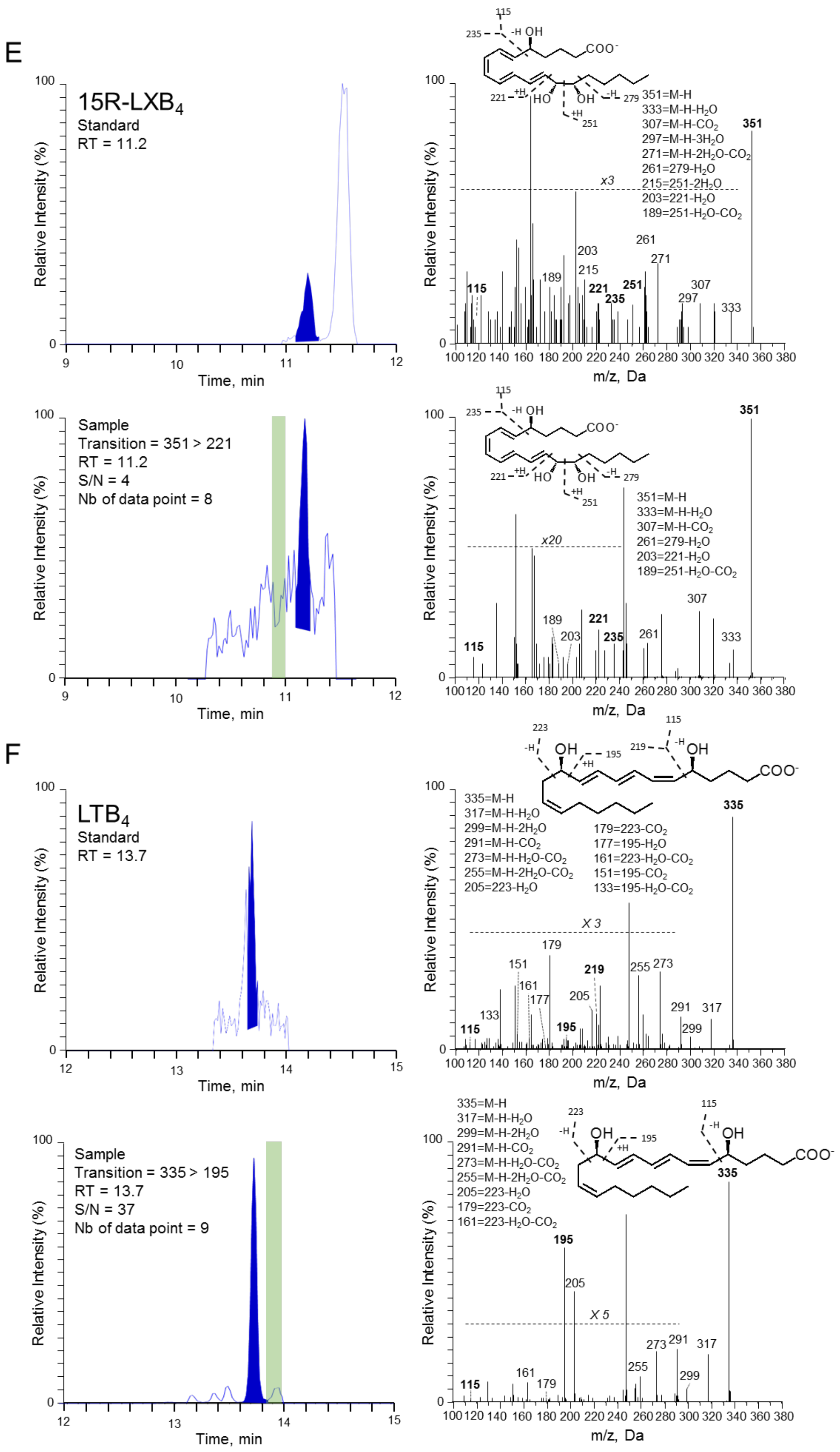
E
15R-LXB4
Standard
RT = 11.2
Sample
Transition = 351 > 221
RT = 11.2
S/N = 4
Nb of data point = 8
351=M-H
333=M-H-H2O
307=M-H-CO2
297=M-H-3H2O
271=M-H-2H2O-CO2
261=279-H2O
215=251-2H2O
203=221-H2O
189=251-H2O-CO2
F
LTB4
Standard
RT = 13.7
Sample
Transition = 335 > 195
RT = 13.7
S/N = 37
Nb of data point = 9
335=M-H
317=M-H-H2O
299=M-H-2H2O
291=M-H-CO2
273=M-H-H2O-CO2
255=M-H-2H2O-CO2
205=223-H2O
179=223-CO2
177=195-H2O
161=223-H2O-CO2
151=195-CO2
133=195-H2O-CO2
Relative Intensity (%)
Time, min
m/z, Da

G

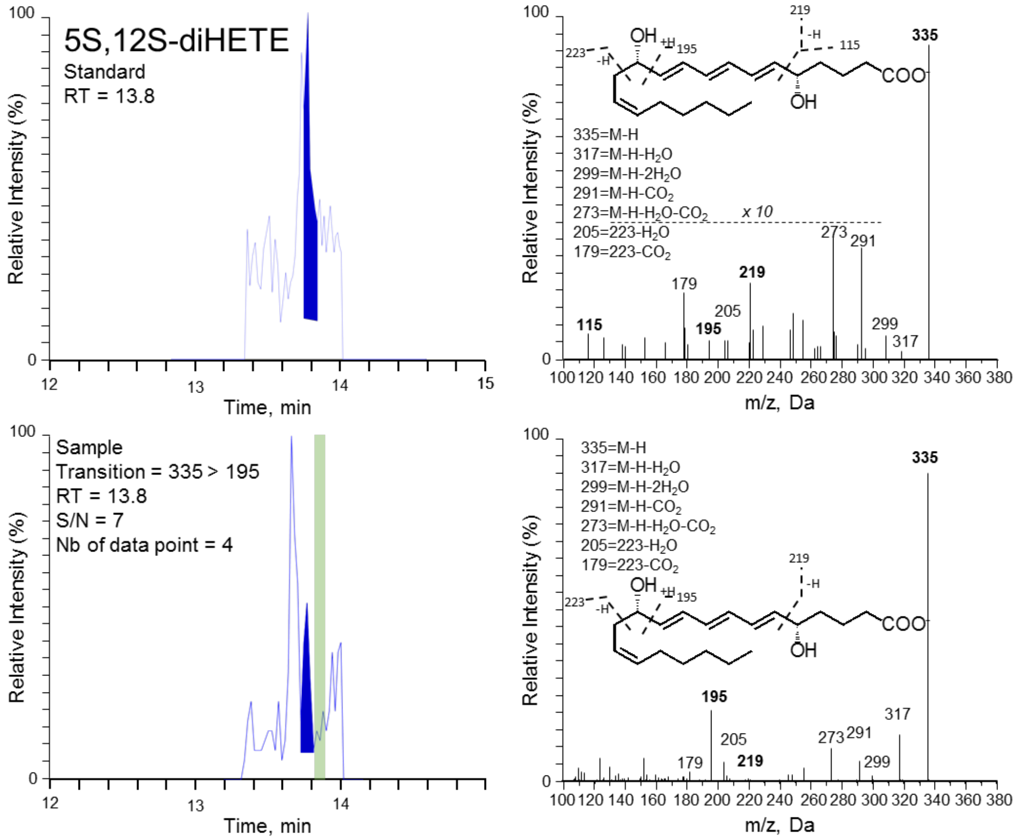

5S,12S-diHETE
Standard
RT = 13.8
Relative Intensity (%)
Time, min
335=M-H
317=M-H-H2O
299=M-H-2H2O
291=M-H-CO2
273=M-H-H2O-CO2
205=223-H2O
179=223-CO2
x 10
m/z, Da
Sample
Transition = 335 > 195
RT = 13.8
S/N = 7
Nb of data point = 4

H

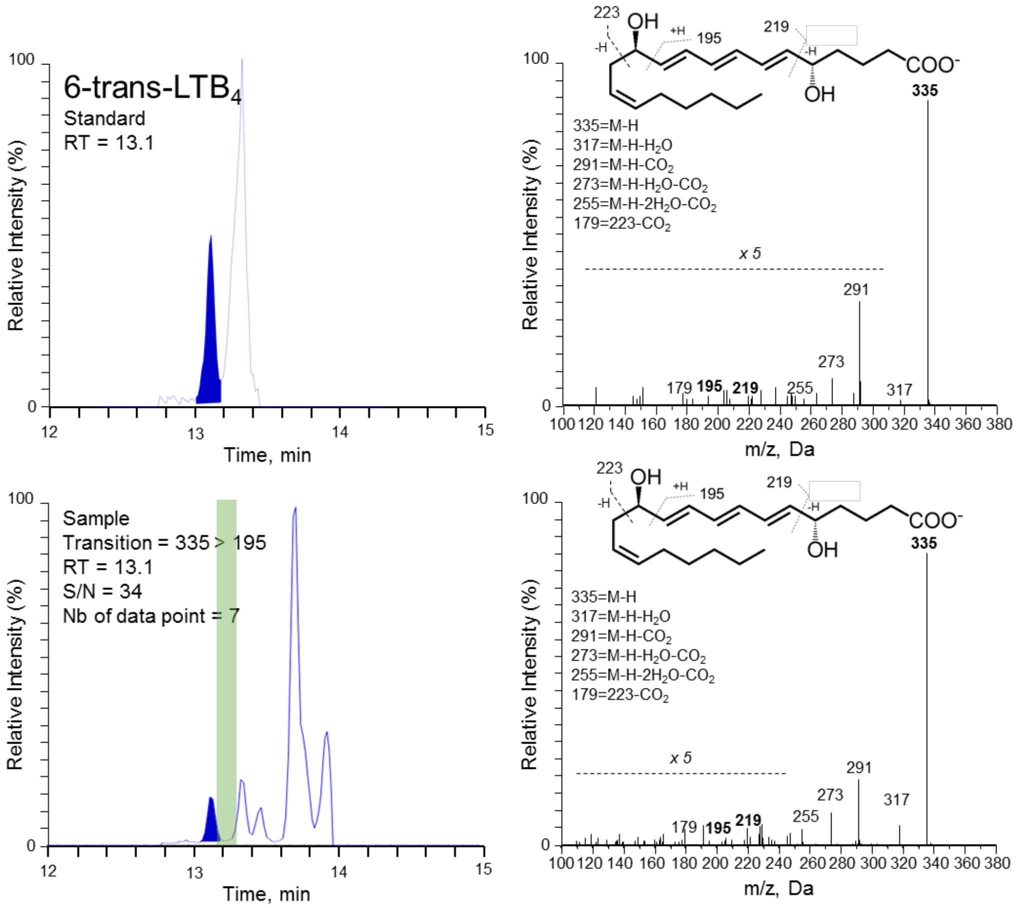

6-trans-LTB4
Standard
RT = 13.1
Relative Intensity (%)
Time, min
335=M-H
317=M-H-H2O
291=M-H-CO2
273=M-H-H2O-CO2
255=M-H-2H2O-CO2
179=223-CO2
x 5
m/z, Da
Sample
Transition = 335 > 195
RT = 13.1
S/N = 34
Nb of data point = 7

I

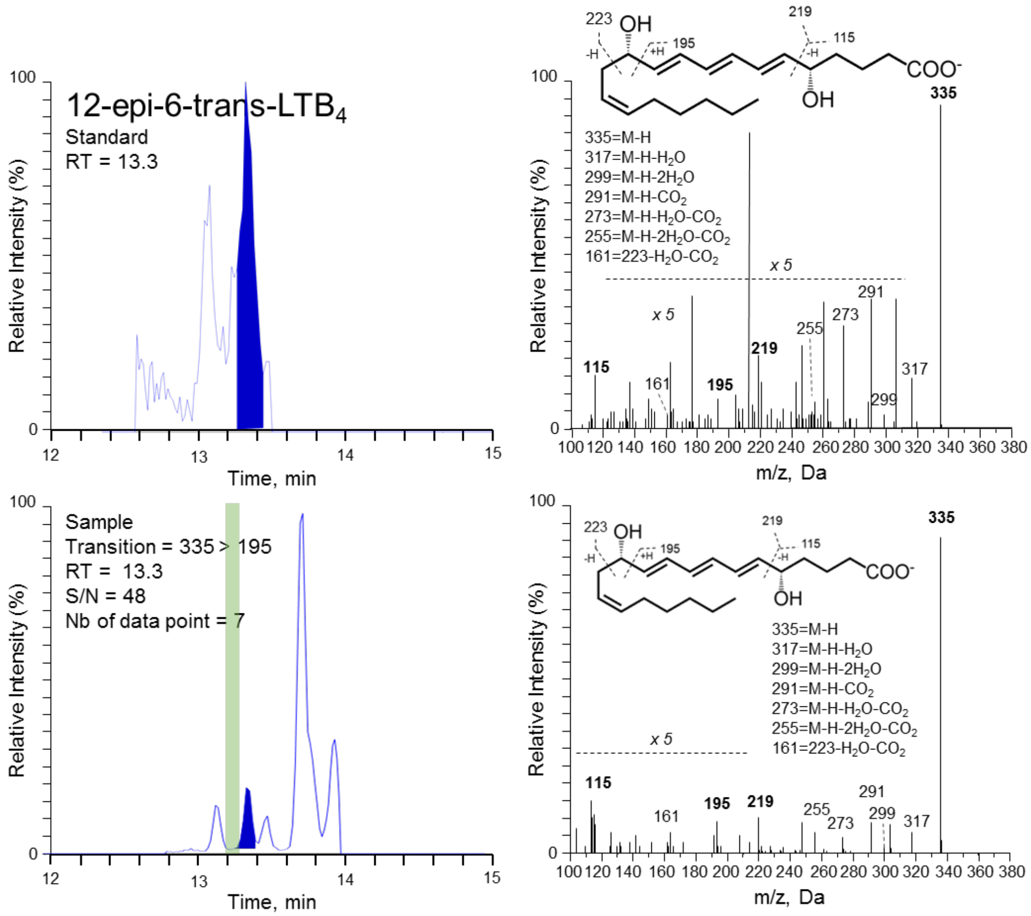
12-epi-6-trans-LTB4
Standard
RT = 13.3
Relative Intensity (%)
Time, min
335=M-H
317=M-H-H2O
299=M-H-2H2O
291=M-H-CO2
273=M-H-H2O-CO2
255=M-H-2H2O-CO2
161=223-H2O-CO2
x 5
m/z, Da
Sample
Transition = 335 > 195
RT = 13.3
S/N = 48
Nb of data point = 7

J

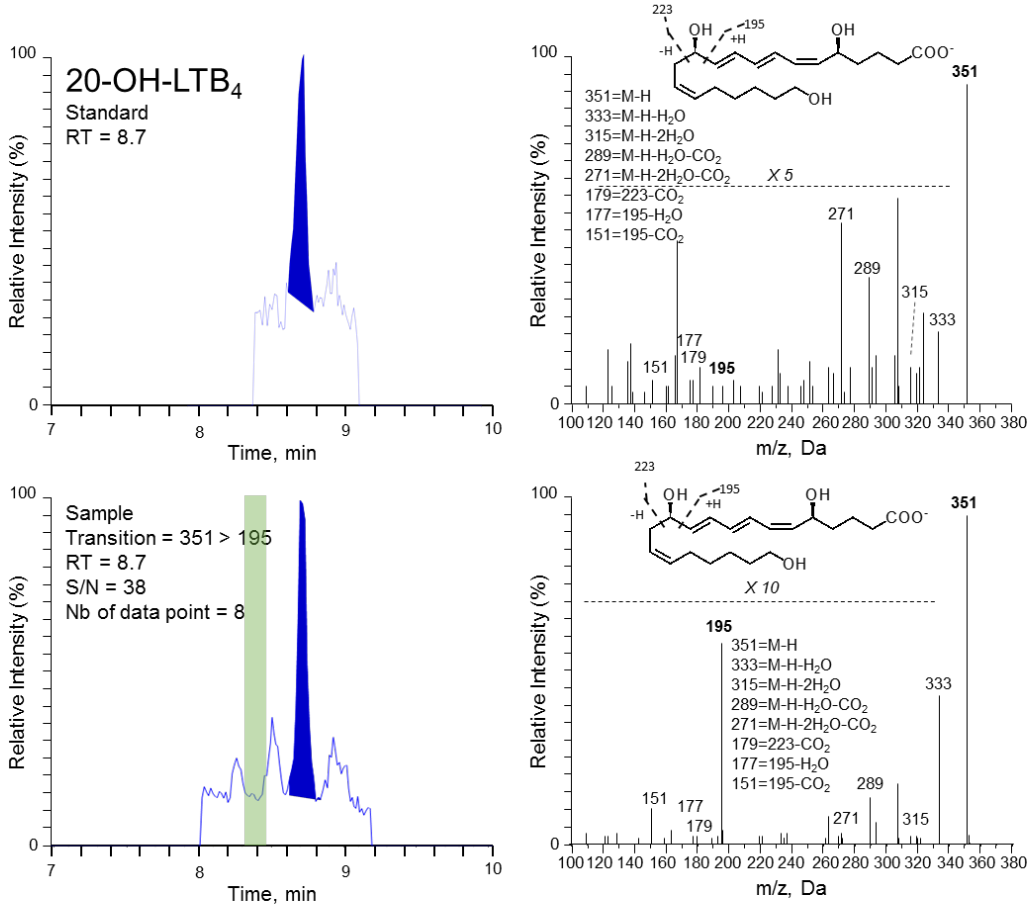
20-OH-LTB4
Standard
RT = 8.7
Relative Intensity (%)
Time, min
351=M-H
333=M-H-H2O
315=M-H-2H2O
289=M-H-H2O-CO2
271=M-H-2H2O-CO2
179=223-CO2
177=195-H2O
151=195-CO2
X 5
m/z, Da
Sample
Transition = 351 > 195
RT = 8.7
S/N = 38
Nb of data point = 8
X 10

K

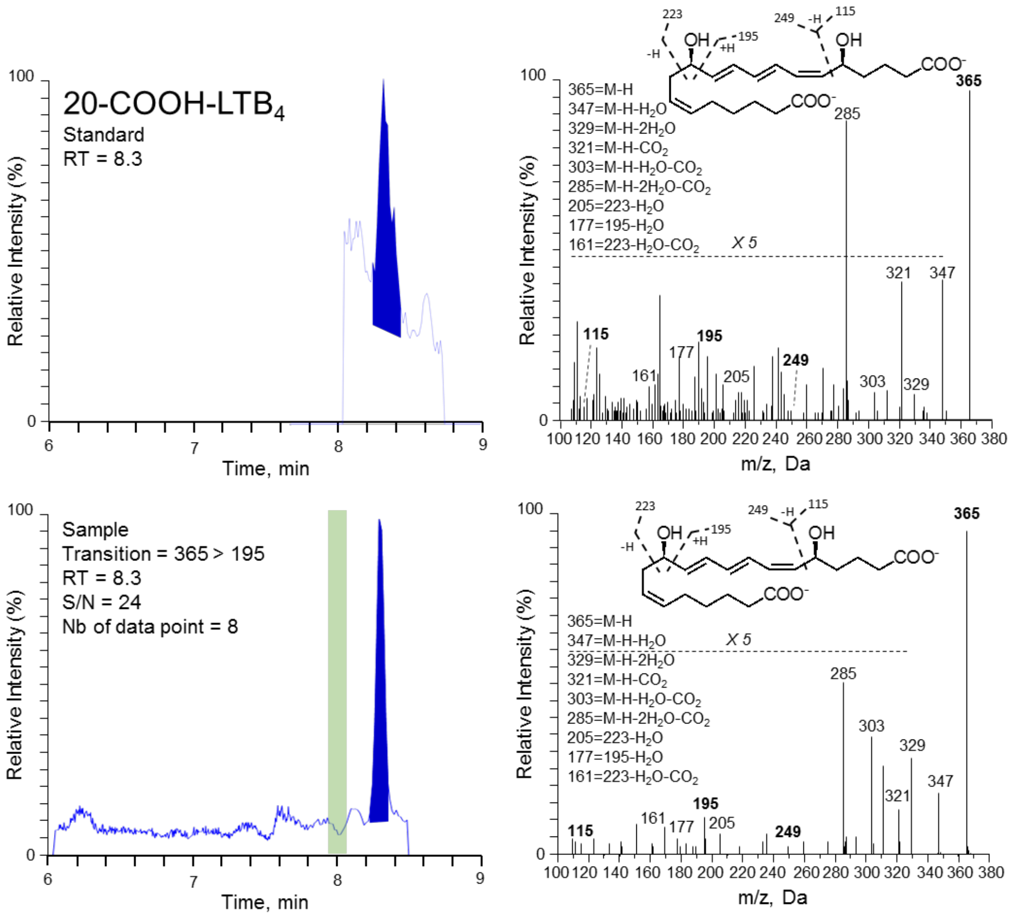
20-COOH-LTB4
Standard
RT = 8.3
Relative Intensity (%)
Time, min
365=M-H
347=M-H-H2O
329=M-H-2H2O
321=M-H-CO2
303=M-H-H2O-CO2
285=M-H-2H2O-CO2
205=223-H2O
177=195-H2O
161=223-H2O-CO2
X 5
m/z, Da
Sample
Transition = 365 > 195
RT = 8.3
S/N = 24
Nb of data point = 8

L

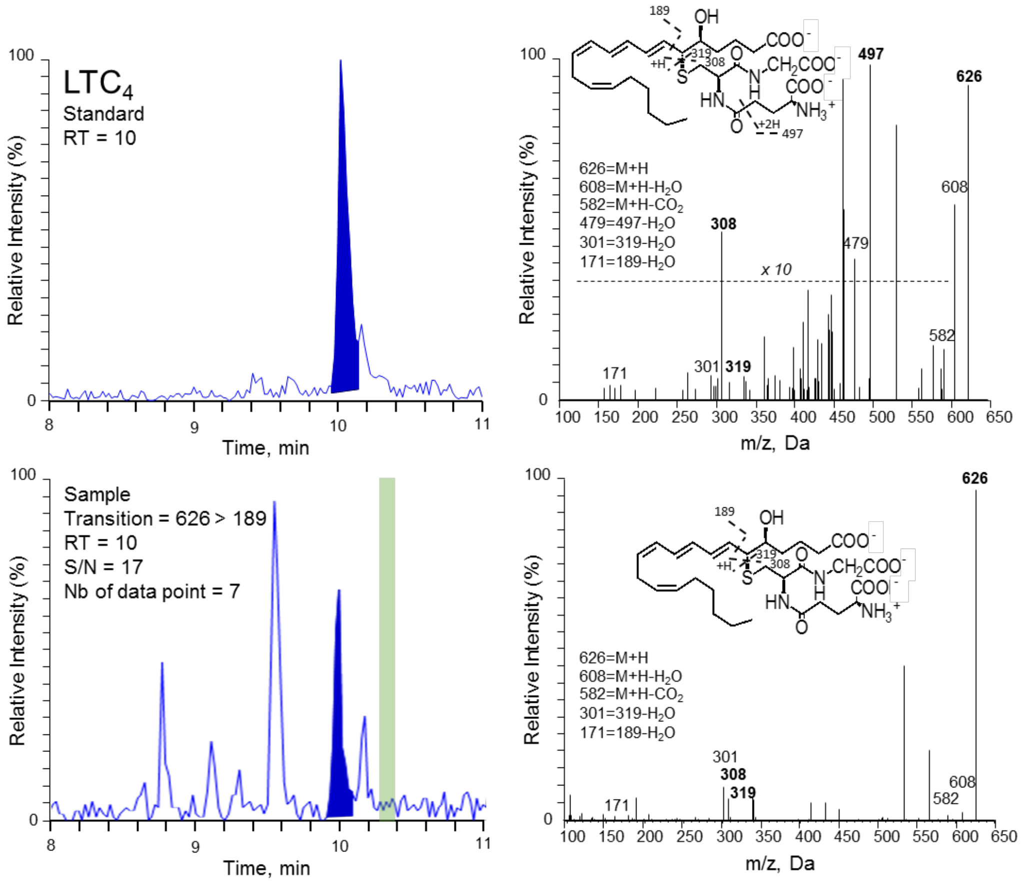
LTC4
Standard
RT = 10
Relative Intensity (%)
Time, min
626=M+H
608=M+H-H2O
582=M+H-CO2
479=497-H2O
301=319-H2O
171=189-H2O
x 10
m/z, Da
Sample
Transition = 626 > 189
RT = 10
S/N = 17
Nb of data point = 7
626=M+H
608=M+H-H2O
582=M+H-CO2
301=319-H2O
171=189-H2O

M

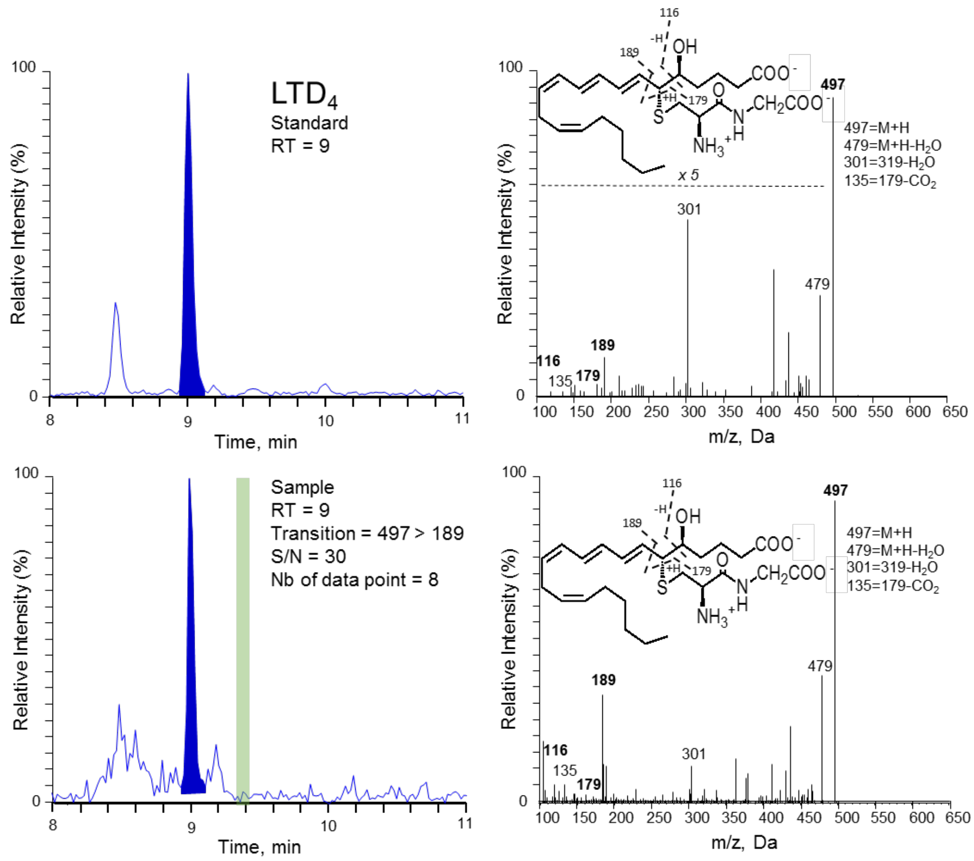
LTD4
Standard
RT = 9
Relative Intensity (%)
Time, min
497=M+H
479=M+H-H2O
301=319-H2O
135=179-CO2
x 5
m/z, Da
Sample
RT = 9
Transition = 497 > 189
S/N = 30
Nb of data point = 8

N

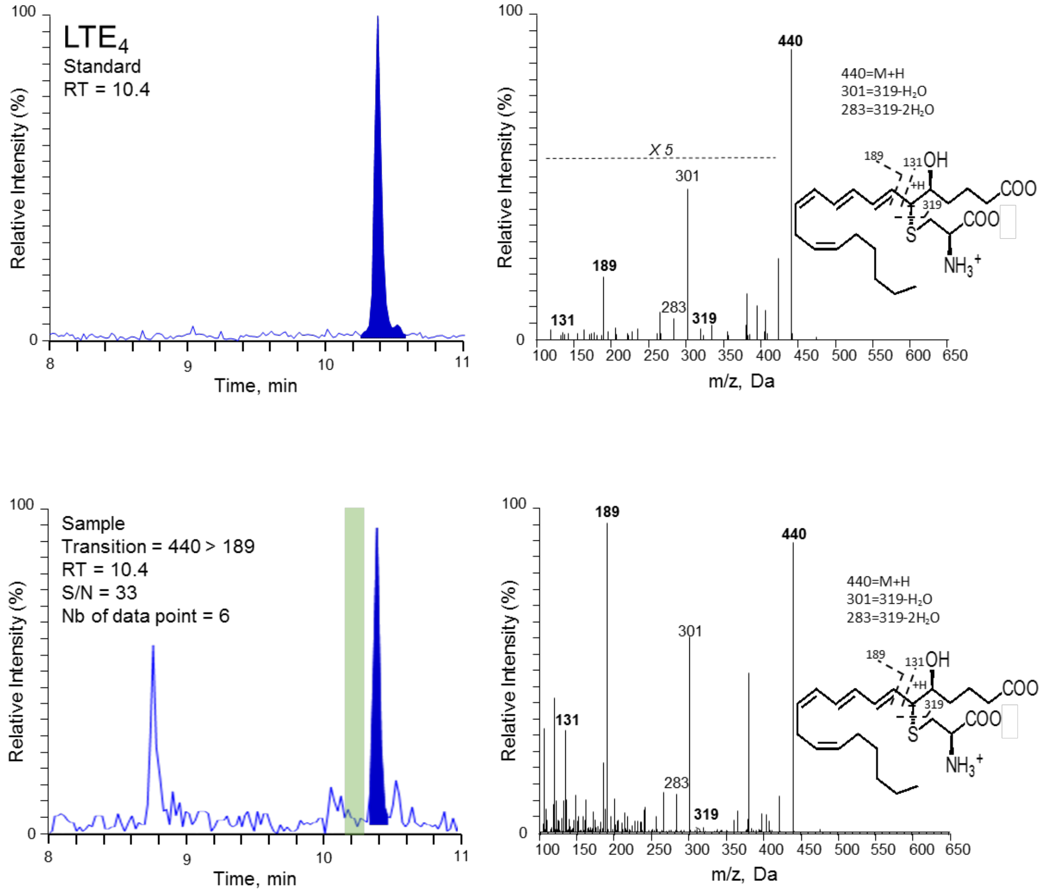
LTE4
Standard
RT = 10.4
Relative Intensity (%)
Time, min
440=M+H
301=319-H2O
283=319-2H2O
X 5
m/z, Da
Sample
Transition = 440 > 189
RT = 10.4
S/N = 33
Nb of data point = 6

O

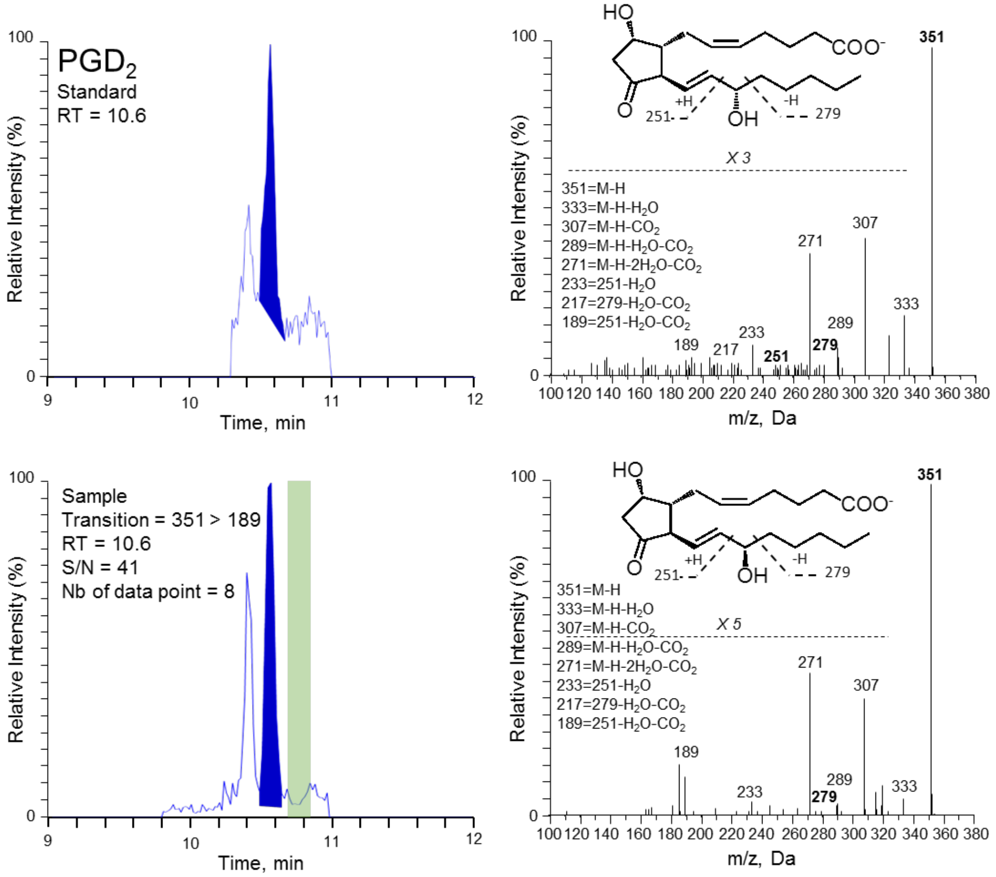
PGD2
Standard
RT = 10.6
Sample
Transition = 351 > 189
RT = 10.6
S/N = 41
Nb of data point = 8
Relative Intensity (%)
Time, min
m/z, Da
HO
COO-
O
OH
+H
-H
251
279
X 3
X 5
351=M-H
333=M-H-H2O
307=M-H-CO2
289=M-H-H2O-CO2
271=M-H-2H2O-CO2
233=251-H2O
217=279-H2O-CO2
189=251-H2O-CO2
351
307
271
333
289
279
251
233
217
189

P

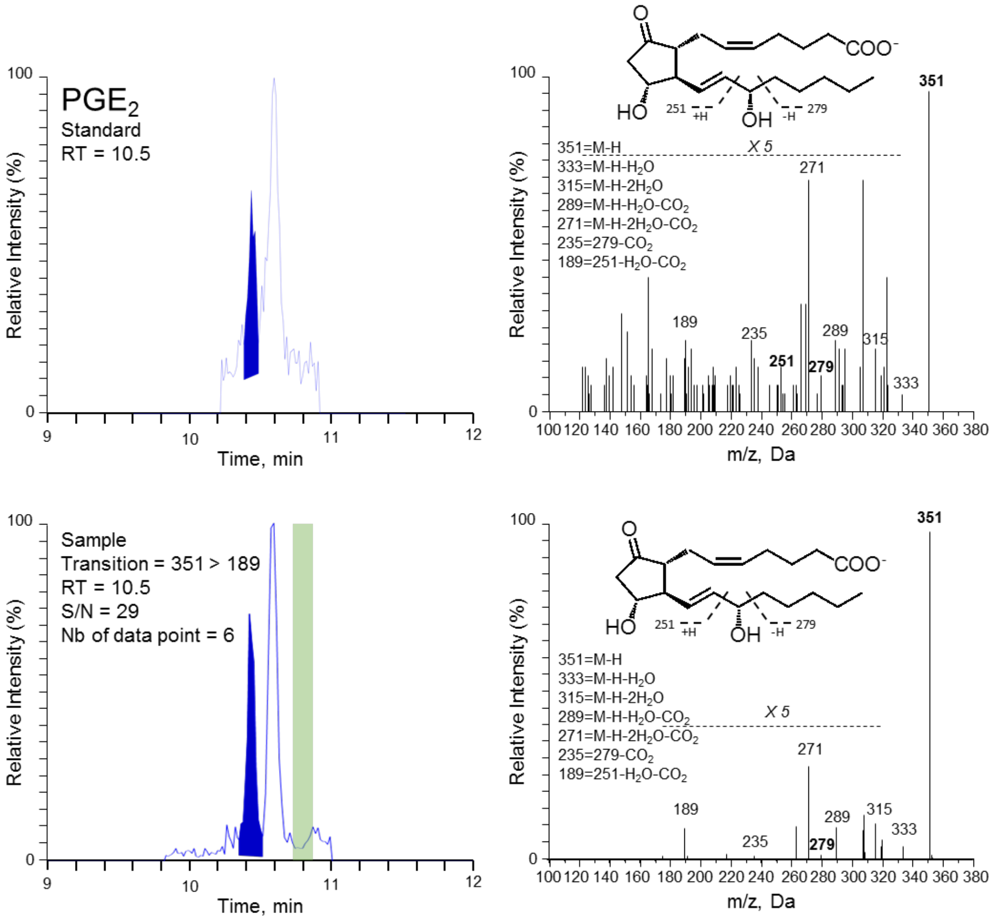
PGE2
Standard
RT = 10.5
Sample
Transition = 351 > 189
RT = 10.5
S/N = 29
Nb of data point = 6
Relative Intensity (%)
Time, min
m/z, Da
O
COO-
HO
OH
+H
-H
251
279
X 5
351=M-H
333=M-H-H2O
315=M-H-2H2O
289=M-H-H2O-CO2
271=M-H-2H2O-CO2
235=279-CO2
189=251-H2O-CO2
351
271
189
235
289
315
251
279
333

Q

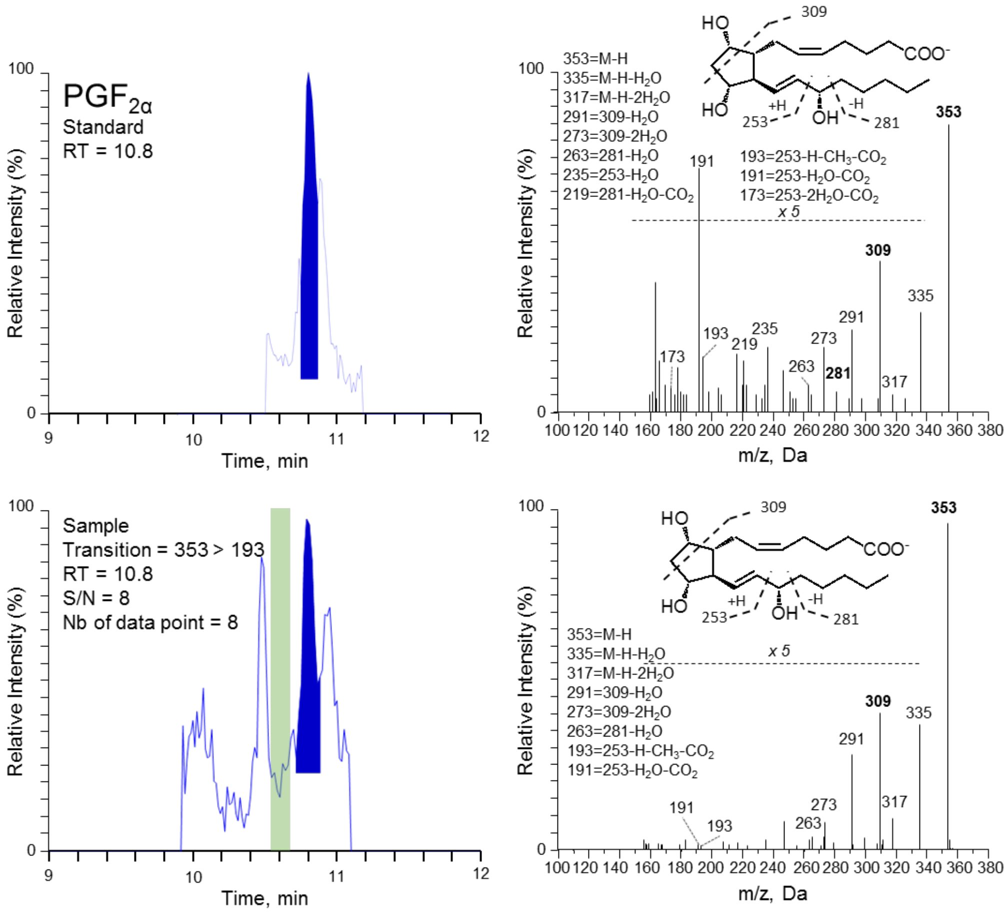

PGF2α
Standard
RT = 10.8
Relative Intensity (%)
Time, min
353=M-H
335=M-H-H2O
317=M-H-2H2O
291=309-H2O
273=309-2H2O
263=281-H2O
235=253-H2O
219=281-H2O-CO2
193=253-H-CH3-CO2
191=253-H2O-CO2
173=253-2H2O-CO2
x 5
m/z, Da
Sample
Transition = 353 > 193
RT = 10.8
S/N = 8
Nb of data point = 8
353=M-H
335=M-H-H2O
317=M-H-2H2O
291=309-H2O
273=309-2H2O
263=281-H2O
193=253-H-CH3-CO2
191=253-H2O-CO2
x 5

R

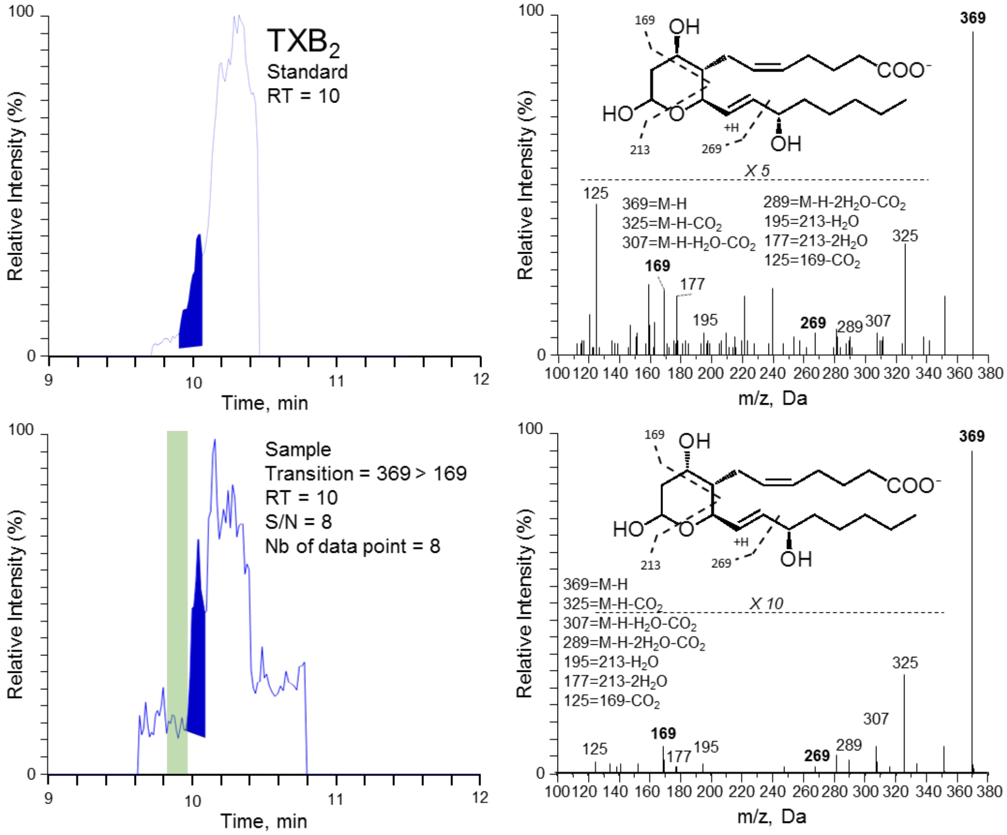

TXB2
Standard
RT = 10
Relative Intensity (%)
Time, min
369=M-H
325=M-H-CO2
307=M-H-H2O-CO2
289=M-H-2H2O-CO2
195=213-H2O
177=213-2H2O
125=169-CO2
X 5
m/z, Da
Sample
Transition = 369 > 169
RT = 10
S/N = 8
Nb of data point = 8
369=M-H
325=M-H-CO2
307=M-H-H2O-CO2
289=M-H-2H2O-CO2
195=213-H2O
177=213-2H2O
125=169-CO2
X 10

Supplementary Figure 4: Chromatographic and MS-MS spectral evidence for identified mediators for the AA bioactive metabolome. Representative sMRM chromatograms (*left panels)* and MS-MS fragmentation spectra (*right panels*) employed for the identification of (A-E) Lipoxins, (F-K) LTB_4 metabolome, (L-N) cysteinyl leukotrienes, and (O-R) Prostanoids for spiked standard in matrix (*top panels*) and in the samples (*bottom panels*). Area highlighted in blue in extracted ion chromatograms represents the area under the curve employed for quantitation of identified mediators. Insets in chromatograms report sMRM transition (Transition), Retention tine (RT), signal to noise (S/N) ratio and number of data points in the peak. Area highlighted in green represents the area designated as the noise region that was used to calculated the S/N ratio. Insets in MS/MS spectra report mediator structure and fragment assignments.

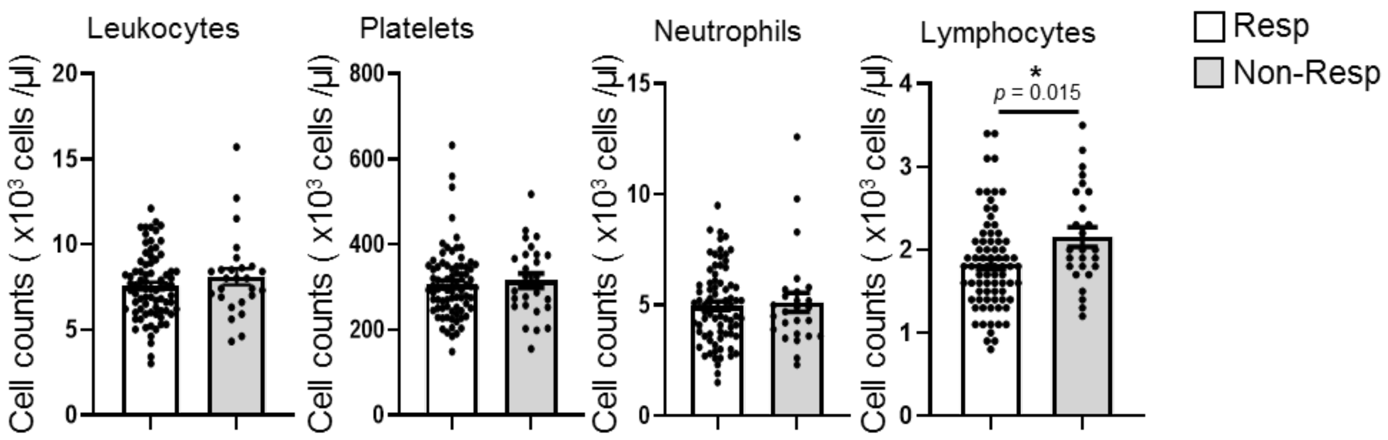

Supplementary Figure 5: Peripheral blood platelet and leukocyte counts in DMARD Responders and Non-Responders. Peripheral blood was collected in patients DMARD-responders (Resp) and DMARD-non-responders (Non-Resp) prior to DMARD treatment initiation and peripheral blood cell counts determined. Results are represented as means ± SEM. Results are from n = 78 for Resp and n=26 Non-Resp. * $p < 0.05$ using Mann–Whitney U test. Source data are provided as a Source Data file.

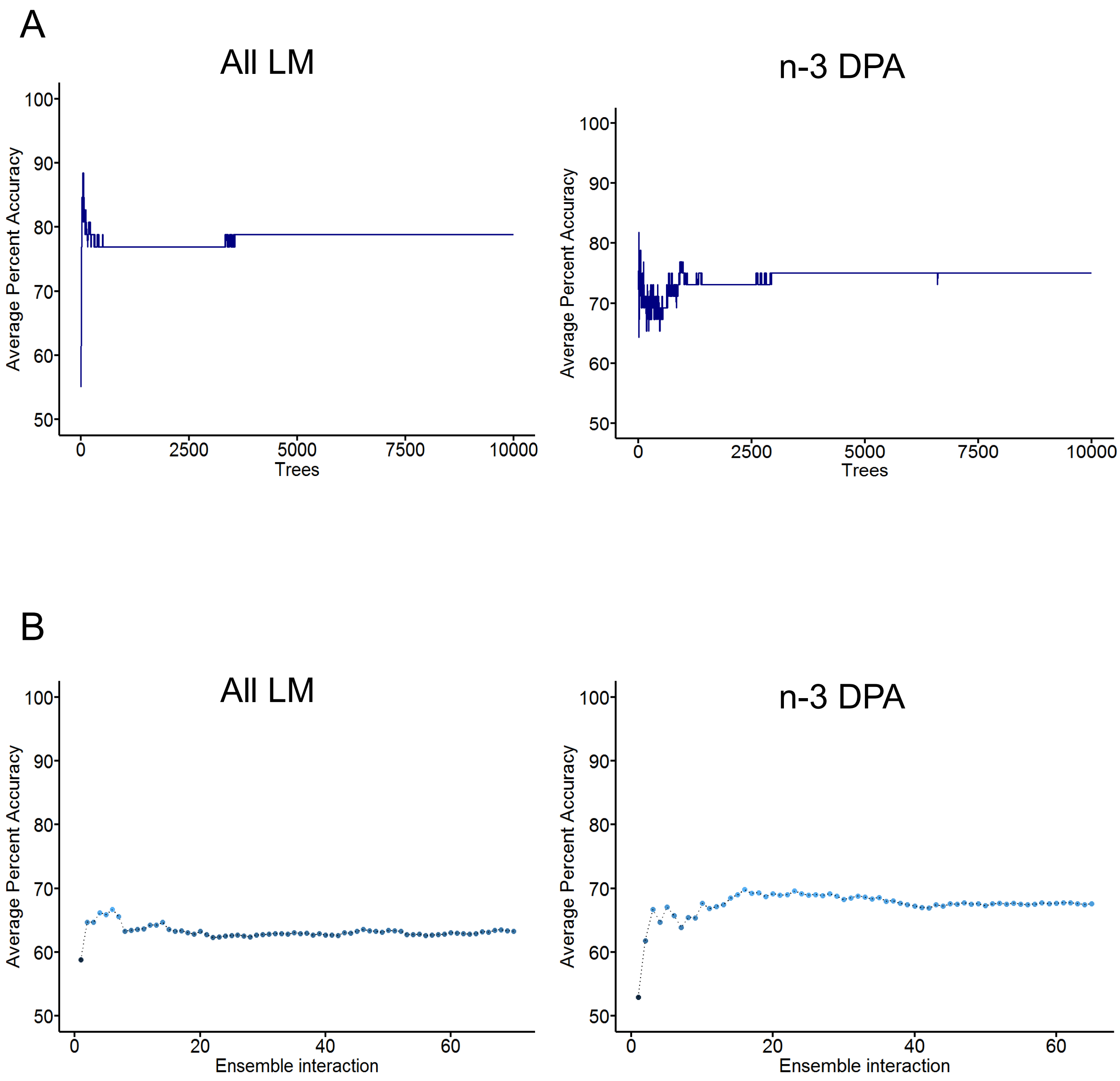

Supplementary Figure 6: Establishing the optimal parameters for RandomForest and SVM models. Representative results obtained for average percent accuracy for (A) RandomForest models and (B) SVM models by increasing the number of (A) decision trees (B) ensemble interactions.

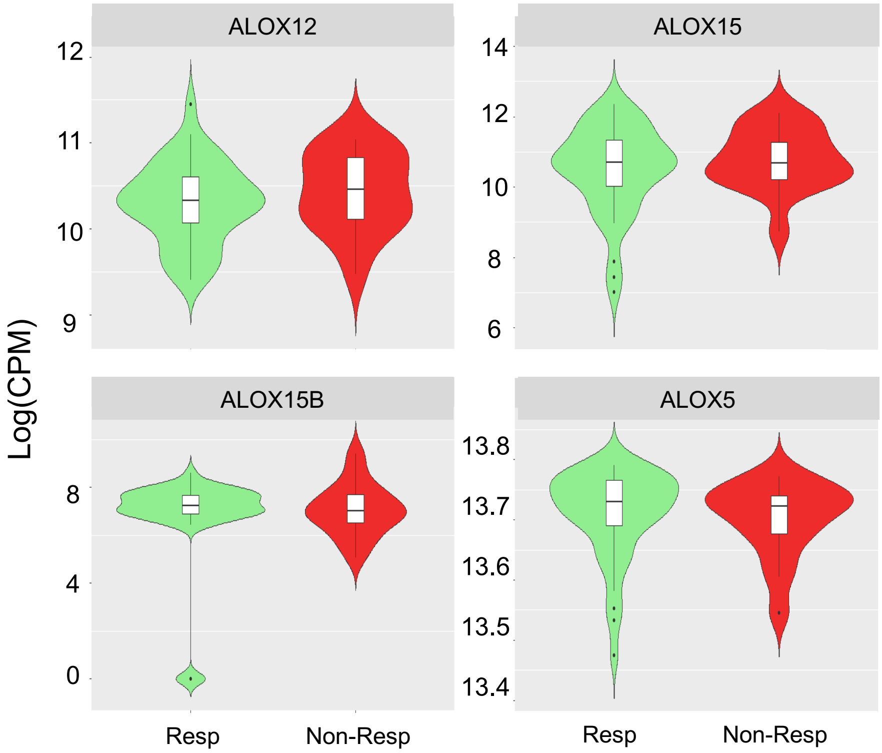

Supplementary Figure 7: Baseline expression of ALOX enzymes is essentially the same between DMARD-responders and DMARD-non-responders. Peripheral blood was collected in patients DMARD- responders (Resp) and DMARD-non-responders (Non Resp) prior to DMARD treatment initiation and the expression of ALOX enzymes was assessed. RNA count normalization and differential gene expression analysis were performed using the quasi-likelihood method of the Bioconductor R package “edgeR”. The data is expressed as the logarithm of counts per million (Log(CPM)), which indicates the number of reads mapping to a gene scaled by the number of reads you sequenced times one million. Results are represented as boxplots where the middle line is the median, the lower and upper hinges correspond to the first and third quartiles, the upper whisker extends from the hinge to the largest value no further than 1.5 × IQR from the hinge (where IQR is the inter-quartile range) and the lower whisker extends from the hinge to the smallest value at most 1.5 × IQR of the hinge, while data beyond the end of the whiskers are outlying points that are plotted individually (as indicated by the

geom_boxplot from the R package “ggplot2”). Results are representative of n = 43 for Resp and n=15 for Non-Resp. Source data are provided as a Source Data file.

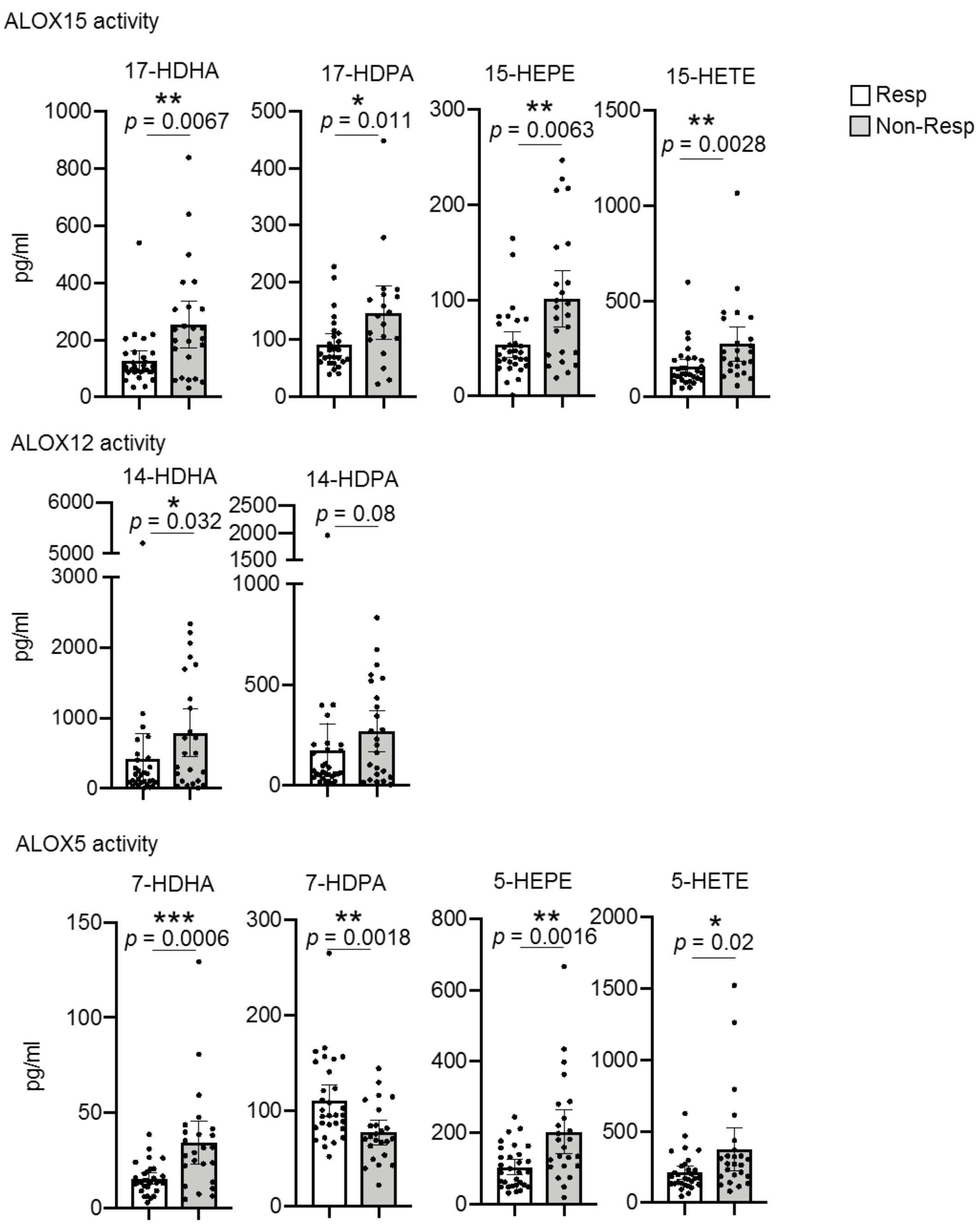

Supplementary Figure 8: Upregulation of monohydroxylated products from all four essential fatty acid metabolomes in plasma from non-responders when compared with responders prior to DMARD treatment initiation. Plasma was collected from RA patients prior the initiation of DMARD treatment and the activity of ALOX5 (7-HDHA, 7-HDPA, 5-HEPE and 5-HETE), ALOX12 (14-HDHA, and

14-HDPA) and ALOX15 (17-HDHA, 17-HDPA, 15-HEPE and 15-HETE) was investigated using LC-MS/MS based lipid mediator profiling. Results are expressed as mean ± 95% CI. *, $p<0.05$; **, $p<0.01$; ***, $p < 0.001$ using Mann-Whitney Test. n=30 for DMARD-responders (Resp) and n=24 for DMARD-non-responders (Non-Resp). Source data are provided as a Source Data file.

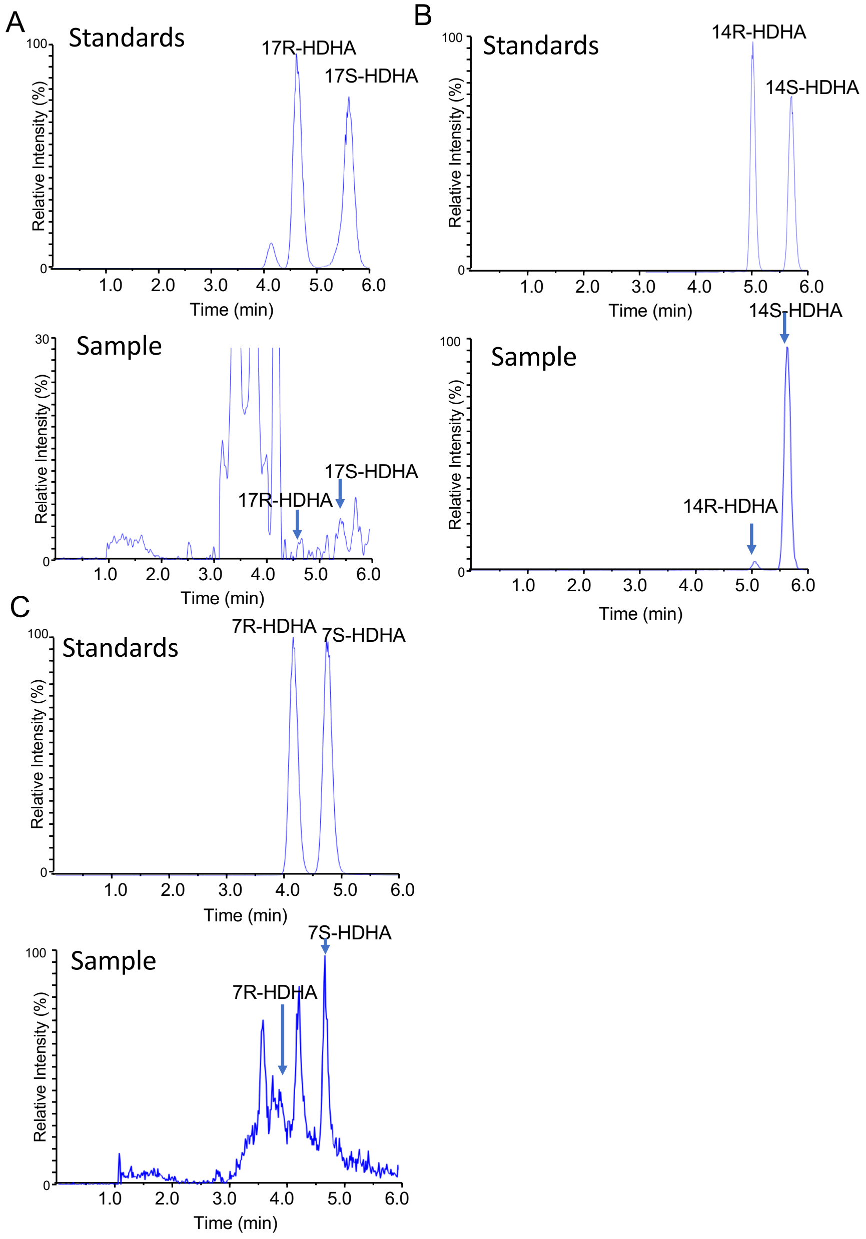

Supplementary Figure 9: Chiral chromatography of baseline plasma from DMARD responders and non-responders for DHA products. Plasma was extracted and relative abundance for each of the chiral isomers for (A) 17-HDHA, (B) 14-HDHA and (C) 7-HDHA were determined using chiral LC-MS/MS. (*Top panels*) chromatographic behaviour of standards, (*bottom samples*) representative chromatographic region of interest for the identification of the monohydroxy fatty acid epimers of interest. Results are representative of n=29 DMARD Responders and n=22 DMARD-non-responders from 3 distinct experiments.

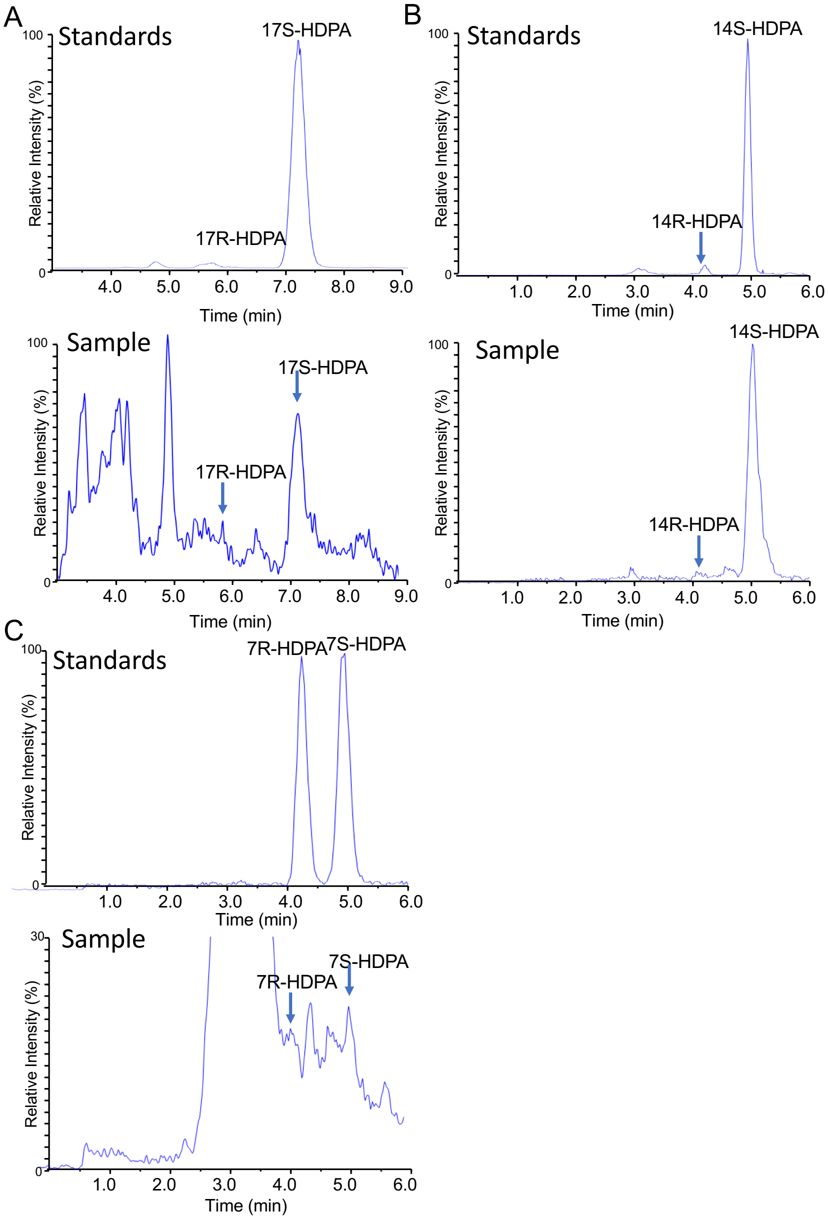

Supplementary Figure 10: Chiral chromatography of baseline plasma from DMARD responders and non-responders for n-3 DPA products. Plasma was extracted and relative abundance for each of the chiral isomers for (A) 17-HDPA, (B) 14-HDPA and (C) 7-HDPA were determined using chiral LC-MS/MS. (*Top panels*) chromatographic behaviour of standards, (*bottom panels*) representative chromatographic region of interest for the identification of the monohydroxy fatty acid epimers of interest. Results are representative of n=29 DMARD Responders and n=22 DMARD-non-responders from 3 distinct experiments.

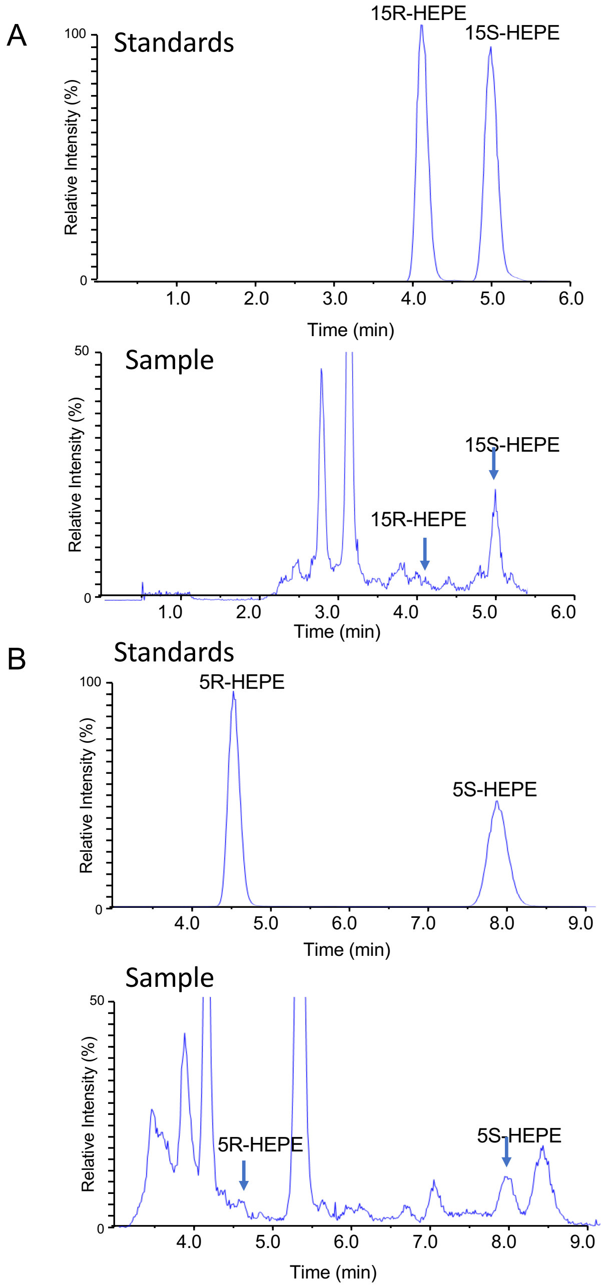

Supplementary Figure 11: Chiral chromatography of baseline plasma from DMARD responders and non-responders for EPA products. Plasma was extracted and relative abundance for each of the chiral isomers for (A) 15-HEPE, (B) 5-HEPE were determined using chiral LC-MS/MS. (*Top panels*) chromatographic behaviour of standards, (*bottom panels*) representative chromatographic region of interest for the identification of the monohydroxy fatty acid epimers of interest. Results are representative of n=29 DMARD Responders and n=22 DMARD-non-responders from 3 distinct experiments.

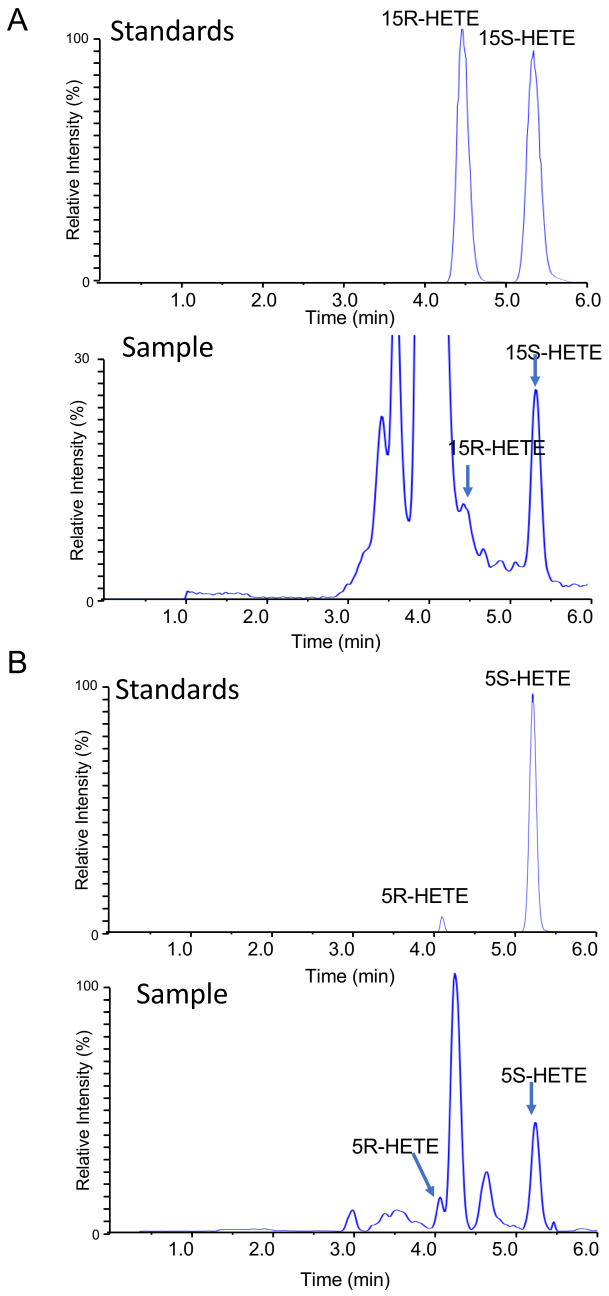

Supplementary Figure 12: Chiral chromatography of baseline plasma from DMARD responders and non-responders for AA products. Plasma was extracted and relative abundance for each of the chiral isomers for (A) 15-HETE, (B) 5-HETE were determined using chiral LC-MS/MS. (*Top panels*) chromatographic behaviour of standards, (*bottom panels*) representative chromatographic region of interest for the identification of the monohydroxy fatty acid epimers of interest. Results are representative of n=29 DMARD Responders and n=22 DMARD-non-responders from 3 distinct experiments.

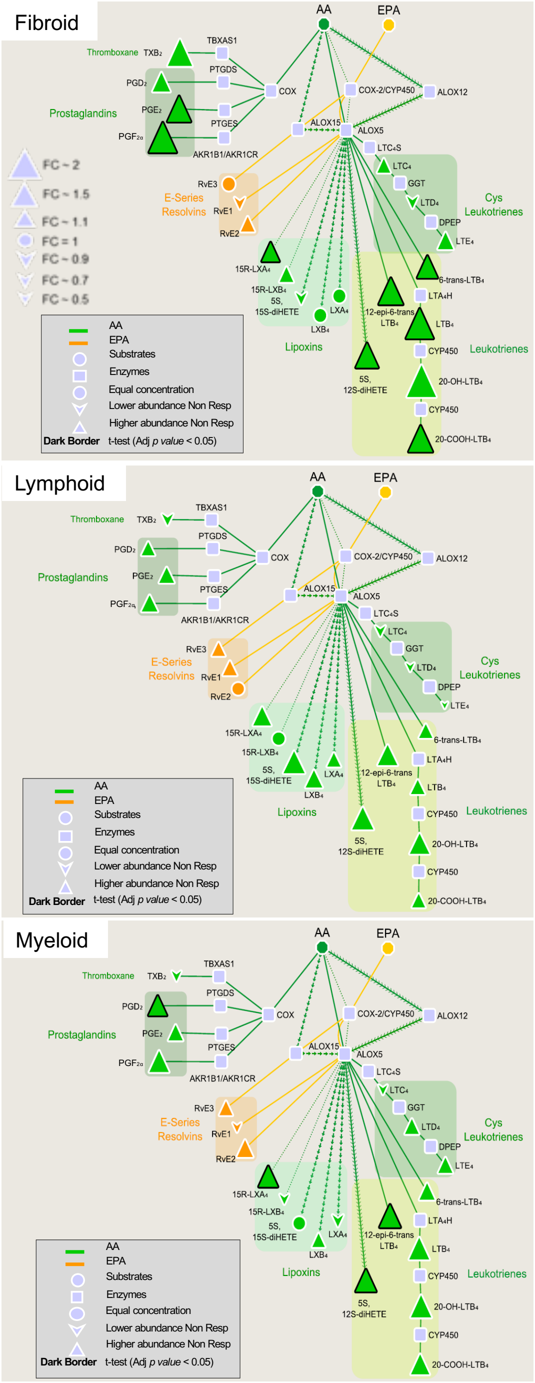

Supplementary Figure 13: Differential regulation of AA and EPA metabolomes in peripheral blood of DMARD-responders and DMARD-non-responders from patients with distinct pathotypes. Plasma was collected from RA patients prior the initiation of DMARD treatment. Lipid mediators were identified and quantified using LC-MS/MS based lipid mediator profiling and the differential expression of

mediators from the AA and EPA metabolomes was analysed for each RA pathotype in DMARD-non-responders (Non-Resp) when compared to DMARD-responders (Resp). Statistical differences between the normalised concentrations (expressed as the fold change) of the lipid mediators from the Non-Resp and Resp groups were determined using a two-sided t-test followed by multiple comparison correction using Benjamini-Hochberg procedure. Up or down regulated mediators are denoted with using upward and downward facing triangles, respectively, and on changes of the node's size. Bolded mediators represent statistical differences between the two groups when adjusted p value < 0.05. Results are representative of n= 18 for Fibroid Resp, n = 15 for Fibroid Non-Resp, n = 19 for Lymphoid Resp, n = 10 for Lymphoid Non-Resp, n = 22 for Myeloid Resp, n = 15 for Myeloid Non-Resp. Source data are provided as a Source Data file.

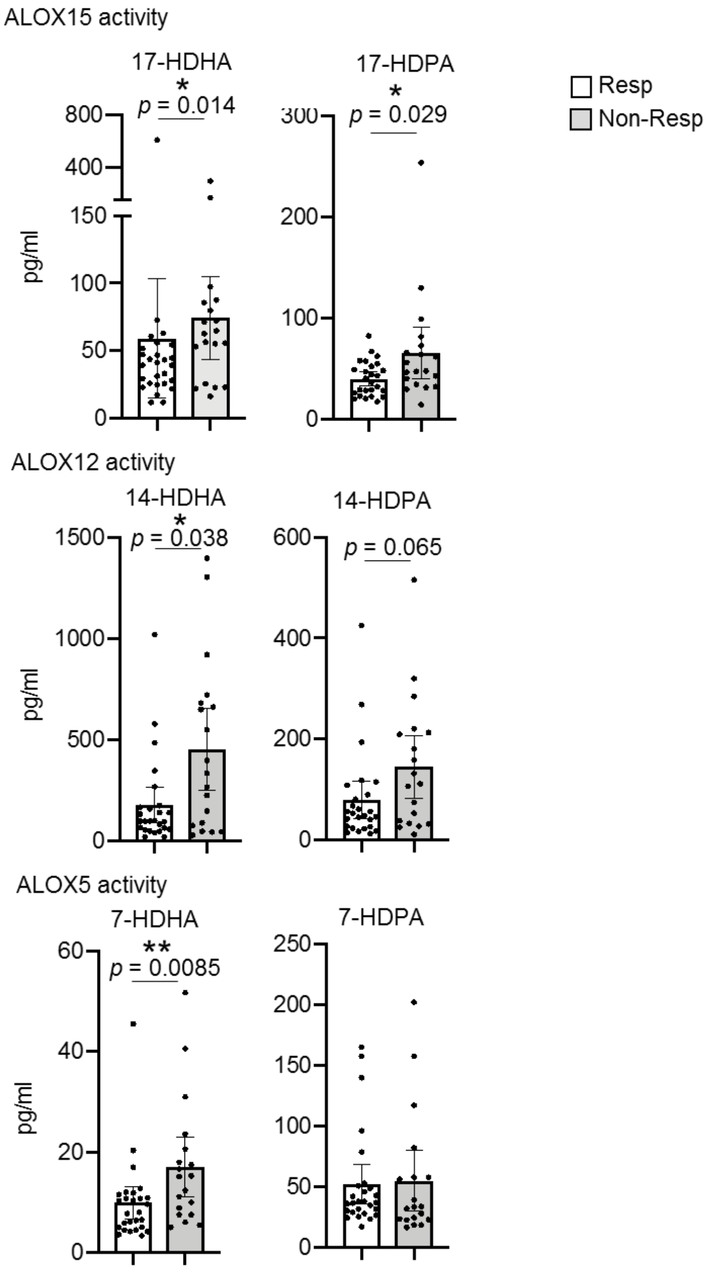

Supplementary Figure 14: Upregulation of monohydroxylated products from the DHA and n-3 DPA metabolomes in plasma from DMARD-non-responders when compared with DMARD-responders 6 months after DMARD treatment initiation. Plasma was collected from RA patients 6 months after the initiation of DMARD treatment and the activity of ALOX5 (7-HDHA and 7-HDPA), ALOX12 (14-HDHA and 14-HDPA) and ALOX15 (17-HDHA and 17-HDPA) was investigated using LC-MS/MS based lipid

mediator profiling. Results are expressed as mean ± 95% CI. *, $p<0.05$; **, $p<0.01$; using Mann-Whitney Test. n=26 for DMARD-responders (Resp) and n=19 for DMARD-non-responders (Non-Resp).

Source data are provided as a Source Data file.

Supplementary Tables

Supplementary Table 1: Cohort 1 patient demographics and clinical information

	DMARD - Responders (n=30)	DMARD- Non-Responders (n=24)
Pathotype (n)	Lymphoid (10), Fibroid (10), Myeloid (10)	Lymphoid (9), Fibroid (8), Myeloid (7)
Ethnicity (n)	Caucasian (19), Black (2), Indian (1), Caribbean (1), Asian (3), Bangladeshi (2), Black African (1)	Asian (1), Bangladeshi (1), Black (7), British (1), Caribbean (3), Caucasian (9), Filipino (1)
Gender (n)	Female (16), Male (14)	Female (21), Male(3)
Age at Recruitment – years	51 (±21)	56 (±9.5)
Onset	5.6 (±3.4)	6.0 (±3.42)
Currently smoking (%)	5 (16.7)	10 (43.5)
Co-Morbs Baseline (n)	Acne (1), Anaemia (1), Vitamin D deficiency (3), Hypercholesterolemia (2), Asthma (3), Hypertension (9), Osteoarthritis (2), Hypothyroidism (3), Gout (1), Shingles (1), Graves' disease (1), Kidney disease (1), TIA (1), Cardiovascular disease (1), Type 1 diabetes (1), Reactive iritis (1), Raised cholesterol (1), Hypothyroidism (3), Glaucoma (1), Thalassaemia (1), Poor vision (1), Heart surgery (1), Rubello in utero (1), Scleritis (1), COPD (1), Enlarged prostate (1), Sinusitis (1), Peptic ulcer (1), Lower back pain (1), Acute MI (1), Ischaemic heart disease (1), Ca bladder (1)	Hypercholesterolemia (2), Asthma (5), Hypertension (12), Osteoarthritis (2), Hypothyroidism (1), COPD (1), Ischaemic heart disease (2), Osteoporosis (1), Sickle cell trait (1), IBS (2), Cervical spondylitis (1), Psoriasis (1), Fatty liver (1), Renal impairment (1), Coronary artery disease (1), Angina (1), Dyslipidaemia (1), Menorrhagia (1), Multiple sclerosis (1), Rectal incontinence (1), Detrusor instability (1), Meniscus tear/knee (1), Depression (1), Spina Bifida occulta (1), Axonal neuropathy (1), BCC (1), Gastritis (1), Heart valve repaired (1), Hysterectomy (1), foot & shoulder surgery (1), Endometriosis (1), Hay fever (1), Hysterectomy (1)
Concomitant Med. Baseline (n)	Tetracycline (1), Ferrous sulphate (1), Co-codamol (6), NSAIDs (3), Calcium vitamin D (2), Inhaler (1), Candesartan (1), Etoricoxib (1), Furosemide (1), Ramipril (2), Levothyroxine (1), Dihydrocodeine (1), Lisinopril (1), Lansoprazole (1), Bisoprolol (1), Tamsulosin (1), Carbimazole (1), Aspirin (2), Adcal (1), Simvastatin (5), Ibuprofen (4), Codeine (1), Losartan (1), Citalopram (1), Naproxen (5), Metformin (1), Lantus (1), Lacri-lube (1), Atenolol (1), Irbesartan (1), Amlodipine (2), Timolol (1), GTN spray (1), Frusemide (1), Celecoxib (1), Omeprazole (2), Budromide (1), Salbutamol (3), Diclofenac (1), Finasteride (1), Thyroxine (1), Talisartan (1)	Co-codamol (3), NSAIDs (3), Calcium vitamin D (3), Inhaler (1), Candesartan (2), Furosemide (1), Ramipril (3), Dihydrocodeine (1), Allopurinol (1), Lansoprazole (4), Aspirin (4), Simvastatin (5), Ibuprofen (2), Losartan (1), Ca-antagonist (1), Atenolol (2), Amlodipine (2), Alendronate (1), GTN spray (1), Pregabalin (2), Frusemide (1), Omeprazole (1), Salbutamol (1), Diclofenac (4), Thyroxine (2), Isosorbide (1), Adizem (1), Clopidogrel (2), Atorvastatin (1), Ivabradine (1), Co-tenidone (1), Nicorandil (1), Paracetamol (4), Tramadol (5), Amitriptyline (5), Piroxicam (1), Quinine (1), Bendroflumethiazide (3), Budesonide (2), Formoterol (1), Cetirizine (1), Arthrotec (1), Insulin (1), Doxazosin (2), Seretide (2), Spiriva (1), Arcoxia (1), Metformin (2), Glimepiride (1), Fluoxetine (1), Anti-hyper (1), Lyrica (1), CaD3 (1), Peppermint (1), Tiotropium (1), Detrusitol (1), Docusate (1), Voltron (1), HRT, (1), Aminophylline (1), Xolair (1), Ventolin (1), Oramorph (1), Temazepam (1)
Concomitant Med. 6mth (n)	Ferrous sulphate (1), MTX (28), Folic acid (8), ASA (21), Co-codamol (6), HCQ (8), Prednisolone (6), NSAIDs (3), Calcium vitamin D (3), Azathioprine (1), Salbutamol (3), Inhaler (1), Candesartan (1), Etoricoxib (1), Furosemide (1), Ramipril (2), Levothyroxine (1), Dihydrocodeine (1), Lisinopril (1), Allopurinol (1), Lansoprazole (1), Bisoprolol (1), Tamsulosin (1), Carbimazole (1), Aspirin (2), Adcal (1), Simvastatin (5), Ibuprofen (4), Codeine (1), Losartan (1), Citalopram (1), Naproxen (5), Metformin (1), Lantus (1), Lacri-lube (1), Atenolol (1), Irbesartan (1), Amlodipine (3),Timolol (1), Frusemide (1), Celecoxib (1), Omeprazole (2), Budromide (1), Diclofenac (1), Thyroxine (1), GTN spray (1), Finasteride (1)	MTX (18), Folic acid (6), ASA (9), Co-codamol (2), HCQ (11), Prednisolone (5), NSAIDs (3), Calcium vitamin D (3), Salbutamol (1), Inhaler (1), Candesartan (1), Furosemide (1), Ramipril (3), Dihydrocodeine (1), Allopurinol (1), Lansoprazole (5), Aspirin (4), Simvastatin (5), Ibuprofen (2), Losartan (1), Atenolol (2), Amlodipine (2), Frusemide (1), Omeprazole (1), Diclofenac (4), Thyroxine (2), Atorvastatin (1), GTN spray (1), Ca-antagonist (1), Alendronate (1), Pregabalin (2), Co-tenidone (1), Leflunomide (2), Paracetamol (5), Tramadol (5), Amitriptyline (4), Piroxicam (1), Quinine (1), Spironolactone (1), Bendroflumethiazide (2), Clopidogrel (2), Sulfasalazine (2), Budesonide (2), Formoterol (1), Cetirizine (1), Insulin (1), Doxazosin (2), Isosorbide (1), Adizem (1), Ivabradine (1), Nicorandil (1), Seretide (2), Spiriva (1), Arcoxia (1), Metformin (2), Glimepiride (1), Fluoxetine (1), Anti-hyper (1), Lyrica (1), Peppermint (1), Tiotropium (1), CaD4 (1), Detrusitol (1), Docusate (1), Voltarol (1), Aminophylline (1), Xolair (1), Ventolin (1), Co-Amoxiclav (1), Calcichew (1)
DMARD Treatment (n)	MTX/ASA/HCQ (2), MTX/ASA (21), MTX/HCQ (3), ASA/HCQ (1), MTX (1)	MTX/ASA/HCQ (2), MTX/ASA (11), MTX/HCQ (6), MTX (1), ASA (1), HCQ (2)
Recent Steroid Therapy Baseline (n)	No (25), Yes (5)	No (19), Yes (3)
Steroid Treatment Baseline (n)	Depo-Medro (5)	Pred. (3)
Recent Steroid Therapy 6mth (n)	No (13), Yes (15)	No (8), Yes (13)
Steroid Treatment 6mth (n)	Pred (15)	Depo-Medro (1), Pred (11)
ESR Baseline (mm/hr)	28 (±23)	41 (±28)

CRP Baseline (mg/l)	14 (±17)	24 (±37)
CCP Baseline (UI/L)	210 (±201)	249 (±255)
RF Baseline (UI/L)	87 (±120)	93 (±150)
ESR 6mth (mm/hr)	7 (±7)	30 (±14)
CRP 6mth (mg/l)	7 (±5)	15 (±18)
Tiredness VAS Baseline (1-100)	34 (±29)	48 (±26)
Pain VAS Baseline (1-100)	50 (±29)	62 (±23)
Pt. VAS Global Health baseline (1-100)	67 (±24)	72 (±19)
Physician VAS Global Assess. Baseline (1-100)	58 (±)21	65 (±20)
Tiredness VAS 6mth (1-100)	28 (±27)	64 (±17)
Pain VAS 6mth (1-100)	12 (±14)	66 (±22)
Pt. VAS Global Health 6mth (1-100)	17 (±20)	71 (±24)
Physician VAS Global Assess. 6mth (1-100)	10 (±12)	63 (±22)
Tender Joints Baseline (Number/28)	12 (±7)	13 (±9)
Swollen Joints Baseline (Number/28)	7 (±5)	8 (±5)
Tender Joints 6mth (Number/28)	1 (±1)	17 (±8)
Swollen Joints 6mth (Number/28)	1 (±1)	8 (±4)
HAQ Baseline (Max 38)	1.41 (±0.68)	1.83 (±0.60)
HAQ 6mth (Max 38)	0.57 (±0.73)	1.79 (±0.51)
DAS28 Baseline (0-10)	5.58 (±0.98)	5.81 (±1.08)
DAS28 6mth (0-10)	1.86 (±0.65)	6.17 (±0.98)
Delta DAS28 (Difference between 2 time points)	-3.72 (±1.13)	0.36 (±0.98)
US Synovial Thickness (12max) Baseline (number/36)	18 (±7)	15 (±9)
US Power Doppler (12max) Baseline (number/36)	7 (±6)	6 (±8)
US Synovial Thickness (Biopsied Joint) Baseline (number/3)	2 (±0.5)	3 (±1)
US Power Doppler (Biopsied Joint) Baseline (number/3)	1 (±1)	2 (±1)
US Synovial Thickness (12max) 6mths (number/36)	6 (±4)	7 (±8)
US Power Doppler (12max) 6mths (number/36)	2 (±3)	3 (±5)
Radiographic Erosion (n)	No (23), Yes (4)	No (20), Yes (2)

Erythrocyte sedimentation rate = ESR, C-reactive protein = CRP, Anti-cyclic citrullinated peptide = CCP, rheumatoid factor =RF, VAS = visual analogue scale, HAQ = Health Assessment Questionnaire, DAS28 = disease activity score-28, US = ultrasound, MTX = methotrexate, ASA = aspirin, HCQ = hydroxychloroquine, LEF = Leflunomide.

Supplementary Table 2: Chromatography and MS-MS spectral criteria employed in the identification of lipid mediators.

	Standard RT	Reference diagnostic ions	Identified RT	S/N ratio	Data points	Identified diagnostic ions
DHA Bioactive Metabolome						
RvD1	11.1	**375**, 357, 331, 313, 295, **277**, **233**, 215, **171, 113**	11.1	12	7	**375**, 357, 331, 295, **277**, 215, **171**, **113**
RvD2	10.6	**375**, 357, 331, 313, 295, 287, **247**, **233**, 215, 203 **141**, **113**	10.6	8	9	**375**, 357, 331, 313, 295, 287, **247**, **233**, 215, 203, **113**
RvD3	10.7	**375**, 357, 313, 295, 259, 215, **181**, **165**, 147, 137, **101**	10.7	5	9	**375**, 357, 313, 295, 259, 215, **165**, 137
RvD4	12.1	**375**, 357, 339, 331, 313, 295, **277**, 255, 225, 215, 113, **101**	12.1	7	11	**375**, 357, 339, 331, 313, 295, **277**, 225, 215, **101**
RvD5	13.2	**359**, 341, 323, 315, 297, 279, 245, 243, 227, **217**, 199, **141**, **113**	13.2	5	6	**359**, 341, 315, 297, 279, 227, 199, **141**, **113**
RvD6	13.8	**359**, 341, 315, 297, **289**, 279, 245, 243, 227, 217, 199, **101**	13.8	5	4	**359**, 341, 315, **289**, 279, 243, 227, 217, 199, **101**
17R-RvD1	11.3	**375**, 357, 331, 313, 295, 243, 215, **171**, 135, 123	11.3	4	6	**375**, 357, 331, 313, 295, 243, 215, **171**, 123
17R-RvD3	10.6	**375**, 357, 339, 313, 295, 259, 191, **165**, 147, 137, **101**	10.6	3	8	**375**, 357, 339, 313, 295, 259, 191, **165**, 147, 137
PD1	13.3	**359**, 341, 323, 315, 297, 243, 217, **206**, 199, **181**, 159, **153**	13.3	5	6	**359**, 341, 323, 315, 297, 243, 217, 199, **153**
17R-PD1	12.5	**359**, 341, 323, 315, 297, 279, **261**, 217, **206**, **181**, 177, 153	12.5	6	5	**359**, 341, 323, 315, 297, 279, 217, **206**, **181**, 153
PDx	13.2	**359**, 341, 323, 315, 297, 279, 243, 217, **206**, **181**, 159, **153**, 119	13.2	4	5	**359**, 341, 315, 297, 279, 243, 217, 159, **153**
22-OH-PD1	9.8	**375**, 357, 331, 313, 295, **289**, **261**, 243, 217, 199, **181**, **153**, 137	9.8	31	11	**375**, 357, 331, 313, 295, **289**, 243, 199, **153**
PCTR1	9.5	**650**, 632, 503, **343**, **308**, 264, 227	9.5	4	6	**650**, 632, **343**, **308**, 264, 227
PCTR2	8.5	**521**, 503, **343**, 325, **231**, 213, **179**, 161	8.5	6	6	**521**, 503, **343**, 325, **231**, 213, **179**, 161
PCTR3	10	**464**, 446, **343**, 325, **231**, 213, 187, **121**	10	7	6	**464**, 446, **343**, **231**, 213, 187, **121**
MaR1	13.4	**359**, 341, 323, 315, 297, 279, **221**, **141**, **113**	13.4	5	5	**359**, 341, 323, 315, 297, 279, **221**, **141**, **113**
MaR2	14.1	**359**, 341, 323, 315, 297, **249**, **221**, **191**, **167**, 149	14.1	11	7	**359**, 341, 323, 315, 297, **221**, **191**, **167**, 149
22-OH-MaR1	9.5	**375**, 357, 339, 331, 313, 295, **262**, 244, **221**, 177, **153**	9.5	4	9	**375**, 357, 339, 331, 313, 295, 244, **221**, **153**
14-oxo-MaR1	13.3	**357**, 339, 321, 313, 295, **248**, **221**, **141**, **113**	13.3	24	5	**357**, 339, 321, 313, 295, **248**, **141**, **113**
7S,14S-diHDHA	13.5	**359**, 341, 323, 315, 297, 279, **221**, **141**, **113**	13.5	5	11	**359**, 341, 323, 315, 297, 279, **221**, **141**, **113**
4,14-diHDHA	14.1	**359**, 341, 323, 315, 297, 279, **257**, 239, **221**, 203, 159, **109**	14.1	9	8	**359**, 341, 323, 315, 297, 279, **221**, 159
MCTR1	9.4	**650**, 632, **418**, **308**, **235**, **109**	9.4	14	6	**650**, 632, **418**, **308**, **235**, **109**
MCTR2	8.5	**521**, 503, **343**, 325, **191**, **179**, 161	8.5	5	5	**521**, 503, **343**, 325, **191**, **179**, 161
MCTR3	10	**464**, 446, **343**, 325, **235**, **205**, **191**, 187, 147	10	13	7	**464**, 446, **343**, 325, 235, **205**, **191**, 147
n-3 DPA Bioactive Metabolome						
RvT1	10.4	**377**, 359, 333, **319**, 315, 297, **233**, 193, **143**	10.4	10	6	**377**, 359, 333, **319**, 315, 297, 193, **143**
RvT2	11.3	**377**, 359, 341, 333, 315, 297, **263**, **233**, **197**, **143**	11.3	6	5	**377**, 359, 341, 333, 315, 297, **263**, **233**
RvT3	11.7	**377**, 359, 341, 333, 315, 297, **255**, **233**, 215, **173**,**143**	11.7	8	9	**377**, 359, 333, 315, 297, **233**, 215, **173**, **143**
RvT4	13.7	**361**, 343, 325, 317, 299, 233, 221, **217**, **211**, 193, **143**	13.7	7	6	**361**, 343, 325, 317, 299, 233, 221, **143**

RvD1$_{n-3\ DPA}$	11.4	**377**, 359, 341, 333, 315, 297, 289, **279,** 261, 235, **233**, 125	11.4	13	9	**377**, 359, 341, 333, 315, 297, 289, **279,** 261, 245, **233**
RvD2$_{n-3DPA}$	11	**377,** 359, 341, 333, 315, 297, 261, **233**, 215, **143,** 125	11	4	11	**377**, 359, 341, 333, 315, 297, **233**, **143**, 125
RvD5$_{n-3DPA}$	13.6	**361**, 343, 325, 317, 299, 281, **263**, **217**, 201, 199, **143**	13.6	6	6	**361**, 343, 325, 317, 299, 281, **263**, **217**, 201, **143**
PD1$_{n-3\ DPA}$	13.7	**361,** 343, 325, 317, 299, 281, **263**, 219, **183**	13.7	6	5	**361**, 343, 325, 317, 299, 281, **263**, 219**, 183**
10S,17S-diHPDA	13.6	**361,** 343, 325, 317, 299, 281, **263,** 245, 219, **183, 155**	13.6	4	10	**361,** 343, 325, 317, 299, 281, **263,** 245, 219, **183, 155**
MaR1$_{n-3\ DPA}$	13.7	**361,** 343, 317, 299, 281, **223**, 205, 179, 161, **143, 115**	13.7	5	11	**361**, 343, 317, 299, 281, **223**, 205, 161 , **115**
EPA Bioactive Metabolome						
RvE1	8.2	**349**, 331, 305, 287, 273, 269, 255, 205, **195,** 177, 161, 151	8.2	6	7	**349**, 331, 305, 287, 273, 269, 205, **195,** 177
RvE2	12	**333,** 315, 297, 289, **275,** 271, 257, 253, 231, **217**	12	8	9	**333**, 315, 297, 289, 257, 253, **217**
RvE3	13.5	**333,** 315, 297, 289, 271, 259, 253, **245,** 201	13.5	8	5	**333**, 315, 297, 289, 271, **245,** 201
AA Bioactive Metabolome						
LXA$_4$	11.2	**351,** 333, 307, 289, **279,** 271, **251, 235,** 233, 207, 189, **145**	11.2	4	5	**351**, 333, 307, 289, 271, **251**, **235**, 189
LXB$_4$	10.5	**351,** 333, 315, 307, 289, 271, **251,** 243, **235**, **221,** 207, 189, 177, 159, **115**	10.5	11	5	**351**, 333, 315, 307, 289, 271, **221**, 189, **115**
5S,15S-diHETE	13.2	**335**, 317, 299, 291, 273, **263**, 255, **235,** 201, 191, 173, **115**	13.2	7	4	**335**, 317, 299, 291, 273, **235**, 201, 191, 173, **115**
15R-LXA$_4$	11.4	**351,** 333, 307, 289, **279**, 271, **251, 235**, 217, 207, 199, **145**	11.4	8	8	**351**, 333, 307, 289, **279**, 271, **251**, **235**, **145**
15R-LXB$_4$	11.2	**351,** 333, 307, 297, 271, 261, **251, 235**, **221,** 215, 203, 177, **115**	11.2	4	8	**351**, 333, 307, 261, **235**, **221**, 203, 189, **115**
LTB$_4$	13.7	**335,** 317, 299, 291, 273, 255, **219,** 205, **195,** 179, 177, 161, 151, 133, **115**	13.7	37	9	**335**, 317, 299, 291, 273, 255, 205, **195**, 179, 161, **115**
5S,12S-diHETE	13.8	**335,** 317, 299, 291, 273, **219**, 205, **195**, 179, **115**	13.8	7	4	**335**, 317, 299, 291, 273, **219**, 205, **195**, 179
6-trans-LTB$_4$	13.1	**335,** 317, 291, 255, **219, 195,** 179	13.1	34	7	**335**, 317, 291, 273, 255, **219**, **195**, 179
12-epi-6-trans-LTB$_4$	13.3	**335,** 317, 299, 291, 273, 255, **219, 195,** 161, **115**	13.3	48	7	**335**, 317, 299, 291, 273, 255**, 219**, **195**, 161, **115**
20-OH-LTB$_4$	8.7	**351,** 333, 315, 289, 271, **195,** 179, 177, 151	8.7	38	8	**351**, 333, 315, 289, 271, **195,** 179, 177, 151
20-COOH-LTB$_4$	8.3	**365,** 347, 329, 321, 303, 285, **249,** 205, **195,** 177, 161, **115**	8.3	24	8	**365**, 347, 329, 321, 303, 285, **249**, 205, 1**95**, 177, 161, **115**
LTC$_4$	10	**626,** 608, 582, **497**, 479, **319, 308,** 301, 171	10	17	7	**626**, 608, 582, **319**, **308,** 301, 171
LTD$_4$	9	**497,** 479, 301, **189, 179,** 135, **116**	9	30	8	**497,** 479, 301, **189**, **179,** 135, **116**
LTE$_4$	10.4	**440, 319,** 301, 283, **189, 131**	10.4	33	6	**440, 319,** 301, 283, **189**, **131**
PGD$_2$	10.6	**351,** 333, 307, 289, **279,** 271, **251**, 233, 217, 189	10.6	41	8	**351,** 333, 307, 289, **279**, 271, 233, 189
PGE$_2$	10.5	**351,** 333, 315, 289, **279,** 271, **251**, 235, 189	10.5	29	6	**351**, 333, 315, 289, **279**, 271, 235, 189
PGF$_{2a}$	10.8	**353,** 335, 317, **309,** 291, **281,** 273, 263, 235, 219, 193, 191, 173	10.8	8	8	**353**, 335, 317, **309**, 291, 273, 263, 193, 191
TxB$_2$	10	**369,** 325, 307, 289, **269,**195, 177, **169**, 125	10	8	8	**369**, 325, 307, 289, **269**, 195, 177, **169**, 125

RT = Retention time, S/N = Signal to Noise. Ions marked in **Bold** denote fragments

Supplementary Table 3: Baseline peripheral blood lipid mediator profiles in RA patient cohort 1.

	Q1	Q3	DMARD Responders			DMARD Non-Responders		
**DHA bioactive metabolome**			Mean	±	SEM	Mean	±	SEM
RvD1	375	233	0.92	±	0.44	2.20	±	0.61
RvD2	375	141	1.75	±	0.73	1.52	±	1.52
RvD3	375	147	0.52	±	0.15	1.06	±	0.24
RvD4	375	101	1.40	±	0.53	5.70	±	1.19
RvD5	359	199	7.90	±	1.06	8.17	±	1.34
RvD6	359	101	1.82	±	0.66	1.30	±	0.40
17R -RvD1	375	233	2.43	±	1.62	3.69	±	1.92
17R -RvD3	375	147	0.13	±	0.05	0.60	±	0.20
PD1	359	153	0.78	±	0.27	0.99	±	0.33
PDX	359	153	0.63	±	0.21	1.39	±	0.46
17R-PD1	359	153	0.31	±	0.13	0.74	±	0.40
22-OH-PD1	375	153	2.36	±	2.36	1.91	±	1.46
PCTR1	650	231	0.13	±	0.13	0.00	±	0.00
PCTR2	521	231	1.48	±	0.65	2.10	±	1.80
PCTR3	464	231	16.87	±	6.74	4.75	±	4.63
MaR1	359	221	0.00	±	0.00	0.00	±	0.00
MaR2	359	191	0.47	±	0.20	0.56	±	0.31
7S,14S-diHDHA	359	221	3.21	±	1.57	3.65	±	1.73
4S,14S-diHDHA	359	101	0.98	±	0.48	4.29	±	1.13
22-OH-MaR1	375	221	4.64	±	3.77	5.31	±	3.98
14-oxo-MaR1	357	248	0.73	±	0.38	2.65	±	0.78
MCTR1	650	191	0.16	±	0.16	0.38	±	0.21
MCTR2	521	191	1.42	±	0.46	1.44	±	1.04
MCTR3	464	191	8.45	±	2.67	6.57	±	2.92
**n-3 DPA bioactive metabolome**								
RvT1	377	193	0.92	±	0.69	0.66	±	0.31
RvT2	377	197	2.97	±	1.31	2.26	±	0.71
RvT3	377	197	0.49	±	0.17	0.60	±	0.19
RvT4	361	211	0.48	±	0.13	0.78	±	0.18
$RvD1_{n-3\ DPA}$	377	143	4.73	±	0.78	4.50	±	0.58
$RvD2_{n-3\ DPA}$	377	261	3.64	±	1.22	2.32	±	0.96
$RvD5_{n-3DPA}$	361	199	3.39	±	1.32	3.06	±	1.26
$PD1_{n-3\ DPA}$	361	155	0.72	±	0.18	1.26	±	0.38
10S, 17S-diHDPA	361	155	0.69	±	0.33	2.11	±	0.49
$MaR1_{n-3\ DPA}$	361	249	0.07	±	0.07	1.25	±	0.49
**EPA bioactive metabolome**								
RvE1	349	161	1.98	±	0.56	1.94	±	0.83
RvE2	333	199	0.30	±	0.25	0.71	±	0.34
RvE3	333	201	2.89	±	0.78	3.96	±	0.95
**AA bioactive metabolome**								
LXA_4	351	115	0.72	±	0.28	0.96	±	0.60
LXB4	351	221	5.14	±	3.99	3.05	±	1.47
15R-LXA_4	351	115	0.97	±	0.26	5.71	±	2.27
15R-LXB_4	351	221	3.90	±	1.09	9.13	±	2.60
5S,15S-diHETE	335	115	36.90	±	7.28	46.68	±	7.59
LTB_4	335	195	65.59	±	13.57	115.09	±	37.71
5S,12S-diHETE	335	195	11.42	±	9.03	12.69	±	4.52
6-trans-LTB_4	335	195	2.93	±	0.57	5.37	±	1.82
12-epi-6-trans-LTB_4	335	195	3.56	±	0.73	7.89	±	2.10
20-OH-LTB_4	351	195	61.80	±	20.51	147.89	±	50.54
20-COOH-LTB_4	369	195	12.86	±	5.40	22.68	±	7.65
LTC_4	626	189	12.34	±	3.16	18.59	±	8.10
LTD_4	497	189	7.42	±	2.20	6.93	±	2.71
LTE_4	440	189	32.99	±	7.84	72.85	±	16.13
PGD_2	351	189	9.00	±	2.47	15.22	±	7.22
PGE_2	351	189	13.39	±	5.30	18.75	±	5.76
PGF_{2a}	353	193	29.26	±	15.96	27.65	±	7.49
TXB_2	369	169	33.46	±	13.46	83.09	±	25.63

Results are expressed pg/ml. n = 30 for DMARD Responders and n = 22 DMARD Non-Responders.

Supplementary Table 4: Cohort 2 patient demographics and clinical information

	DMARD Responders (n=36)	DMARD Non-Responders (n=22)
Pathotype (n)	Lymphoid (9), Fibroid (8), Myeloid (13), Ungraded (6)	Lymphoid (2), Fibroid (7), Myeloid (9), Ungraded (4)
Ethnicity	Caucasian (18), Asian (3), Indian (1), Chinese (1), Black Caribbean (1), Bengali (1), Somalian (1), Black African (2), Afro-Caribbean (1), Bangladeshi (2), Sudanese (1), Pakistan (1), Korean (1), Caribbean (1)	Caucasian (8), Asian (5), Black African (1), Bangladeshi (2), Mixed Greek (1), Black (1), Pakistani (1), British (1), African (1)
Gender (n)	Female (13), Male (22)	Female (19), Male (3)
Age at Recruitment – years	51 (±14)	50 (±10.4)
Onset	4.8 (±2.7)	4.8 (±3.1)
Currently smoking (%)	7 (20)	6 (22.3)
Co-Morbs Baseline (n)	Osteoarthritis (1), Acne (1), Anaemia (1), Asthma (5), Depression (3), Hypothyroidism (2), Gastro-oesophageal reflux disease (1), anal fistula (1), Hay fever (1), Varicose veins (1), Hypertension (10), Glaucoma (2), Psoriasis (1), Gastric ulcers (1), Diverticulitis (1), Allergy to penicillin (1), Hypercholesterolemia (5), Erectile dysfunction (1), Ischaemic heart disease (2), Diabetes (3), Coronary heart disease (1), Enlarged prostate (1), Carpal tunnel syndrome (1), Low vitamin D (1), GORD (1), Ca prostate (1)	Osteoarthritis (3), Asthma (4), Depression (2), Hypertension (6), Hypercholesterolemia (6), Diabetes (5), Osteopenia (1), Low back pain (1), Anaemia (1), Psoriasis (3), Gout (1), heart block (1), hearing loss (1), laiden deficiency (1), Polcystic ovaries (1), Chest infection (1), Gastritis (1), Fibromyalgia (1), Renal tubular acidosis (1),
Concomitant Med. Baseline (n)	NSAIDs (1), Tetracycline (1), Ferrous sulfate (2), Fluoxetine(1), Fluticasone (1), Salbutamol (1), Lansoprazole (2), Levothyroxine (2), Naproxen (5), Gaviscon (1), Movicol (1), Ibuprofen (5), Amlodipine (6), Timolol (1), Latanoprost (1), Omeprazole (2), Tramadol (1), Diclofenac (2), Perindopril (2), Etoricoxib (1), Indapamide (1), Calcium (2), Irbesartan (1), Vitamin D (2), Sibicol (1), Bendroflumethiazide (2), Simvastatin (6), Co-codamol (5), Metformin (3), Ramipril (2), Repaglinide (1), Insulin (2), Sildenafil (1), Aspirin (3), Dipyridamole (1), Isosorbide (1), Calci-chew (1), Doxazosin (1), Prednisolone (1), Lisinopril (2), Atenolol (2), Gabapentin (1), Symbicort (1), Paracetamol (1), Solgar (1), Glucosamine (1), Multivitamin (1), Senokot (1), Amitriptyline (1), Flixotide (1), Ventolin (1), Phyllocontin (1), Tamsulosin (1), Arcoxia (1)	Salbutamol (2), Lansoprazole (3), Levothyroxine (1), Naproxen (2), Ibuprofen (3), Amlodipine (2), Omeprazole (2), Tramadol (2), Diclofenac (3), Irbesartan (1), Vitamin D (1), Bendroflumethiazide (1), Simvastatin (2), Co-codamol (3), Metformin (7), Ramipril (2), Aspirin (1), Doxazosin (2), Lisinopril (1), Gabapentin (1), Paracetamol (4), Ventolin (1), Arcoxia (2), Co-dydramol (3), Gliclazide (3), Atorvastatin (4), Pioglitazone (1), Trimethoprim (1), Lipitor (1), Allopurinol (1), Voltarol (1), Buprenorphine (1), Dihydrocodeine (2), Glucophage (1), Lanus (1), Novorapid (1), Amoxycillin (1), Clarithromycin (1), Losartan (1), Frusemide (1), Vitamin B12 (1), Humalog (2), Exenatide (1), Glucaxide (1), Glargine (1), Citalopram (2), Colecalcif (1), Metatone (1), Paroxitene, (1), Fenobrate (1), Ferrous fu (3), Bisacodyl (1), Pregabalin (1),
Recent Steroid Therapy (n)	No (30), Yes (5)	No (20), Yes (2)
Steroid Treatment (n)	Depo-Medro (2), Pred (2), Fluticason (1)	Methylpred. (1), Depo-Medro (1)
DMARD Treatment (n)	MTX (1), MTX/ASA (10), MTX/ASA/HCQ (5), ASA (1), ASA/HCQ (2), HCQ (1), MTX/HCQ/LEF (1), MTX/HCQ (12)	MTX (4), MTX/ASA/HCQ (2), MTX/ASA (6), MTX/HCQ (2), ASA (2), ASA/HCQ (1), HCQ (1), ASA/LEF (1)
ESR (mm/hr)	42 (±34)	45 (±26)
CRP (mg/l)	22 (±34)	31 (±35)
CCP (UI/L)	197 (±218)	164 (±200)
RF (UI/L)	119 (±187)	272 (±227)
Tiredness VAS (1-100)	49 (±31)	51 (±32)
Pain VAS (1-100)	61 (±28)	55 (±26)
Pt. VAS Global Health (1-100)	62 (±29)	62 (±30)
Physician VAS Global Assess. (1-100)	60 (±24)	60 (±25)

Tender Joints Baseline (Number/28)	15 (±8)	13 (±8)
Swollen Joints Baseline (Number/28)	9 (±6)	6 (±5)
HAQ (Max 38)	1.46 (±0.75)	1.85 (±0.68)
DAS28 (0-10)	6.03 (±1.48)	5.67 (±1.20)
Delta DAS28 (difference between 2 time points	-2.59 (±1.18)	0.14 (±1.0)
Response (1)	Responder (36)	Non-responder (22)
Response (2)	Good (19), Moderate (17)	Non-responder (22)
US Synovial Thickness (12max) (Number/36)	2 (±0)	
US Power Doppler (12max) (Number/36)	1.5 (±0.5)	
US Synovial Thickness (Biopsied Joint) (Number/3)	13 (±1)	
US Power Doppler (Biopsied Joint) (Number/3)	4 (±1)	
Radiographic Erosion (n)	No (20), Yes (6)	No (11), Yes (4)

Erythrocyte sedimentation rate = ESR, C-reactive protein = CRP, Anti-cyclic citrullinated peptide = CCP, rheumatoid factor =RF, VAS = visual analogue scale, HAQ = Health Assessment Questionnaire, DAS28 = disease activity score-28, US = ultrasound, MTX = methotrexate, ASA = aspirin, HCQ = hydroxychloroquine, LEF = Leflunomide.

Supplementary Table 5: Baseline peripheral blood lipid mediator profiles in RA patient cohort 2.

	Q1	Q3	DMARD Responders			DMARD Non-Responders		
DHA bioactive metabolome			Mean	±	SEM	Mean	±	SEM
RvD1	375	233	0.31	±	0.19	0.50	±	0.17
RvD2	375	141	0.08	±	0.04	16.96	±	11.42
RvD3	375	147	0.01	±	0.01	0.10	±	0.05
RvD4	375	101	0.93	±	0.27	1.98	±	0.51
RvD5	359	199	0.15	±	0.04	2.39	±	1.41
RvD6	359	101	0.18	±	0.03	1.72	±	0.78
17R -RvD1	375	233	0.49	±	0.12	0.68	±	0.23
17R -RvD3	375	147	0.01	±	0.00	0.02	±	0.02
PD1	359	153	0.38	±	0.06	0.92	±	0.28
PDX	359	153	0.12	±	0.02	1.47	±	0.60
17R-PD1	359	153	0.04	±	0.01	0.65	±	0.34
22-OH-PD1	375	153	0.09	±	0.03	0.66	±	0.35
PCTR1	650	231	0.00	±	0.00	0.00	±	0.00
PCTR2	521	231	0.17	±	0.06	0.13	±	0.08
PCTR3	464	231	0.36	±	0.14	0.00	±	0.00
MaR1	359	221	0.44	±	0.14	3.27	±	0.81
MaR2	359	191	0.08	±	0.03	1.43	±	0.74
7S,14S-diHDHA	359	221	0.67	±	0.17	2.70	±	0.70
4S,14S-diHDHA	359	101	0.65	±	0.09	3.79	±	2.12
22-OH-MaR1	375	221	0.74	±	0.26	6.55	±	1.98
14-oxo-MaR1	357	248	0.04	±	0.02	0.02	±	0.01
MCTR1	650	191	0.00	±	0.00	0.00	±	0.00
MCTR2	521	191	0.44	±	0.12	2.15	±	0.81
MCTR3	464	191	0.29	±	0.12	1.83	±	1.14
n-3 DPA bioactive metabolome								
RvT1	377	193	0.48	±	0.45	0.00	±	0.00
RvT2	377	197	2.07	±	0.18	2.40	±	1.36
RvT3	377	197	0.32	±	0.11	0.43	±	0.13
RvT4	361	211	1.74	±	1.03	0.93	±	0.34
$RvD1_{n-3 DPA}$	377	215	0.78	±	0.21	0.74	±	0.50
$RvD2_{n-3 DPA}$	377	261	0.36	±	0.25	0.17	±	0.06
$RvD5_{n-3DPA}$	361	199	0.49	±	0.22	2.69	±	0.90
$PD1_{n-3 DPA}$	361	155	0.06	±	0.03	0.10	±	0.06
10S, 17S-diHDPA	361	155	0.08	±	0.03	0.12	±	0.06
$MaR1_{n-3 DPA}$	361	223	0.05	±	0.03	1.53	±	0.69
EPA bioactive metabolome								
RvE1	349	195	0.24	±	0.08	0.01	±	0.01
RvE2	333	159	1.58	±	0.27	5.37	±	1.62
RvE3	333	201	0.21	±	0.06	0.18	±	0.10
AA bioactive metabolome								
LXA_4	351	115	0.02	±	0.01	0.41	±	0.16
LXB4	351	221	0.80	±	0.49	1.86	±	0.83
15R-LXA_4	351	115	0.65	±	0.31	3.82	±	0.82
15R-LXB_4	351	221	1.39	±	0.30	0.44	±	0.17
5S,15S-diHETE	335	235	2.05	±	0.44	5.87	±	3.78
LTB_4	335	195	18.04	±	4.20	66.04	±	14.51
5S,12S-diHETE	335	195	0.15	±	0.07	10.51	±	2.95
6-trans-LTB_4	335	195	0.72	±	0.14	4.79	±	0.98
12-epi-6-trans-LTB_4	335	195	0.63	±	0.17	9.97	±	2.10
20-OH-LTB_4	351	195	10.92	±	3.42	34.65	±	7.93
20-COOH-LTB_4	369	195	1.43	±	0.48	7.26	±	1.89
LTC_4	626	189	51.99	±	17.43	14.45	±	3.07
LTD_4	497	189	14.88	±	4.12	8.14	±	1.89
LTE_4	440	189	91.54	±	23.83	32.76	±	6.85
PGD_2	351	189	0.87	±	0.15	5.84	±	1.39
PGE_2	351	189	1.30	±	0.31	7.13	±	1.72
PGF_{2a}	353	193	2.04	±	0.62	23.01	±	4.64
TXB_2	369	169	53.28	±	13.57	45.37	±	16.21

Results are expressed pg/ml. n = 36 for DMARD Responders and n = 22 DMARD Non-Responders

Supplementary Table 6: Chiral chromatography of baseline plasma from DMARD responders and non-responders demonstrates higher concentrations of the S-monohydroxylated enantiomers.

	Responders		Non-Responders	
	R-Enantiomer *Relative abundance (%)*	S-Enantiomer *Relative abundance (%)*	R-Enantiomer *Relative abundance (%)*	S-Enantiomer *Relative abundance (%)*
17-HDHA	45.48 ± 2.48	54.52 ± 2.48	44.89 ± 1.69	55.11 ± 1.69
14-HDHA	6.71 ± 1.03	93.29 ± 1.03	19.84 ± 18.50	80.16 ± 18.50
7-HDHA	31.64 ± 3.13	68.36 ± 3.13	35.20 ± 4.01	64.80 ± 4.01
17-HDPA	16.16 ± 6.76	83.84 ± 6.76	16.16 ± 6.76	83.84 ± 6.76
14-HDPA	6.90 ± 1.63	93.10 ± 1.63	6.90 ± 1.63	93.10 ± 1.63
7-HDPA	-	-	-	-
15-HEPE	34.31 ± 5.59	65.69 ± 5.59	34.31 ± 5.59	65.69 ± 5.59
5-HEPE	24.34 ± 8.33	75.66 ± 8.33	24.34 ± 8.33	75.66 ± 8.33
15-HETE	4.44 ± 2.64	95.56 ± 2.64	4.44 ± 2.64	95.56 ± 2.64
5-HETE	11.27 ± 2.37	88.73 ± 2.37	11.27 ± 2.37	88.73 ± 2.37

- = below detection limits

Supplementary Table 7: Peripheral blood lipid mediator profiles in patients with RA after 6 months of DMARD treatment.

	Q1	Q3	DMARD Responders			DMARD Non-Responders		
DHA bioactive metabolome			Mean	±	SEM	Mean	±	SEM
RvD1	375	233	0.09	±	0.06	0.60	±	0.19
RvD2	375	141	0.97	±	0.44	0.18	±	0.13
RvD3	375	147	0.13	±	0.05	0.02	±	0.02
RvD4	375	101	1.38	±	0.41	0.93	±	0.26
RvD5	359	199	0.30	±	0.12	0.32	±	0.11
RvD6	359	101	0.52	±	0.10	0.55	±	0.16
17R -RvD1	375	233	0.13	±	0.07	0.27	±	0.13
17R -RvD3	375	147	0.07	±	0.04	0.10	±	0.07
PD1	359	153	1.14	±	0.19	0.78	±	0.27
PDX	359	153	0.92	±	0.36	9.23	±	3.38
17R-PD1	359	153	0.64	±	0.51	5.98	±	2.01
22-OH-PD1	375	153	0.07	±	0.04	0.06	±	0.05
PCTR1	650	231	2.25	±	1.59	0.00	±	0.00
PCTR2	521	231	0.39	±	0.14	0.05	±	0.05
PCTR3	464	231	0.00	±	0.00	0.00	±	0.00
MaR1	359	221	6.82	±	1.39	5.83	±	1.30
MaR2	359	191	0.64	±	0.38	0.00	±	0.00
7S,14S-diHDHA	359	221	2.21	±	0.78	2.79	±	0.69
4S,14S-diHDHA	359	101	3.89	±	3.28	1.51	±	0.37
22-OH-MaR1	375	221	2.21	±	0.95	2.07	±	1.78
14-oxo-MaR1	357	248	0.32	±	0.32	2.35	±	0.86
MCTR1	650	191	0.00	±	0.00	0.00	±	0.00
MCTR2	521	191	0.97	±	0.23	0.28	±	0.19
MCTR3	464	191	3.32	±	0.98	4.71	±	1.61
n-3 DPA bioactive metabolome								
RvT1	377	193	0.34	±	0.13	0.23	±	0.12
RvT2	377	197	0.04	±	0.04	0.16	±	0.10
RvT3	377	197	0.00	±	0.00	0.00	±	0.00
RvT4	361	211	0.31	±	0.09	0.93	±	0.27
$RvD1_{n\text{-}3\ DPA}$	377	143	1.19	±	0.31	1.02	±	0.27
$RvD2_{n\text{-}3\ DPA}$	377	261	1.54	±	0.27	1.68	±	0.58
$RvD5_{n\text{-}3DPA}$	361	199	1.26	±	0.21	1.38	±	0.28
$PD1_{n\text{-}3\ DPA}$	361	155	0.37	±	0.08	0.34	±	0.11
10S, 17S-diHDPA	361	155	0.28	±	0.07	0.16	±	0.11
$MaR1_{n\text{-}3\ DPA}$	361	249	0.02	±	0.02	0.10	±	0.07
EPA bioactive metabolome								
RvE1	349	161	0.09	±	0.07	0.00	±	0.00
RvE2	333	199	5.26	±	1.02	3.62	±	1.44
RvE3	333	201	0.87	±	0.26	0.90	±	0.23
AA bioactive metabolome								
LXA_4	351	115	0.11	±	0.03	0.08	±	0.03
LXB4	351	221	0.17	±	0.08	0.36	±	0.23
$15R\text{-}LXA_4$	351	115	0.76	±	0.15	1.78	±	0.54
$15R\text{-}LXB_4$	351	221	5.11	±	0.60	9.37	±	1.39
5S,15S-diHETE	335	115	26.99	±	7.61	26.54	±	3.89
LTB_4	335	195	52.21	±	10.73	108.07	±	50.98
5S,12S-diHETE	335	195	1.57	±	0.69	0.85	±	0.33
$6\text{-}trans\text{-}LTB_4$	335	195	1.67	±	0.28	5.35	±	3.63
$12\text{-}epi\text{-}6\text{-}trans\text{-}LTB_4$	335	195	1.95	±	0.33	4.63	±	2.24
$20\text{-}OH\text{-}LTB_4$	351	195	24.47	±	7.22	42.62	±	18.36
$20\text{-}COOH\text{-}LTB_4$	369	195	0.16	±	0.03	0.42	±	0.33
LTC_4	626	189	13.21	±	4.65	20.10	±	6.85
LTD_4	497	189	6.86	±	1.69	5.65	±	1.31
LTE_4	440	189	41.48	±	12.00	87.57	±	44.40
PGD_2	351	189	7.48	±	1.87	12.26	±	5.93
PGE_2	351	189	11.10	±	3.55	11.29	±	4.59
PGF_{2a}	353	193	11.24	±	2.30	13.45	±	3.42

TXB_2	369	169	190.70 ± 72.02	168.15 ± 85.85

Results are expressed pg/ml. n = 27 for DMARD Responders and n = 17 for DMARD Non-Responders.

Supplementary Table 8: Chiral chromatography of plasma 6 months post treatment initiation from DMARD responders and non-responders demonstrates higher concentrations of the S-monohydroxylated enantiomers

	Responders		Non-Responders	
	R-Enantiomer *Relative abundance (%)*	S-Enantiomer *Relative abundance (%)*	R-Enantiomer *Relative abundance (%)*	S-Enantiomer *Relative abundance (%)*
17-HDHA	66.80 ± 3.97	33.20 ± 3.97	58.22 ± 11.53	41.78 ± 11.53
14-HDHA	9.57 ± 2.76	90.43 ± 2.76	10.32 ± 4.39	89.68 ± 4.39
7-HDHA	38.47 ± 10.90	61.53 ± 10.90	32.37 ± 8.50	67.63 ± 8.50
17-HDPA	-	-	-	-
14-HDPA	3.25 ± 0.32	96.75 ± 0.32	2.06 ± 0.40	97.94 ± 0.40
7-HDPA	-	-	-	-
15-HEPE	77.12 ± 6.97	22.88 ± 6.97	67.06 ± 10.51	32.94 ± 10.51
5-HEPE	18.30 ± 3.55	81.70 ± 3.55	17.86 ± 3.28	82.14 ± 3.28
15-HETE	48.48 ± 18.21	51.52 ± 18.21	15.87 ± 6.60	84.13 ± 6.60
5-HETE	15.82 ± 0.75	84.18 ± 0.75	13.51 ± 1.81	86.49 ± 1.81

- = below detection limits

Supplementary Table 9: Mass Spectrometer settings for Multiple Reaction Monitoring and Enhance product Ion for AB Sciex 5500 Q TRAP.

AB Sciex 5500 Q TRAP		
**Parameter**	**MRM**	**EPI**
Curtain Gas	35	35
Collision Gas	Medium	Medium
Ion Spray Voltage	-3500	-3500
Temperature	550 °C	550 °C
Ion Source Gas 1	30	30
Ion Source Gas 2	75	75

Supplementary Table 10: Mass Spectrometer settings for Multiple Reaction Monitoring and Enhance product Ion for AB Sciex 6500+ Q TRAP.

AB Sciex 6500+ Q TRAP		
**Parameter**	**MRM**	**EPI**
Curtain Gas	30	30
Collision Gas	Medium	Medium
Ion Spray Voltage	-4500	-4500
Temperature	440 °C	440 °C
Ion Source Gas 1	45	45
Ion Source Gas 2	70	70

Supplementary Table 11: Signal repeatability for MRM transitions employed in the quantitation of lipid mediators – SCIEX 5500.

			% variability		
Compound	Q1	Q3	0.5 pg	1 pg	5 pg
DHA Metabolome					
RvD1	375	233	12%	4%	3%
RvD2	375	141	4%	6%	5%
RvD3	375	147	5%	3%	7%
RvD4	375	101	2%	4%	2%
RvD5	359	199	6%	9%	2%
RvD6	359	101	5%	3%	3%
17R -RvD1	375	233	7%	6%	7%
17R -RvD3	375	147	9%	1%	7%
PD1	359	153	7%	8%	3%
PDX	359	153	3%	5%	1%
17R-PD1	359	153	5%	1%	9%
22-OH-PD1	375	153	2%	2%	3%
MaR1	359	221	19%	6%	15%
MaR2	359	191	3%	1%	2%
7S,14S-diHDHA	359	221	5%	6%	3%
4S,14S-diHDHA*	359	101	6%	5%	2%
22-OH-MaR1	375	221	N.D.	N.D.	N.D.
14-oxo-MaR1	357	248	N.D.	N.D.	N.D.
n-3 DPA Metabolome					
RvT1	377	193	5%	8%	3%
RvT2	377	197	3%	5%	5%
RvT3	377	197	4%	5%	5%
RvT4	361	211	5%	14%	6%
$RvD1_{n\text{-}3\ DPA}$	377	143	11%	6%	4%
$RvD2_{n\text{-}3\ DPA}$	377	261	9%	5%	1%
$RvD5_{n\text{-}3\ DPA}$	361	199	10%	5%	5%
$PD1_{n\text{-}3\ DPA}$	361	183	3%	5%	5%
10S, 17S-diHDPA*	361	183	3%	5%	5%
$MaR1_{n\text{-}3\ DPA}$	361	249	10%	9%	4%
EPA Metabolome					
RvE1	349	161	4%	6%	4%
RvE2	333	199	17%	9%	6%
RvE3	333	201	N.D.	N.D.	N.D.
AA Metabolome					
LXA_4	351	115	7%	3%	4%
LXB_4	351	221	10%	4%	7%
5S,15S-diHETE	335	115	14%	11%	6%
15-epi-LXA_4	351	115	6%	3%	3%
15-epi-LXB_4	351	221	6%	1%	7%
LTB_4	335	195	2%	4%	6%
5S,12S-diHETE*	335	195	2%	4%	6%
6-trans-LTB_4*	335	195	2%	4%	6%
12-epi-6-trans-LTB_4*	335	195	2%	4%	6%
20-OH-LTB_4	335	195	4%	2%	5%
20-COOH-LTB_4	351	195	5%	2%	7%
PGD_2	351	189	4%	5%	2%
PGE_2	351	189	5%	5%	3%
$PGF_{2\alpha}$	353	193	6%	4%	5%

TXB_2	369	169	10%	7%	17%
d_4-PGE_2	355	193	17%	8%	2%
d_5-LXA_4	356	115	9%	3%	3%
d_5-RvD2	380	141	19%	9%	3%
d_4-LTB_4	339	197	9%	8%	6%

Lipid mediator were injected at the indicated concentrations and signal was quantified using a Sciex 5500 mass spectrometer. AUC for 4 distinct injections were determined and average calculated. Results are absolute percent variation for each transition. * Repeatability was determined using RvD6 for 4S,4S-diHDHA, $PD1_{n\text{-}3\ DPA}$ for 10S, 17S-diHDPA and LTB_4 for 5S,12S-diHETE, 6-trans-LTB_4, 12-epi-6-trans-LTB_4. N.D. = not determined

Supplementary Table 12: Signal repeatability for MRM transitions employed in the quantitation of lipid mediators – SCIEX 6500+.

			% variability		
Compound	Q1	Q3	0.5 pg	1 pg	5 pg
DHA Metabolome					
RvD1	375	233	11%	9%	5%
RvD2	375	141	12%	9%	3%
RvD3	375	147	4%	4%	1%
RvD4	375	101	2%	3%	2%
RvD5	359	199	3%	6%	3%
RvD6	359	101	2%	4%	1%
17R -RvD1	375	233	7%	3%	1%
17R -RvD3	375	147	2%	9%	1%
PD1	359	153	8%	2%	2%
PDX	359	153	2%	3%	3%
17R-PD1	359	153	3%	4%	2%
22-OH-PD1	375	153	1%	1%	1%
PCTR1	650	231	7%	8%	9%
PCTR2	521	231	5%	5%	4%
PCTR3	464	231	7%	2%	13%
MaR1	359	221	9%	14%	15%
MaR2	359	191	2%	2%	1%
7S,14S-diHDHA	359	221	6%	5%	7%
4S,14S-diHDHA	359	101	3%	3%	1%
22-OH-MaR1	375	221	N.D.	N.D.	N.D.
14-oxo-MaR1	357	248	N.D.	N.D.	N.D.
MCTR1	650	191	4%	9%	9%
MCTR2	521	191	2%	4%	4%
MCTR3	464	191	3%	4%	7%
n-3 DPA Metabolome					
RvT1	377	193	10%	6%	2%
RvT2	377	197			
RvT3	377	197			
RvT4	361	211	9%	6%	6%
$RvD1_{n\text{-}3\ DPA}$	377	215	6%	5%	3%
$RvD2_{n\text{-}3\ DPA}$	371	261	9%	16%	13%
$RvD5_{n\text{-}3\ DPA}$	361	199	9%	10%	3%
$PD1_{n\text{-}3\ DPA}$	361	183			
10S, 17S-diHDPA	361	183			
$MaR1_{n\text{-}3\ DPA}$	361	249	1%	10%	6%
EPA Metabolome					
RvE1	349	195	5%	5%	2%
RvE2	333	159	5%	10%	4%
RvE2	333	201	N.D.	N.D.	N.D.
AA Metabolome					
LXA_4	351	115	13%	6%	2%
LXB_4	351	221	12%	4%	7%
5S,15S-diHETE	335	235	15%	16%	8%
15-epi-LXA_4	351	115	5%	4%	4%
15-epi-LXB_4	351	221	8%	4%	6%
LTB_4	335	195	4%	3%	2%
5S,12S-diHETE*	335	195	4%	3%	2%
6-trans-LTB_4*	335	195	4%	3%	2%
12-epi-6-trans-LTB_4*	335	195	4%	3%	2%
20-OH-LTB_4	351	195	3%	3%	1%
20-COOH-LTB_4	369	195	6%	10%	4%

LTC_4	626	189	5%	7%	7%
LTD_4	497	189	3%	1%	12%
LTE_4	440	189	4%	3%	8%
PGD_2	351	189	6%	5%	1%
PGE_2	351	189	5%	3%	4%
$PGF_{2\alpha}$	353	193	11%	3%	1%
TXB_2	369	169	8%	8%	9%
d_4-PGE_2	355	193	6%	8%	2%
d_5-LXA_4	356	115	5%	4%	2%
d_5-RvD2	380	141	14%	10%	3%
d_4-LTB_4	339	197	13%	5%	1%
d_5-LTC_4	631	194	6%	2%	12%
d_5-LTD_4	502	194	6%	3%	13%
d_5-LTE_4	445	194	3%	3%	3%

Lipid mediator were injected at the indicated concentrations and signal was quantified using a Sciex 6500+ mass spectrometer. AUC for 4 distinct injections were determined and average calculated. Results are absolute percent variation for each transition. * Repeatability was determined using a RvD6 for 4S,4S-diHDHA, $PD1_{n\text{-}3\ DPA}$ for 10S, 17S-diHDPA and LTB_4 for 5S,12S-diHETE, 6-trans-LTB_4, 12-epi-6-trans-LTB_4. N.D. = not determined
