# Supplementary material for: Blood pro-resolving mediators are linked with synovial pathology and are predictive of DMARD responsiveness in rheumatoid arthritis
Source: Nat Commun. 2020 Oct 27;11:5420. doi: 10.1038/s41467-020-19176-z (PMC7591509; doi:10.1038/s41467-020-19176-z)
Supplement: Supplementary file 3 — Reporting Summary [file 41467_2020_19176_MOESM3_ESM.pdf]

nature research

Corresponding author(s): Prof Jesmond Dalli

Last updated by author(s): 09/09/2020

Reporting Summary

Nature Research wishes to improve the reproducibility of the work that we publish. This form provides structure for consistency and transparency in reporting. For further information on Nature Research policies, see our Editorial Policies and the Editorial Policy Checklist.

Statistics

For all statistical analyses, confirm that the following items are present in the figure legend, table legend, main text, or Methods section.

n/a	Confirmed	
☐	☒	The exact sample size (n) for each experimental group/condition, given as a discrete number and unit of measurement
☐	☒	A statement on whether measurements were taken from distinct samples or whether the same sample was measured repeatedly
☐	☒	The statistical test(s) used AND whether they are one- or two-sided<br>*Only common tests should be described solely by name; describe more complex techniques in the Methods section.*
☐	☒	A description of all covariates tested
☐	☐	A description of any assumptions or corrections, such as tests of normality and adjustment for multiple comparisons
☐	☒	A full description of the statistical parameters including central tendency (e.g. means) or other basic estimates (e.g. regression coefficient) AND variation (e.g. standard deviation) or associated estimates of uncertainty (e.g. confidence intervals)
☐	☒	For null hypothesis testing, the test statistic (e.g. F, t, r) with confidence intervals, effect sizes, degrees of freedom and P value noted<br>*Give P values as exact values whenever suitable.*
☒	☐	For Bayesian analysis, information on the choice of priors and Markov chain Monte Carlo settings
☐	☒	For hierarchical and complex designs, identification of the appropriate level for tests and full reporting of outcomes
☒	☐	Estimates of effect sizes (e.g. Cohen's d, Pearson's r), indicating how they were calculated

Our web collection on statistics for biologists contains articles on many of the points above.

Software and code

Policy information about availability of computer code

Data collection	Analyst v1.63 (Lipid Mediator profiling), Illumina HiSeq2500 (sequencing)
Data analysis	Prism v8 (Lipid mediator profiling, Flow cytometry), R Software v3.5.1 and RStudio v1.1.456(Lipid mediator profiling + Sequencing + Machine Learning), Analyst v1.63 (Lipid mediator profiling), Cytoscape v3.7.1 (Lipid meidator profiling), Kallisto v0.43.0 (Sequencing), MetaboAnalyst v4.01 (Lipid mediator profiling), SIMCA v14.1 (Lipid mediator profiling), Microsoft Excel Professional Plus 2016.<br><br>All relevant codes used in this study are available in the following Github repository (section R Scripts):<br>https://github.com/eagomezc/2019_Machine_Learning_DMARD_in_RA_patients/tree/master/b_R_Scripts

For manuscripts utilizing custom algorithms or software that are central to the research but not yet described in published literature, software must be made available to editors and reviewers. We strongly encourage code deposition in a community repository (e.g. GitHub). See the Nature Research guidelines for submitting code & software for further information.

Data

Policy information about availability of data

All manuscripts must include a data availability statement. This statement should provide the following information, where applicable:

- Accession codes, unique identifiers, or web links for publicly available datasets
- A list of figures that have associated raw data
- A description of any restrictions on data availability

All relevant dataset generated during and/or analyzed in current study (Figures 1-4, and Supplemental Figures 5, 7, 8, 13, 14) are available in the following public Github repository (Section Data):
https://github.com/eagomezc/2019_Machine_Learning_DMARD_in_RA_patients/tree/master/a_Data

RNA-Seq data (Supplemental Figure 7) are uploaded to ArrayExpress and are accessible via accession E-MTAB-6141:
https://www.ebi.ac.uk/arrayexpress/experiments/E-MTAB-6141/

Field-specific reporting

Please select the one below that is the best fit for your research. If you are not sure, read the appropriate sections before making your selection.

- [x] Life sciences
- [ ] Behavioural & social sciences
- [ ] Ecological, evolutionary & environmental sciences

For a reference copy of the document with all sections, see nature.com/documents/nr-reporting-summary-flat.pdf

Life sciences study design

All studies must disclose on these points even when the disclosure is negative.

Sample size	Sample size for lipid mediator analysis and model building was evaluated using learning curves in Random Forests by evaluating the change in predictive accuracy and standard deviation across the 100 models.
Data exclusions	From the exploratory analysis (PLS-DA analysis), two samples were removed for showing outlier concentrations of TXB2, which likely reflected coagulation during sample collection and an additional sample was removed due to lack of clinical records.
Replication	Machine learning models were established using an independent cohort of samples and then these models were evaluated (corroborate the accuracy of the models) using a second independent cohort of samples. Lipid mediator profiling, PBMC counts and differential gene expression analysis were not replicated.
Randomization	All patients were disease modifying anti-rheumatic drugs (DMARDs) and steroid-naïve, had symptoms duration less than 12 months and fulfilled the ACR/EULAR 2010 classification criteria for RA. RA individuals were categorised into three pathotypes based on histological classification of synovial tissue: Lympho-myeloid, Diffuse-Myeloid and pauci-immune Fibroid. Patients were treated with DMARDs. Response status after 6 months of mixed DMARD therapy was determined (Responder and Non-Responder groups) by EULAR response criteria based on DAS28-ESR.
Blinding	Investigators were blinded for data acquisition (lipid mediator profiling and RNA sequencing) and data analysis. For model building blinding was not possible since we used supervised machine learning models and we determined statistical significance differences comparing the two groups (Responder and Non-Responders to DMARD therapy).

Reporting for specific materials, systems and methods

We require information from authors about some types of materials, experimental systems and methods used in many studies. Here, indicate whether each material, system or method listed is relevant to your study. If you are not sure if a list item applies to your research, read the appropriate section before selecting a response.

Materials & experimental systems

n/a	Involved in the study	
x		Antibodies
x		Eukaryotic cell lines
x		Palaeontology and archaeology
x		Animals and other organisms
	x	Human research participants
	x	Clinical data
x		Dual use research of concern

Methods

n/a	Involved in the study	
x		ChIP-seq
x		Flow cytometry
x		MRI-based neuroimaging

Human research participants

Policy information about studies involving human research participants

Population characteristics	This information is provided in Supplemental Tables 1 and 4 for patient blood. For healthy volunteers these were all of an age between 20 and 39 years, that declared not taking NSAIDS for at least 14 days, caffeine and alcohol for at least 24h and fatty fish for 48h.
Recruitment	Participants were not directly recruited into the study
Ethics oversight	Volunteers gave written consent in accordance with a Queen Mary Research Ethics Committee (QMREC 2014:61) and the Helsinki declaration

Note that full information on the approval of the study protocol must also be provided in the manuscript.

Clinical data

Policy information about clinical studies

All manuscripts should comply with the ICMJE guidelines for publication of clinical research and a completed CONSORT checklist must be included with all submissions.

Clinical trial registration	REC 05/Q0703/198 by King's College Hospital Research Ethics Committee. All relevant information about the Pathobiology of Early Arthritis Cohort (PEAC) can be found in: http://www.peac-mrc.mds.qmul.ac.uk/docs.php
Study protocol	http://www.peac-mrc.mds.qmul.ac.uk/docs/PEAC_PROTOCOL_Protected.pdf
Data collection	RA patients fulfilling 2010 American College of Rheumatology/European League Against Rheumatism (EULAR) Classification Criteria, with clinically defined synovitis and symptom duration less than 12 months, were enrolled as part of the 'Pathobiology of Early Arthritis Cohort' (PEAC, http://www.peac-mrc.mds.qmul.ac.uk) at three UK Academic Centres: Queen Mary University of London/ Barts Health NHS trust, University of Glasgow and University of Birmingham. All patients were naïve to steroid and DMARD therapy.<br><br>Clinical data was collected at recruitment and every 6 months for a period of 2 years. Health assessment questionnaire, European Quality of life score, fatigue scores and DAS28 score was recorded as normally done in the RA clinic of Queen Mary University of London.<br><br>Within 2 weeks of recruitment for each patient, patients underwent a ultrasound (US)-guided synovial biopsy (figure 1A) procedure we pioneered of a clinically active joint selected according to a previously defined algorithm to ensure maximal synovial tissue retrieval; a minimum of 12 synovial biopsies were stored for subsequent analysis at the William Harvey research Institute (six for histological analysis and six for RNA extraction), and patients then commenced on standard DMARD therapy and/or low-dose corticosteroid. A treat-to-target approach to therapy escalation was followed aiming for low disease activity score-28 (DAS28) <3.2. Patients failing DMARD therapy were commenced on biological therapy according to the UK National Institute for Clinical Excellence prescribing algorithm for RA patients if they continued to have a DAS28 >5.1 at 6 months.<br><br>Ultrasonographic images were collected at the time of biopsy for both the individual biopsied joint and the global joint score: first to fifth metacarpophalangeal (MCP) joints and midline, radial and ulnar views of both wrist joints. Images subsequently underwent semiquantitative assessment by a single blinded (to clinical/histological data) assessor for both synovial thickening (ST) and power Doppler (PD) activity according to standard EULAR-OMERACT (Outcome Measures in Rheumatology) US synovitis scores (grade 0–3).7 The mean global ST and PD scores including the maximal score for the wrist joint were then determined. A second biopsy was performed and serum was collected after 6 months for the above test to be repeated.<br><br>Plain radiographs of the hands and feet performed at baseline and 12-month follow-up were scored in time sequential order according to the van der Heijde modified Sharp score (SHSS) by a single reader blinded to all clinical/histological data.
Outcomes	The purpose of PEAC is to develop a biomedical resource with a comprehensive collection of clinical, radiological, and tissue samples from patients with early onset inflammatory arthritis.<br><br>The primary measure of disease activity in this study is the DAS28(ESR). The components of the DAS28(ESR) are the number of tender joints (28 joint count), the number of swollen joints (28 joint count), the Patient Global Health Index Score (100 mm VAS), and the ESR (mm/hr).<br>DAS28 values >5.1 correspond to a high disease activity; values between 3.2 and 5.1 indicate moderate disease activity; values between 2.6 and 3.2 define low disease activity, and values < 2.6 represent remission.<br><br>Clinical response will be assessed according to the EULAR response criteria
